# Regional brain morphology and current antidepressant use: findings from 32 international cohorts from the ENIGMA major depressive disorder working group

**DOI:** 10.1038/s41380-025-03310-8

**Published:** 2025-11-03

**Authors:** Chaira Serrarens, Yara J. Toenders, Elena Pozzi, André Aleman, Nina Alexander, Zeynep Başgöze, Vladimir Belov, Klaus Berger, Katharina Brosch, Robin Bülow, Geraldo Filho Busatto, Liliana P. Capitão, Colm G. Connolly, Baptiste Couvy-Duchesne, Kathryn R. Cullen, Udo Dannlowski, Christopher G. Davey, Greig I. de Zubicaray, Danai Dima, Katharina Dohm, Verena Enneking, Tracy Erwin-Grabner, Ulrika Evermann, Cynthia H. Y. Fu, Paola Fuentes-Claramonte, Beata R. Godlewska, Ali Saffet Gonul, Ian H. Gotlib, Roberto Goya-Maldonado, Hans J. Grabe, Nynke A. Groenewold, Dominik Grotegerd, Oliver Gruber, Tim Hahn, Geoffrey Hall, Ben J. Harrison, Walter Heindel, Marco Hermesdorf, Tiffany C. Ho, Naho Ichikawa, Eri Itai, Neda Jahanshad, Hamidreza Jamalabadi, Alec J. Jamieson, Andreas Jansen, Tilo Kircher, Bonnie Klimes-Dougan, Bernd Krämer, Axel Krug, Thomas M. Lancaster, Elisabeth J. Leehr, Meng Li, David E. J. Linden, Frank MacMaster, Katie L. McMahon, Sarah E. Medland, David M. A. Mehler, Susanne Meinert, Benson Mwangi, Igor Nenadić, Go Okada, Yasumasa Okamoto, Nils Opel, Julia-Katharina Pfarr, Edith Pomarol-Clotet, Maria J. Portella, Ronny Redlich, Liesbeth Reneman, Jonathan Repple, Kai Ringwald, Elena Rodriguez-Cano, Pedro G. P. Rosa, Matthew D. Sacchet, Philipp G. Sämann, Raymond Salvador, Anouk Schrantee, Hotaka Shinzato, Kang Sim, Egle Simulionyte, Jair C. Soares, Dan J. Stein, Frederike Stein, Benjamin Straube, Lachlan T. Strike, Florian Thomas-Odenthal, Sophia I. Thomopoulos, Paul M. Thompson, Marie-Jose van Tol, Paula Usemann, Aslihan Uyar, Nic van der Wee, Steven van der Werff, Yolanda Vives-Gilabert, Henry Völzke, Martin Walter, Sarah Whittle, Katharina Wittfeld, Adrian Wroblewski, Mon-Ju Wu, Tony T. Yang, Giovana B. Zunta-Soares, Dick J. Veltman, Lianne Schmaal, Laura S. van Velzen

**Affiliations:** 1 Department of Psychiatry and Neuropsychology, Mental Health and Neuroscience Research Institute, Maastricht University, Maastricht, the Netherlands; 2 Changes GGZ, Breda, the Netherlands; 3 Orygen, Parkville, VIC, Australia; 4 Centre for Youth Mental Health, University of Melbourne, Melbourne, VIC, Australia; 5 Leiden University, Developmental and Educational Psychology, Leiden, the Netherlands; 6 Erasmus University Rotterdam, Erasmus School of Social and Behavioural Sciences, Rotterdam, the Netherlands; 7 University of Groningen, Cognitive Neuroscience Center, Department of Biomedical Sciences of Cells & Systems, University Medical Center Groningen, Groningen, the Netherlands; 8 Philipps-Universität Marburg, Faculty of Medicine, Department of Psychiatry and Psychotherapy, Marburg, Germany; 9 Center for Mind, Brain and Behavior, University of Marburg, Marburg, Germany; 10 Department of Psychiatry and Behavioral Sciences, University of Minnesota, Minneapolis, MN USA; 11 Laboratory of Systems Neuroscience and Imaging in Psychiatry (SNIP-Lab), University Medical Center Göttingen (UMG), Göttingen, Germany; 12 Institute of Epidemiology and Social Medicine, University of Muenster, Muenster, Germany; 13 Institute of Behavioral Science, Feinstein Institutes for Medical Research, Manhasset, USA; 14 Institute for Diagnostic Radiology and Neuroradiology, University Medicine Greifswald, Greifswald, Germany; 15 German Center for Cardiovascular Research, Partner Site Greifswald, Greifswald, Germany; 16 Laboratory of Psychiatric Neuroimaging (LIM-21), Departamento e Instituto de Psiquiatria, Hospital das Clinicas HCFMUSP, Faculdade de Medicina, Universidade de Sao Paulo, Sao Paulo, Brasil; 17 Psychological Neuroscience Laboratory, Psychology Research Centre (CIPsi), School of Psychology, University of Minho, Braga, Portugal; 18 Department of Biomedical Sciences, Florida State University, Tallahassee, FL USA; 19 University of Queensland, Institute for Molecular Bioscience, St. Lucia, QLD Australia; 20 Sorbonne University, Paris Brain Institute (ICM), CNRS, INRIA, INSERM, AP-HP, Hôpital de la Pitié Salpêtrière, Paris, France; 21 Institute for Translational Psychiatry, University of Münster, Münster, Germany; 22 Bielefeld University, Medical School and University Medical Center OWL, Protestant Hospital of the Bethel Foundation, Department of Psychiatry, Bielefeld, Germany; 23 Department of Psychiatry, The University of Melbourne, Parkville, Victoria, Australia; 24 School of Psychology and Counseling, Faculty of Health, Queensland University of Technology, Kelvin Grove, QLD Australia; 25 Department of Psychology, School of Health and Psychological Sciences, City, University of London, London, UK; 26 Department of Neuroimaging, Institute of Psychiatry, Psychology and Neuroscience, King’s College London, London, UK; 27 University of East London, School of Psychology, London, UK; 28 Centre for Affective Disorders, Institute of Psychiatry, Psychology and Neuroscience, King’s College London, London, UK; 29 FIDMAG Germanes Hospitalaries Research Foundation, Barcelona, Spain; 30 CIBERSAM ISCIII, Madrid, Spain; 31 Clinical Psychopharmacology Group, Department of Psychiatry, University of Oxford, Oxford, United Kingdom; 32 SoCAT Lab, Department of Psychiatry, School of Medicine, Ege University, Izmir, Turkey; 33 Mercer University School of Medicine Department of Psychiatry and Behavioral Sciences Macon GA, Macon, USA; 34 Department of Psychology, Stanford University, Stanford, USA; 35 Department of Psychiatry and Psychotherapy, University Medicine Greifswald, Greifswald, Germany; 36 Department of Psychiatry and Mental Health, Neuroscience Institute, University of Cape Town, Cape Town, South Africa; 37 Section for Experimental Psychopathology and Neuroimaging, Department of General Psychiatry, Heidelberg University Hospital, Heidelberg, Germany; 38 Department of Psychology, Neuroscience & Behaviour, McMaster University, Hamilton, Ontario, Canada; 39 University Clinic for Radiology, University of Münster, Münster, Germany; 40 Department of Psychology, University of California, Los Angeles, Los Angeles, CA USA; 41 Department of Psychiatry and Neurosciences, Graduate School of Biomedical and Health Sciences, Hiroshima University, Hiroshima, Japan; 42 Imaging Genetics Center, Mark & Mary Stevens Institute for Neuroimaging & Informatics, Keck School of Medicine, University of Southern California, Los Angeles, CA USA; 43 Core-Facility Brainimagin, Faculty of Medicine, University of Marburg, Marburg, Germany; 44 Department of Psychology, University of Minnesota, Minneapolis, MN USA; 45 Department of Psychiatry and Psychotherapy, University Hospital Bonn, Bonn, Germany; 46 Cardiff University Brain Research Imaging Center (CUBRIC), Cardiff University, Maindy Road, Cardiff, UK; 47 Department of Psychology, University of Bath, Claverton Down, Bath, UK; 48 Department of Psychiatry and Psychotherapy, Jena University Hospital, Jena, Germany; 49 Center for Intervention and Research on adaptive and maladaptive brain Circuits underlying mental health (C-I-R-C), Halle-Jena-, Magdeburg, Germany; 50 Clinical Affective Neuroimaging Laboratory, Otto-von-Guericke-University Magdeburg, Magdeburg, Germany; 51 IWK Health, NS, Halifax, Canada; 52 Department of Psychiatry, Dalhousie University, Halifax, Canada; 53 School of Clinical Sciences, Queensland University of Technology, Brisbane, QLD Australia; 54 QIMR Berghofer Medical Research Institute, Herston, Australia; 55 Department of Psychiatry, Psychotherapy and Psychosomatics, Medical School, RWTH Aachen University, Aachen, Germany; 56 Center of Excellence on Mood Disorders, Louis A. Faillace, MD, Department of Psychiatry and Behavioral Sciences, McGovern Medical School, The University of Texas Health Science Center at Houston, Houston, USA; 57 German Center for Mental Health (DZPG), partner site Halle-Jena-, Magdeburg, Germany; 58 Origami Lab, Montreal Neurological Institute, McGill, Canada; 59 Mental Health Research Group, Institut de Recerca Sant Pau (IR Sant Pau), Barcelona, Spain; 60 Department of Psychiatry and Legal Medicine, Universitat Autònoma de Barcelona (UAB), Barcelona, Spain; 61 Department of Psychology, University of Halle, Halle, Germany; 62 German Center of Mental Health - Halle Site, Halle, Germany; 63 Amsterdam University Medical Centers, location AMC, department of Radiology and Nuclear Medicine, Amsterdam, the Netherlands; 64 Department of Psychiatry, Psychosomatic Medicine and Psychotherapy, University Hospital Frankfurt, Goethe University, Frankfurt, Germany; 65 Meditation Research Program, Department of Psychiatry, Massachusetts General Hospital, Harvard Medical School, Boston, MA USA; 66 Max Planck Institute of Psychiatry, Munich, Germany; 67 Department of Neuropsychiatry, Graduate School of Medicine, University of the Ryukyus, Okinawa, Japan; 68 West Region, Institute of Mental Health, Singapore, Singapore; 69 Yong Loo Lin School of Medicine, National University of Singapore, Singapore, Singapore; 70 Lee Kong Chian School of Medicine, Nanyang Technological University, Singapore, Singapore; 71 SAMRC Unit on Risk & Resilience, Dept of Psychiatry & Neuroscience Institute, University of Cape Town, Cape Town, South Africa; 72 University Medical Center Groningen, Cognitive Neuroscience Center, University of Groningen, Groningen, the Netherlands; 73 Department of Psychiatry, Muğla Training and Research Hospital, Muğla, Turkey; 74 Department of Psychiatry, Leiden University Medical Center, Leiden, The Netherlands; 75 Leiden Institute for Brain and Cognition, Leiden, The Netherlands; 76 Intelligent Data Analysis Laboratory, Department of Electronic Engineering, University of Valencia (UV), Valencia, Spain; 77 Institute for Community Medicine, University Medicine Greifswald, Greifswald, Germany; 78 Department of Psychiatry and Psychotherapy, University of Tuebingen, Tuebingen, Germany; 79 Department of Psychiatry and Behavioral Sciences, Division of Child and Adolescent Psychiatry, Weill Institute of Neurosciences, UCSF School of Medicine, San Francisco, USA; 80 Amsterdam UMC location Vrije Universiteit Amsterdam, Department of Psychiatry, Amsterdam, Netherlands; 81 Amsterdam Neuroscience, Brain Imaging program, Amsterdam, Netherlands

**Keywords:** Depression, Neuroscience

## Abstract

The understanding of how antidepressant (AD) use is associated with brain structure in individuals with major depressive disorder (MDD) remains incomplete. We aimed to examine the association between AD medication use and brain morphology in relation to age and sex by pooling structural neuroimaging and clinical data from 32 cohorts within the ENIGMA-MDD working group. Interaction effects of group (2076 cases with current AD use (AD), 1495 cases not currently taking AD (nAD) and 5125 healthy controls (HC)) with age and sex, and main effects of group on regional brain structure (cortical surface area and thickness, and subcortical volume) were examined. Additionally, we examined the effect of AD type (SSRI, SNRI or mirtazapine) and duration of use on brain morphology. Younger individuals in the AD group showed lower bilateral middle temporal gyrus thickness compared to nAD and HC, but this was not seen in older individuals (crossover around 50 years). Lower hippocampal volume and thinner inferior temporal gyrus were shown in AD compared to nAD. These effects were independent of group differences in disease-course-related measures, but were driven by depressive symptom severity. Greater bilateral rostral anterior cingulate thickness was found in individuals older than approximately 40 years taking mirtazapine compared to individuals taking SSRIs or SNRIs. Evidence for subtle structural brain differences in temporal and limbic regions in individuals with MDD who currently use AD medication were found compared to those not currently taking AD medication. Future longitudinal studies are needed to determine the causality of these associations.

## Introduction

Over 300 million people suffer from major depressive disorder (MDD), a leading cause of disability worldwide [1]. Antidepressant (AD) medication is the most used pharmacological therapeutic treatment for MDD [2], and the number of people who are prescribed AD continues to increase [3]. Randomized controlled clinical trials have demonstrated a modest effect of AD treatment on response and remission rates [4–6]. Despite the high rate of use, our understanding of the neurobiological mechanisms through which AD may improve mood remains limited.

Animal studies have suggested that antidepressants, in particular selective serotonin reuptake inhibitors (SSRIs), upregulate brain-derived neurotrophic factor (BDNF), enhance dendritic arborization and total dendritic length of hippocampal neurons and stimulate hippocampal proliferation and neurogenesis, thereby reversing neuronal atrophy and cell loss [7–11]. Magnetic resonance imaging (MRI) has been used to examine the association between AD medication use and brain structure in humans [12, 13]. Previous cross-sectional neuroimaging studies have shown larger hippocampal and orbitofrontal cortex (OFC) volumes in MDD patients currently taking AD (i.e. MDD patients using antidepressants at time of scanning) compared to medication-naive MDD patients, but smaller than those in healthy controls [14, 15]. This suggests a potential neuroprotective effect of AD medication on brain morphology in MDD patients. Longitudinal studies showed significant increases in hippocampal volume [16, 17], dorsolateral prefrontal cortex (DLPFC) volume [18], medial orbitofrontal cortex (mOFC) thickness [19] and thickness of other regions in prefrontal, parietal, and temporal lobes [20] in MDD patients following AD medication treatment. Given previous findings of brain structure alterations in unmedicated MDD patients [21, 22], these findings may suggest (partial) normalization of brain structure of MDD patients after AD medication treatment. Conversely, other studies found no significant longitudinal changes in hippocampal volume [23–26], amygdala volume [24], anterior cingulate cortex (ACC) volume [24] or whole-brain cortical thickness [27] in MDD patients following AD treatment.

Clinical and preclinical evidence suggests that AD medication may have different behavioral and biological effects across the lifespan [10, 28, 29], although the underlying mechanisms remain elusive. AD use might have a stronger impact on the developing brain through increased plasticity and dendritic spine density, mechanisms that have been associated with AD use [11, 30, 31]. However, our understanding of the association between AD medication use and brain structure across the lifespan in MDD patients based on neuroimaging studies remains incomplete, mainly due to heterogeneous findings across previous neuroimaging studies and limited power to detect small effects (which may result from variance in underlying mechanisms) in previous studies [32, 33].

Meta-analysis of the existing literature may increase power and address limitations related to small sample sizes, but it is hampered by heterogeneity in methods used to process neuroimaging data and limited by publication bias. The MDD Working Group within the Enhancing Neuro Imaging Genetics through Meta-Analysis (ENIGMA; http://enigma.usc.edu/) consortium aims to address these issues by performing individual participant data (IPD)-based meta-analyses or mega-analysis of pooled neuroimaging data from MDD patients across many samples, processed with harmonized protocols. In the first ENIGMA-MDD meta-analysis, we identified subcortical brain volume changes in MDD patients when compared to healthy controls [34]. In a supplementary meta-regression analysis the results revealed a trend towards lower hippocampal volume in samples with a higher percentage of patients with MDD taking AD medication [34]. A second ENIGMA-MDD meta-analysis, focusing on cortical structural abnormalities in MDD patients relative to healthy controls, revealed lower cortical thickness in adult patients, with the highest effect sizes and most widespread alterations in thickness in patients using antidepressants (effect size Cohen’s d ranging between −0.08 to −0.13 [35];). These findings seem to contradict part of the previous literature on associations between brain structure and AD use as well as evidence from animal studies which suggested normalization of brain structure with antidepressant use. However, in our prior research on the combined ENIGMA-MDD sample, the strongest subcortical and cortical brain alterations were shown in adults with MDD that were using AD medication at time of scanning compared to those who were not. Our previous ENIGMA-MDD results in adults were interpreted as a potential confounding effect of severity; i.e., patients with more severe or recurrent/chronic depression are more likely to show the strongest reduction in cortical thickness, surface area and subcortical brain volumes and are also more likely to use AD. However, this was not examined directly.

In contrast to findings in adults, an ENIGMA-MDD meta-analysis in adolescent patients showed no differences in brain measures between individuals with MDD who were on AD medication at time of scanning and healthy controls, while adolescents who were not taking AD medication at time of scanning showed lower surface area in various regions compared to healthy controls [35]. Moreover, adolescents taking antidepressants showed larger regional cortical surface area compared to adolescent cases that were not taking AD medication at time of scanning. These findings are more in line with the animal literature and potentially suggest neuroprotective effects of AD medication on surface area in adolescents.

In summary, many conflicting findings exist with regard to the association between AD medication use and brain structure alterations in MDD and it remains unclear what the influence of age and sex is on this association. To examine the association between AD use and cortical and subcortical morphology in more depth and in a larger sample with more detailed and comprehensive information (regarding type and duration) on AD use compared to the previous ENIGMA-MDD meta-analyses, we employed a large sample from the ENIGMA-MDD working group and conducted a mega-analysis to investigate the relationship between AD medication use and brain morphology in relation to age and sex. This mega-analytic approach allowed us to examine the interaction with age and sex across all cohorts and the total age range, in comparison to our previous meta-analytic approach where these interactions could only be examined within cohorts.

### Patients and methods

#### Samples

The ENIGMA-MDD Working Group is a collaboration between more than 40 international research groups, from 14 different countries worldwide, that have collected neuroimaging and clinical data from MDD patients and healthy controls. Thirty-two of these groups have collected detailed information about current AD use and agreed to participate in this study. In total, we pooled and analyzed data from 8696 individuals, including 3571 MDD patients and 5125 healthy controls (HC). MDD patients were further grouped into either: 1) cases with current AD use (AD group) (i.e., MDD patients using AD medication at the time of scanning; *n* = 2076) or 2) cases not currently taking AD (nAD group) (i.e., MDD patients not using AD medication at the time of scanning; *n* = 1495). Demographic and clinical characteristics for the AD group, nAD group and HC are presented in Table 1. A detailed summary of demographic and clinical characteristics of each sample is presented in Supplementary Table S1. Diagnostic assessment instruments and exclusion criteria for every site are listed in Supplementary Table S2. All sites obtained ethics approval from their local institutional review boards and ethics committees for the original studies and sharing of the data for this project. All participants provided informed consent at their local recruitment institution.

**Table 1:** Group differences in demographic and clinical measures between HC, the AD group and nAD group.

	HC N = 5125	AD N = 2076	nAD N = 1495	ANOVA/T-test	Chi-square	Direction of effect
Age (SD)	38.02 (15.45)	42.82 (12.78)	34.88 (15.65)	F = 133.9, p < 0.05		AD > HC>nAD
Sex (% female)	56.25	60.45	67.83		X^2^ = 65.84, p < 0.05	nAD>AD>HC
Age of onset (SD)	NA	31.97 (13.36)	25.90 (13.33)	T = 12.76, p < 0.05		AD>nAD
HDRS-17 score (SD)	NA	15.52 (7.81)	11.30 (8.56)	T = 10.11, p < 0.05		AD>nAD
BDI-II score (SD)	NA	25.81 (11.51)	16.76 (12.08)	T = 13.40, p < 0.05		AD>nAD
Number of episodes (SD)	NA	4.03 (5.61)	3.36 (6.29)	T = 2.65, p < 0.05		AD>nAD
% in remission	NA	7.94	18.00		X^2^ = 62.92; p < 0.05	nAD>AD
% recurrent MDD	NA	73.61	55.98		X^2^ = 91.42, p < 0.05	AD>nAD

NB: HC Healthy controls, AD cases with current AD use, nAD cases not currently taking AD.

### Image acquisition and processing

Structural T1-weighted images were acquired locally at each site. Image acquisition parameters and processing software of each sample are listed in Supplementary Table S3. T1-weighted images were analyzed using the fully automated and validated segmentation software FreeSurfer [36], following standardized protocols to facilitate harmonized image analysis and quality control procedures across sites (see http://enigma.ini.usc.edu/protocols/imaging-protocols/). Mean (across left and right hemisphere) cortical thickness and surface area measures of 34 cortical gray matter regions and average thickness and surface area were obtained based on the Desikan-Kiliany atlas, as well as mean volume segmentations of 7 subcortical gray matter structures together with lateral ventricles and total intracranial volume (ICV). Segmentations were visually inspected and statistically reviewed for outliers. Regions that were not properly segmented according to visual inspection, were excluded from the analyses. Despite the standardized segmentation and QC protocols for FreeSurfer, there remain site differences in the extracted brain imaging features because of the different scanner types and T1-weighted sequences, and these site differences can be a potentially strong confound in multisite analysis [37]. In order to correct the neuroimaging measures for this residual heterogeneity due to scan site, we used ComBat harmonization [37] in R (version 3.3.1) (R Core Team). ComBat adjusts for the variability between sites using an empirical Bayes approach, whereas variability associated with the included covariates (age, sex and diagnosis) is preserved. Within-site outliers (defined as measures greater than three standard deviations away from the mean of a region) were excluded from the analyses.

### Statistical framework

Group (AD group, nAD group and HC) differences in demographic and clinical characteristics were examined using analysis of variance and chi-square tests in R (version 3.3.1) [38]. To examine group differences in subcortical volumes, and cortical thickness and surface areas, multiple regression analyses were performed separately for every region of interest (ROI). The subcortical volume, cortical thickness and surface area of each ROI was introduced as the dependent variable in separate univariate models. The regression models included group (AD group, nAD group and HC), age and sex. Furthermore, in analyses with cortical surface area and subcortical volume, ICV was included as an additional covariate. Given that head size does not scale with cortical thickness, ICV was not included as a covariate in the cortical thickness analyses [39]. We first assessed the significance of group-by-age and group-by-sex interactions on regional brain structure (cortical thickness and surface area, and subcortical volume). In the case of a significant group-by-age interaction effect, the data was plotted and visual inspection was used to examine a crossover point. If no significant interaction effects were detected (false-discovery rate; FDR *p-*value > 0.05), these interaction terms were removed from the model to investigate the main effect of group, while including age and sex as covariates. If a significant group effect was present (FDR *p-*value < 0.05), we performed pairwise comparisons between each pair of groups in post-hoc tests. Effect size estimates of group-by-age and group-by-sex interactions, as well as the main effect of group were calculated using the effectsize package in R [40]. For interaction effects and the main effect of group, we calculated partial η-square as effect size. In two-group comparisons following a significant main effect of group, we calculated the Cohen’s d metric. In interaction analyses, to correct for the number of brain regions, FDR multiple comparison corrections were applied for the total number of ROIs (N = 78) including 7 subcortical volume structures, lateral ventricle volume, 34 cortical thickness regions, 34 cortical surface area regions, average thickness, and average surface area. In main effect of group analyses, to correct for the number of brain regions, FDR multiple comparison corrections were applied for the total number of ROIs showing no significant interaction effects. Lastly, in post-hoc tests, to correct for the number of brain regions, FDR multiple comparison corrections were applied for the total number of ROIs showing a significant main effect of group. Results were considered significant if the FDR corrected *p*-value was lower than 0.05.

### Secondary analyses

For ROIs that showed a significant interaction or main effect of group in the primary analyses, we performed additional sensitivity analyses to examine whether they remained significant after correcting for symptom severity or disease course. In these secondary analyses we performed the analyses described above with the following additional secondary covariates in separate analyses: total Beck Depression Inventory (BDI-II [41];) or the total 17-item Hamilton Depression Rating Scale (HDRS-17 [42];) score, number of depressive episodes (subdivided into 3 categories, either 1, 2 or 3 or higher number of depressive episodes), stage of illness (first episode vs recurrent) and remission status (acutely depressed or remitted patients). As these variables were not available in HC, we performed the secondary analyses in a sample consisting of only the AD and nAD group.

In exploratory analyses we investigated effects of duration and type of AD medication use in the AD group on all ROIs. These analyses were done in a subset of the total sample that had information on duration of use and type of AD. The analyses were performed with either 1) type of AD medication (i.e., SSRI; *n* = 405, serotonin-norepinephrine reuptake inhibitor: SNRI; *n* = 383; mirtazapine; *n* = 120) or 2) duration of current AD medication use in months (*n* = 303) included in the regression model. First, significance of interactions between either 1) type of AD medication or 2) duration of current AD medication use with age and sex was assessed. If no significant interaction effects were detected (FDR *p-*value > 0.05), these terms were removed from the model to investigate the main effect of type of AD medication or duration of current AD medication use. When a significant group-by-sex or group-by-age interaction or main effect of AD group was found for either duration or type of AD medication use, we performed the analyses using the additional secondary covariates as described above.

## Results

### Demographic and clinical characteristics

Demographic and clinical characteristics for the AD, nAD and HC group are presented in Table 1. The groups significantly differed in 1) age, with higher mean age in the AD group compared to the other groups, and higher age in HC compared to the nAD group, 2) sex, with more females in the nAD group compared to the other groups, and more females in the AD group compared to HC, 3) age of MDD onset, with an older age of onset in the AD group compared to the nAD group, 4) HDRS-17 and BDI-II scores, with higher current symptom severity in the AD group compared to the nAD group, 5) number of depressive episodes, with more depressive episodes in the AD group compared to the nAD group, 6) percentage in remission, with more patients in remission in the nAD group compared to the AD group, and 7) stage of illness with a higher ratio of recurrent episodes versus first episode in the AD group compared to the nAD group.

### Interaction and main effects on brain morphology

**Fig. 1:**
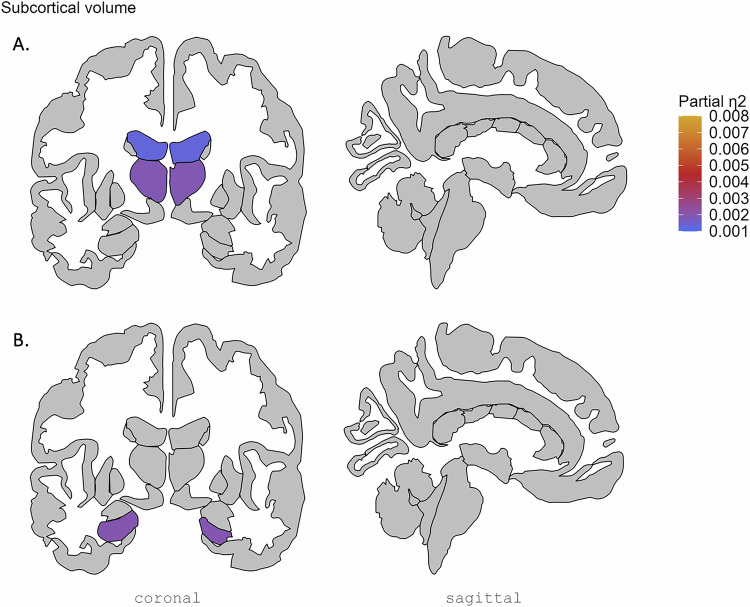
Effect sizes for subcortical volume regions showing significant group-by-age interaction effects and main effect of group. Effect sizes for volume of the (**A**) thalamus and lateral ventricles showing a significant group-by-age (AD, nAD and HC group) interaction effect and of the (**B**) hippocampus showing a significant main effect of group. AD: cases with current AD use; nAD: cases not currently taking AD; HC: healthy controls.

**Fig. 2:**
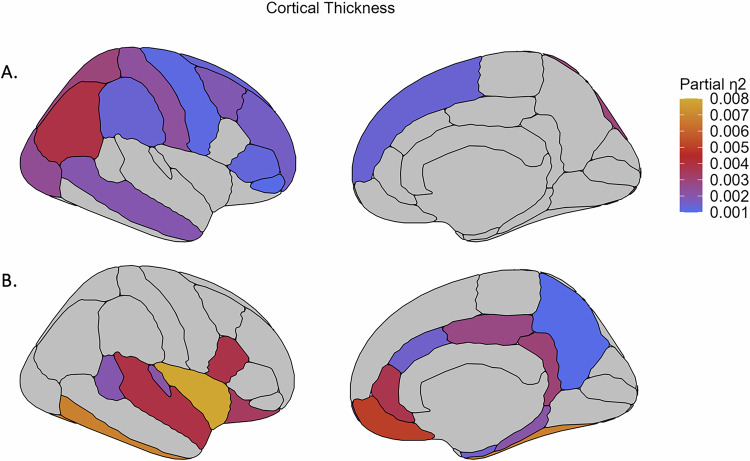
Effect sizes for cortical thickness regions showing significant group-by-age interaction effects and main effect of group. Effect sizes for (**A**) significant group-by-age (AD, nAD and HC group) interaction effects and (**B**) significant main effect of group on cortical thickness regions, plotted on the right hemisphere. AD: cases with current AD use; nAD: cases not currently taking AD; HC: healthy controls.

**Fig. 3:**
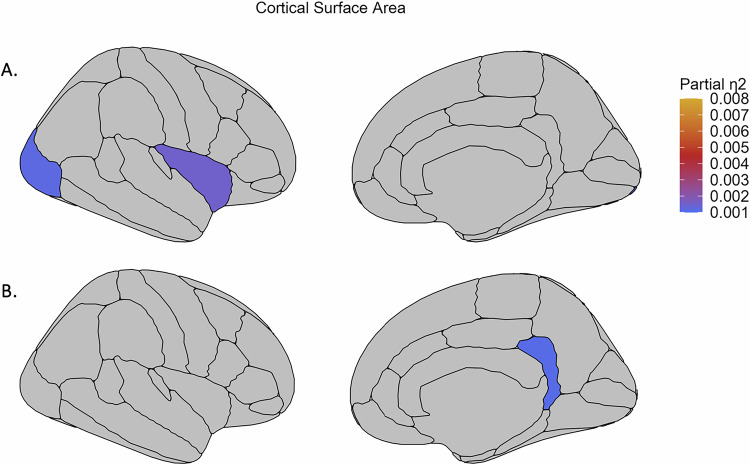
Effect sizes for cortical surface area regions showing significant group-by-age interaction effects and main effect of group. Effect sizes for surface area of the (**A**) insula and lateral occipital cortex showing a significant group-by-age (AD, nAD and HC group) interaction effect and of the (**B**) isthmus cingulate cortex showing a significant main effect of group, plotted on the right hemisphere. AD: cases with current AD use; nAD: cases not currently taking AD; HC: healthy controls.

While there were no significant group-by-sex interaction effects on brain morphology (see Supplementary Tables S4-6), significant group-by-age interaction effects were found for volume of two subcortical regions (thalamus and lateral ventricle; see Fig. 1A, Supplemental Fig. 1, Supplementary Table S4; partial η^2^ 0.0019 and 0.0013 respectively), 13 out of 35 cortical thickness regions (see Fig. 2A, Supplemental Fig. 2, Supplementary Table S5; partial η^2^ between 0.0039 and 0.0011) and surface area of the lateral occipital cortex and insula (see Fig. 3A, Supplemental Fig. 3, Supplementary Table S6; partial η^2^ 0.0012 and 0.0015 respectively). In addition, we observed significant main effects of group on the volume of the hippocampus (see Fig. 1B, Supplementary Table S4; partial η^2^ 0.0020), thickness of 17 cortical regions (see Fig. 2B, Supplementary Table S5; partial η^2^ between 0.0079 and 0.0011) and surface area of the isthmus cingulate cortex (see Fig. 3B, Supplementary Table S6; partial η^2^ 0.0011). These effects are further detailed below, reported separately for effects driven by AD use and effects driven by MDD diagnosis in general.

### AD medication use and brain morphology

**Fig. 4:**
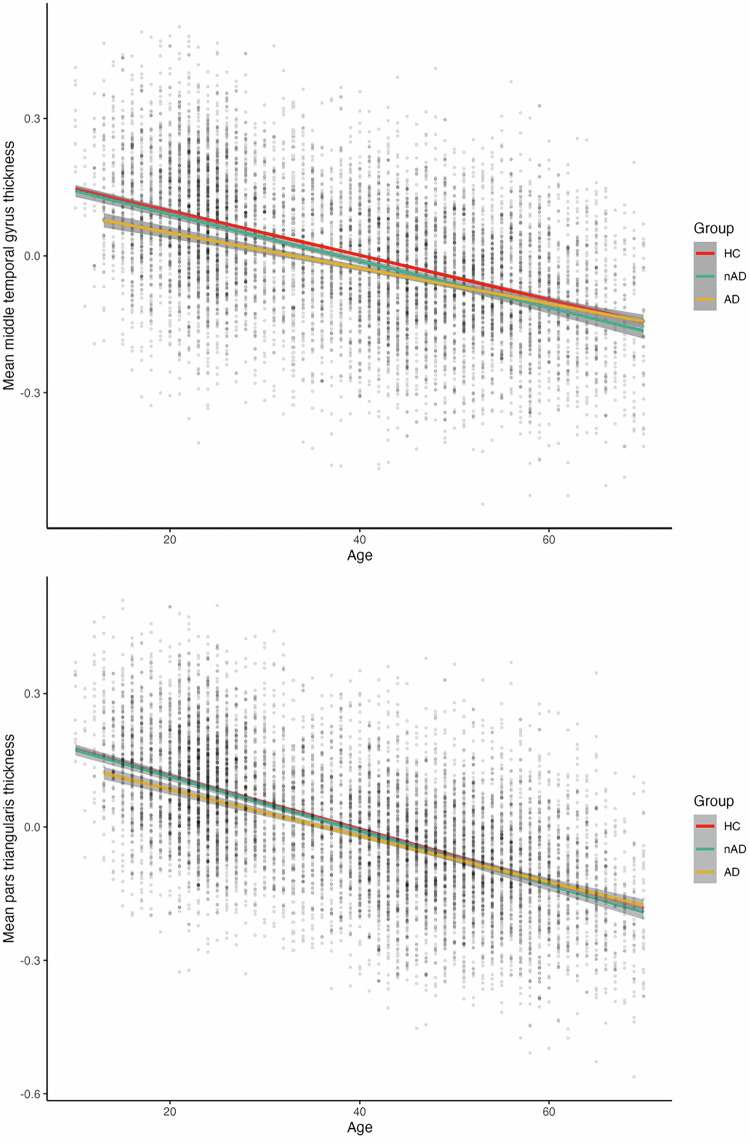
Significant group-by-age interaction effects on mean thickness of the middle temporal gyrus and inferior frontal gyrus (pars triangularis). Mean cortical thickness for these regions is presented corrected for sex (estimated marginal means). HC: Healthy controls; nAD: cases not currently taking AD; AD: cases with current AD use.

Two of the abovementioned significant group-by-age interaction effects showed that the association between age and regional cortical thickness were different in the AD group than both the nAD group and HC, suggesting an effect of current AD use, namely in the middle temporal gyrus and inferior frontal gyrus (pars triangularis). Upon visual inspection, these two regions showed a cross-over around the age of 50, with lower thickness in the AD group, compared to both the nAD group and HC in younger participants, while there was no difference in older participants (see Fig. 4). When correcting for additional clinical variables, including HDRS-17 score, number of depressive episodes, illness stage and remission status, the significant group-by-age interaction for cortical thickness of the middle temporal gyrus remained (Supplementary Table S7-S12), except for when including BDI-II score (Supplementary Table S9).

**Fig. 5:**
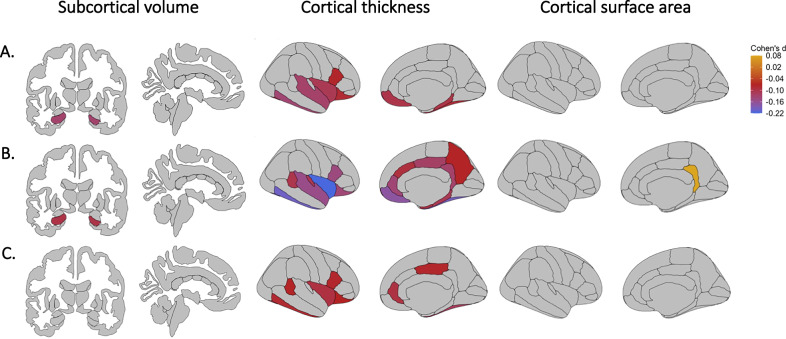
Effects sizes for subcortical volume, cortical thickness, and cortical surface area regions showing a significant difference between groups. Effects sizes for subcortical volume, cortical thickness, and cortical surface area regions showing a significant difference between (**A**) AD-nAD individuals, (**B**) AD-HC individuals, and (**C**) nAD-HC individuals. Cortical thickness and cortical surface area regions showing a significant difference between groups are plotted on the right hemisphere. AD: cases with current AD use; nAD: cases not currently taking AD; HC: healthy controls.

In terms of main effects of group, post-hoc tests showed lower hippocampal volume in the AD group compared to the nAD group (see Fig. 5A; Supplementary Table S13; Cohen’s *d* −0.1300). This finding remained significant after correcting for the number of depressive episodes, stage of illness and remission but not after correcting for current depression severity (HDRS-17 and BDI-II scores) (Supplemental Tables S14-S18). Cortical thickness of nine of the 17 significant regions showed lower cortical thickness in the AD group compared to the nAD group in the fusiform gyrus, inferior temporal gyrus, lateral orbitofrontal cortex, medial orbitofrontal cortex, parahippocampal gyrus, inferior frontal gyrus pars opercularis, superior temporal gyrus, frontal pole and insula (see Fig. 5A; Supplementary Table S19; Cohen’s *d* between −0.0765 and −0.1349). Only the inferior temporal gyrus remained significantly lower in AD compared to nAD when correcting for number of depressive episodes, stage of illness and remission status, but not when correcting for current severity (HDRS-17 and BDI-II scores) (Supplementary Tables S20–24). Differences in surface area of the isthmus cingulate cortex were not present between the AD group and nAD group (see Fig. 5A; Supplementary Table S25; Cohen’s *d* 0.0344).

### MDD diagnosis and brain morphology

For other regions that showed a significant group-by-age interaction, results showed that the association between brain structure and age differed in either or both of the AD and nAD group compared to HC (but not between the AD and nAD group), suggesting that this may be an effect of MDD diagnosis, instead of an effect of AD use. For instance, volume of the thalamus showed upon visual inspection lower volume in both the AD and nAD group before age 40 compared to HC, while no differences were observed at older age (Supplementary Figure 1). For 13 out of the 35 cortical thickness regions that showed a significant group-by-age interaction (i.e., inferior parietal cortex, supramarginal gyrus, superior parietal cortex, lateral occipital gyrus), we observed upon visual inspection lower cortical thickness in younger (mostly before age 40–50) MDD patients (both in the AD and nAD group) compared to HC (Supplementary Figure 2). There were no significant findings to suggest that the association between sex and brain structure was different in the AD and nAD group compared to HC.

Similarly, there were significant main effects of group on brain morphology which showed that brain structure was altered in specific regions in either the AD or nAD group, but not both, compared to HC (Supplementary Table S26–31). Lower hippocampal volume was observed in the AD group compared to HC (see Fig. 5B; Supplementary Table S26; Cohen’s *d* −0.0996). Lower cortical thickness was observed in 17 regions in the AD group compared to HC (see Fig. 5B; Supplementary Table S28; Cohen’s *d* between −0.08 and −0.22). Lower cortical thickness of the banks of the superior temporal sulcus, fusiform gyrus, inferior temporal gyrus, lateral orbitofrontal cortex, pars opercularis of the inferior frontal gyrus, posterior cingulate cortex, rostral anterior cingulate cortex and insula was also shown in the nAD group compared to HC (see Fig. 5C; Cohen’s *d* between −0.07 and −0.12; Supplementary Table S29). Higher surface area of the isthmus was observed in the AD group compared to HC (see Fig. 5B; Supplementary Table S30; Cohen’s *d* 0.0807).

### Type and duration of AD medication use

**Fig. 6:**
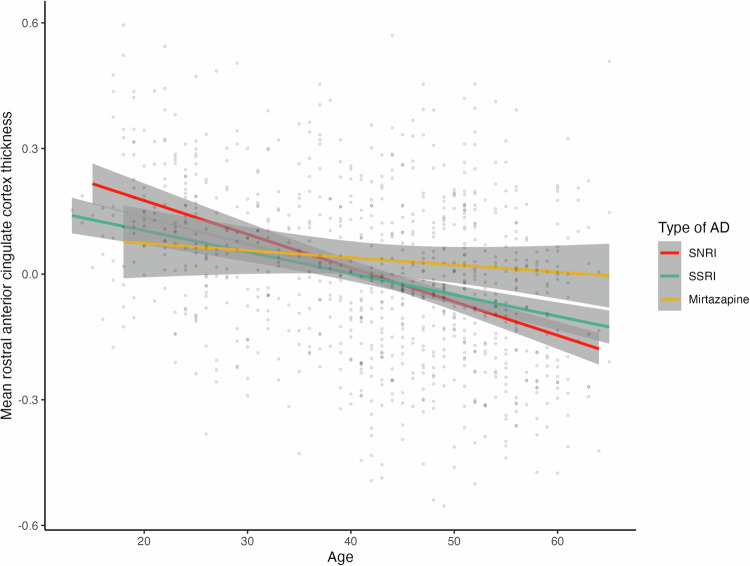
Significant age-by-type of AD medication use interaction effect for mean cortical thickness of the rostral anterior cingulate cortex. Mean cortical thickness for this region is presented corrected for sex (estimated marginal means). SNRI: Selective serotonin and noradrenalin reuptake inhibitor; SSRI: Selective serotonin reuptake inhibitor.

An age-by-type of AD medication use interaction effect was observed for thickness of the rostral anterior cingulate cortex (rACC) (Supplementary Table S33; partial η^2^ 0.0183). We observed upon visual inspection that older participants (after 40 years of age) who use mirtazapine showed higher thickness of the rACC than older participants taking SSRIs or SNRIs (see Fig. 6). After correcting for additional secondary covariates indicating severity of (the course) of depression, the interaction effect of age with type of AD medication use on thickness of the rACC remained significant, except when correcting for BDI-II severity (Supplementary Tables S38–42). In the AD group there were no significant interactions between sex and 1) type of AD medication or 2) duration of AD medication use on any brain measure (see Supplementary Tables S32–37).

For the regions that did not show significant interaction effects, we examined the main effect of type of AD medication use or duration of AD medication use. No significant findings were observed (Supplementary Tables S32–37).

## Discussion

In a large multi-study mega-analysis which included data from almost 9000 individuals, we examined whether there were interactions between age, sex and AD use group (AD, nAD and HC) on brain morphology (regional subcortical volume, and cortical thickness and surface area), while also examining main effects. In a subsample of the AD group, we examined the association between duration of AD use and brain structure, and examined interactions between brain structure and type of AD use (SSRI, SNRI, or mirtazapine). Our results reveal a complex association between AD use and MDD diagnosis on regional brain structure across the lifespan.

For two brain regions, the middle temporal gyrus and the triangular part of the inferior frontal gyrus, thickness was lower in younger participants in the AD group compared to both younger participants in the nAD group and HC (before age 50). In addition, lower hippocampal volume and inferior temporal gyrus thickness, as well as lower thickness in 8 other regions (in the frontal and temporal lobe) were shown in the AD group compared to the nAD group, suggesting an association between AD use (and not merely MDD) and brain morphology.

Our findings of lower middle and inferior temporal gyrus thickness, and hippocampal volume in the AD group compared to the nAD group could not be explained by clinical measures such as number of depressive episodes, recurrence of MDD (first episode or recurrent MDD) or remission status (current or remitted MDD), suggesting that these findings may be more specifically related to current AD use. However, these findings may have been driven by higher current depression symptom severity in the AD group compared to the nAD group, as findings were no longer significant after correcting for BDI-II and/or HDRS-17 scores. These findings may therefore, also reflect a state effect of depressed mood as opposed to, or in addition to, a direct effect of AD use. Interestingly, the differences between AD and nAD were mainly located in the temporal lobe (specifically thickness of the middle and inferior temporal gyrus and hippocampal volume). Temporal gyri, while classically thought to be involved in sensory information processing, are also important for emotional information processing and social cognition [43, 44]. In addition, the hippocampus is involved in both memory and emotional information processing [45]. Alterations of temporal lobe brain structure in individuals with MDD could therefore be related to impaired emotional and memory processing associated with MDD.

Findings from an animal study showed that antidepressant treatment was associated with reduced apoptosis in both the hippocampus and temporal cortex, suggesting that AD medication may act upon general cell survival enhancement in these regions [46]. Also, previous studies using positron emission tomography (PET) and single photon emission computed tomography (SPECT) suggest that AD medication may normalize fronto-temporal metabolism in MDD patients [12]. Moreover, prior studies in adult individuals with MDD showed that short-term use of SSRIs (e.g., paroxetine and citalopram) was associated with increased middle temporal gyrus and hippocampal volume [47]. Thus, AD medication use seems to predominantly impact the temporal cortex, which could potentially be explained by its role in emotional information processing, which shows a strong connection to MDD. However, while some studies across all age ranges also report increases in brain morphology following AD treatment, others report a decrease or find no differences (for a review please see [48]).

Specific genetic phenotypes of some individuals with MDD might have partly driven our findings of lower middle and inferior temporal gyrus thickness, and hippocampal volume in the AD group compared to the nAD group. A previous study investigating the association between the serotonin transporter promoter polymorphism (5-HTTLPR) and functional responses to citalopram in healthy controls, showed a greater decrease in glucose metabolism in temporal and frontal lobes in response to citalopram in specific 5-HTTLPR genotypes compared to other 5-HTTLPR genotypes [49]. Therefore, the 5-HTTLPR may be associated with reduced capacity for neuroplasticity in amongst others, the temporal lobe [50]. Exploring the effect of AD treatment on brain structure in individuals with MDD with various 5-HTTLPR genotypes might be interesting for future studies.

Results of interaction analyses with age showed altered regional brain morphology in younger MDD patients (i.e., before 40–50 years of age), for example lower thalamus volume, and lower thickness in frontal, occipital and parietal lobe regions, compared to HC, in both the AD and nAD groups. As this association was observed in both the AD and nAD groups, these effects seem driven by the diagnostic status of MDD rather than AD medication use per se. In older participants we did not observe a difference in brain morphology between these groups. We speculate that due to developmental neural changes (including structural, neurochemical and molecular changes) the brain of younger people might be more vulnerable to the effects of chronic stress associated with MDD and its underlying pathophysiology (e.g., inflammation, increased oxidative stress, increased cortisol) [51, 52]. Alternatively, physiological aging effects in older participants might obscure MDD-related changes in brain structure in this age group [53]. These findings extend our previous findings showing MDD associations with structural brain alterations that are modulated by age [35]. In previous meta-analyses we were not able to examine this question properly, as diagnosis-by-age interactions were done within each site separately (and thus limited to the age range of each specific sample) and then meta-analyzed.

Cortical thickness of multiple regions was lower in the AD group compared to HC, while these differences were not present when comparing AD to nAD. This may suggest a subtle AD effect, with cortical thickness of the nAD group being in between the AD and HC groups. This included cortical thickness of 9 regions, mainly located in the frontal, cingulate and temporal cortex. These regions are involved in integrating cognitive, emotional and social information [54–58] Evidence suggests that early changes in emotional information processing underlie subsequent mood improvement following AD medication treatment [59–61]. Thus, alterations of frontal, cingulate and temporal cortex brain structure in individuals with MDD could be related to changes in emotional information processing and mood improvement following AD medication treatment.

Our finding of altered brain structure in younger patients with MDD who are also using AD medication adds to the ongoing discussions about the implications of AD use in children, adolescents and young people [62, 63]. However, given the fact that the ENIGMA-MDD cohorts did not collect information on duration of *lifetime* AD use or other psychotropic medication and given the cross-sectional nature of this study, we cannot interpret these findings as direct effects of AD use in younger people.

Supplemental analyses in a subgroup of the AD group for whom this information was available revealed that there was a significant interaction between type of AD (SSRI, SNRI or mirtazapine) and age on brain structure. These results showed that in older people (after approximately 40 years), those using mirtazapine showed greater thickness of the rACC than older MDD patients using SSRIs or SNRIs. Similar to SNRIs, mirtazapine acts on both the serotonin and noradrenergic neurotransmitter systems. Mirtazapine blocks pre-synaptic noradrenergic ɑ2 receptors, and thereby increases norepinephrine release, while also blocking heteroreceptors on serotonergic neurons and thereby increasing serotonin release [64]. In contrast to SNRIs such as venlafaxine, mirtazapine does not influence monoamine reuptake [64]. Mirtazapine may specifically influence the ACC, as it has a high binding potential in cortical regions [65], and the ACC is enriched with serotonin receptors [66]. Given that the rACC plays a crucial role in emotional regulation and reward processing [67–69], the observed greater rACC thickness in older MDD patients using mirtazapine may reflect a compensatory neuroplastic response. This could be driven by mirtazapine-induced serotonergic modulation, potentially enhancing synaptic plasticity and structural integrity in a region critical for emotional control and reward processing. Additionally, a previous randomized controlled trial has shown that a 4-week treatment with mirtazapine boosts serum levels of brain-derived neurotrophic factor (BDNF) while BNDF declined in patients treated with venlafaxine [70]. Given the important role of BDNF in neurogenesis and neuroplasticity, we speculate that mirtazapine, through this mechanism, may exert a protective effect against age-related decline in thickness of the rACC. However, we note that mirtazapine is not a first-line treatment for MDD and is often prescribed after insufficient response to more standard medication such as SSRIs or SNRIs. Hence, an alternative explanation may be that mirtazapine associated differences in brain morphology could be confounded by a longer and potentially more complex history of AD use. While in this study we did not observe associations between duration of current AD use and brain morphology, conclusions from this are limited in the view of a lack of information on lifetime duration of AD use. The observed association between mirtazapine use and greater rACC thickness in older MDD patients might partly reflect clinical characteristics of MDD patients for whom mirtazapine is prescribed, such as insomnia [71, 72], rather than a direct pharmacological effect of AD medication use itself.

While the clinical relevance of subtle effects remains to be fully elucidated, they may still reflect meaningful insights into the neural mechanisms underlying AD medication use in MDD, particularly in light of the large sample size and use of harmonized protocols to obtain brain morphology measures. However, the findings need to be interpreted in light of several limitations. First, this is a cross-sectional study, and future large and ideally randomized longitudinal studies are needed to precisely examine the interaction between age and AD use on brain structure. Importantly, such future longitudinal studies should not only examine the short-term but also long-term (over many years) effects of AD use on brain structure, while adequately correcting for potential confounders. Since depressive symptom severity influenced observed effects of AD use on temporal lobe structures, future longitudinal studies are needed to examine the long-term effects of AD use on brain structure while accounting for depression severity across different ages and at various time points before and following AD initiation. This approach could provide a more comprehensive understanding of the interplay between AD treatment, brain structure alterations, and depressive symptoms over time. Additionally, future longitudinal studies should investigate both short-term and long-term effects on AD use on brain structure in MDD patients, as well as the trajectories of brain changes in medication-naïve MDD patients and healthy individuals. The findings from the current study may help select brain regions of interest for future longitudinal studies on this topic. Following MDD patients who discontinue AD treatment during such a longitudinal study would be particularly valuable for assessing long-term effects of AD use. Second, while we have detailed clinical information available on MDD in this sample, we cannot rule out the presence of (pre-clinical) neurodegenerative diseases in older participants, which may have affected our results. Third, we were unable to take other factors into account which may play a role in changes in brain structure with age, and may differ between groups, including IQ, education level, lifestyle and metabolic factors [73–75]. Finally, we could not control for other psychotropic medication that participants may have been using or presence of comorbid anxiety disorders, as this information was not available for most participants.

To conclude, we report the first robust evidence for an association between current AD medication use and regional brain morphology in a mega-analysis of almost 9000 participants. Specifically, we observed lower middle temporal gyrus thickness in AD users (under ~50 years of age), and lower hippocampal volume and inferior temporal gyrus thickness in the AD group compared to the nAD group. In addition, we observed several brain alterations, which were likely driven by MDD diagnosis, and not AD use per se. Supplementary findings suggest mirtazapine may have a differential effect on brain structure in older participants compared SSRIs or SNRIs, however future research needs to replicate this finding and explore potential underlying mechanisms. In addition, it is recommended for future studies to take age into account when examining the association between AD medication use and the brain.

## Supplementary information

Supplementary figures

Supplementary tables

## Data Availability

The datasets generated during and/or analysed during the current study are not publicly available due to privacy restrictions.
