# Supplementary material for: Regional brain morphology and current antidepressant use: findings from 32 international cohorts from the ENIGMA major depressive disorder working group
Source: Mol Psychiatry. 2025 Nov 3;30(12):5625–36. doi: 10.1038/s41380-025-03310-8 (PMC12602329; doi:10.1038/s41380-025-03310-8)

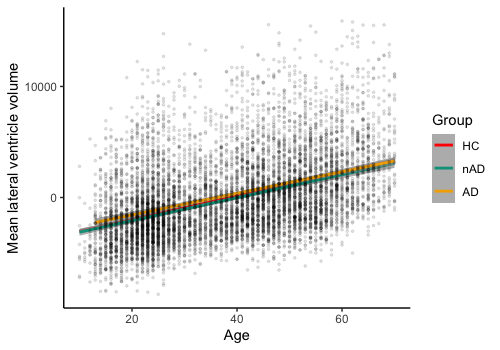

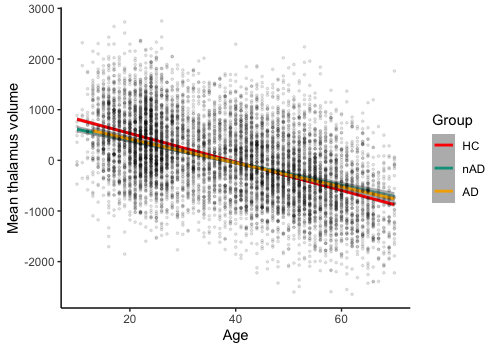


**Supplemental Figure 1.**  Significant age-by-group interaction effects for subcortical volume regions (Thalamus, Lateral Ventricle). Mean subcortical volume for these regions is presented corrected for sex (estimated marginal means). HC: Healthy controls; nAD: cases not currently taking AD; AD: cases with current AD use


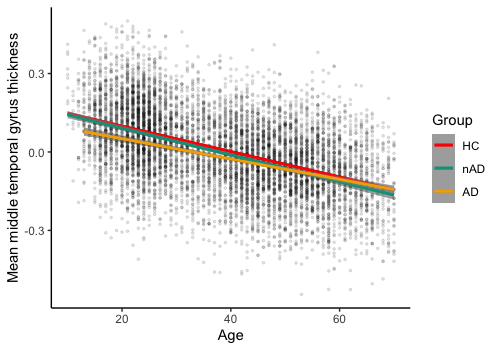

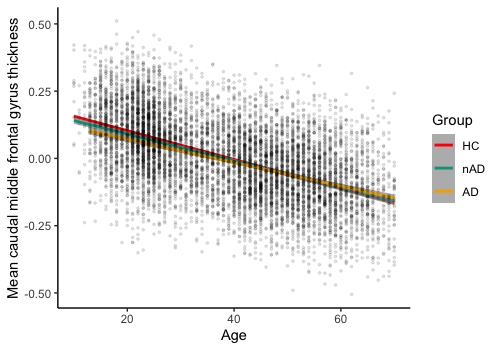

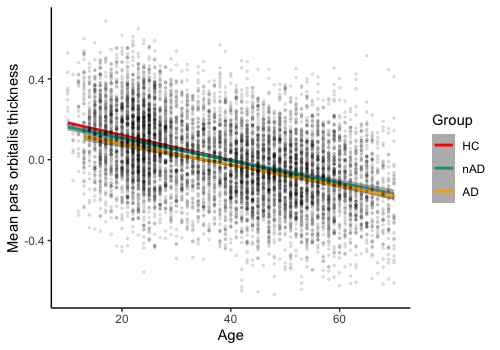

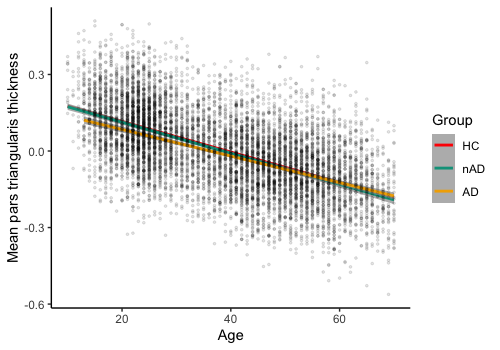

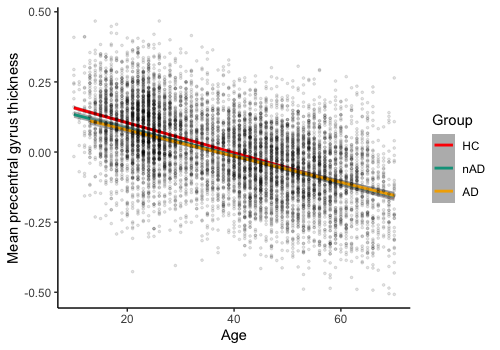

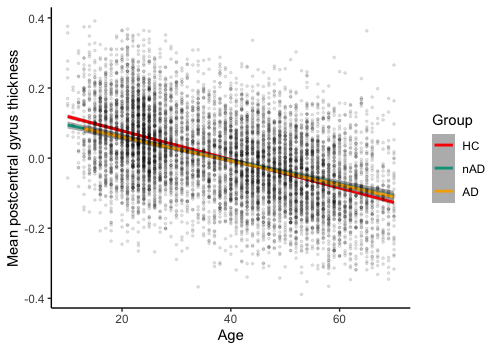

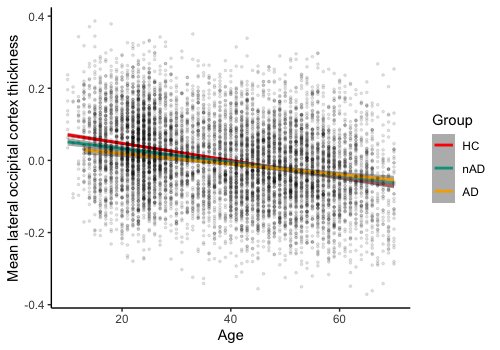

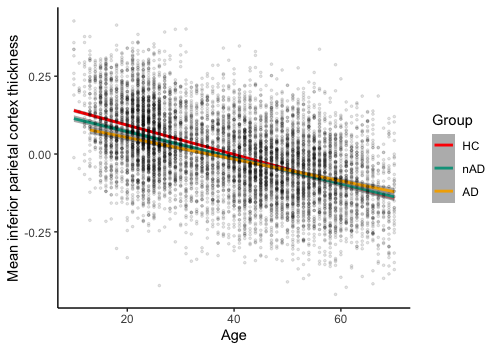


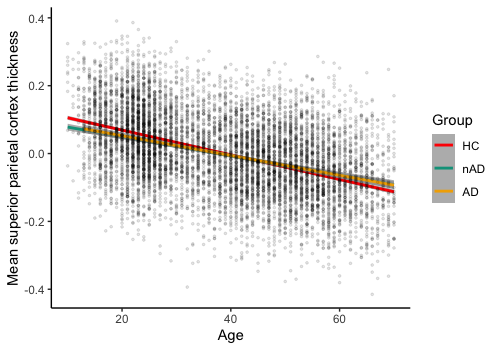

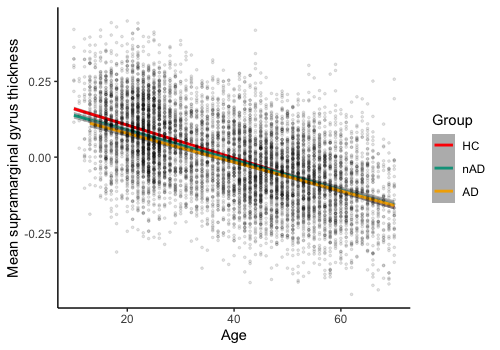

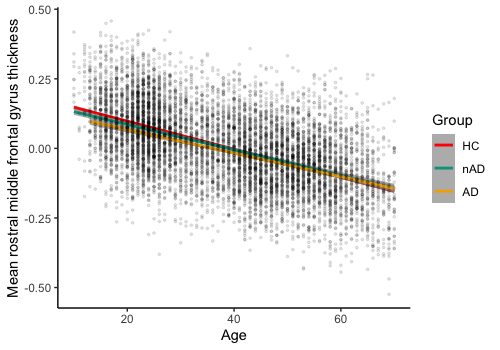

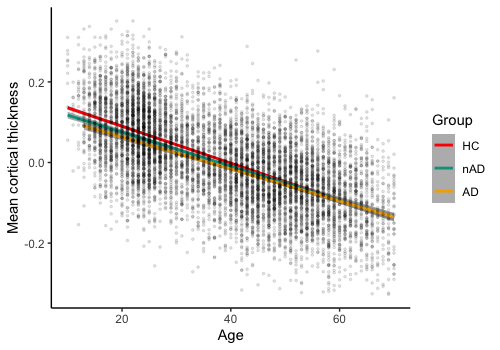

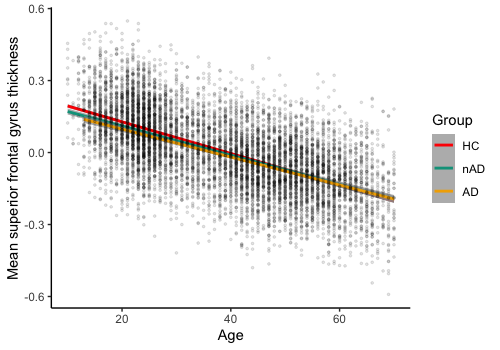


**Supplemental Figure 2.** Significant age-by-group interaction effects for cortical thickness regions. Mean cortical thickness for these regions is presented corrected for sex (estimated marginal means). HC: Healthy controls; nAD: cases not currently taking AD; AD: cases with current AD

**Supplemental Figure 3**. Significant age-by-group interaction effects for cortical surface area of the lateral occipital cortex and insula. Mean cortical surface area of these regions is presented corrected for sex (estimated marginal means). HC: Healthy controls; nAD: cases not currently taking AD; AD: cases with current AD use
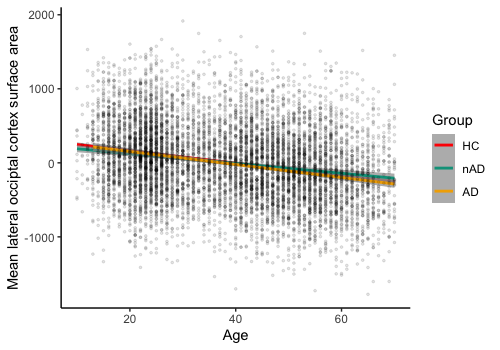

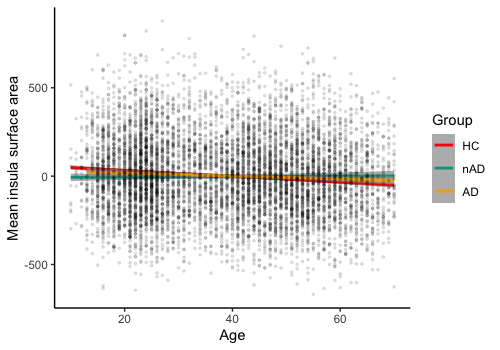

Supplement: Supplementary file 1 — Supplementary figures [file 41380_2025_3310_MOESM1_ESM.docx]
