# Supplementary material for: Regional brain morphology and current antidepressant use: findings from 32 international cohorts from the ENIGMA major depressive disorder working group
Source: Mol Psychiatry. 2025 Nov 3;30(12):5625–36. doi: 10.1038/s41380-025-03310-8 (PMC12602329; doi:10.1038/s41380-025-03310-8)
Supplement: Supplementary file 2 — Supplementary tables [file 41380_2025_3310_MOESM2_ESM.docx]

Supplementary Tables

**Content:**

**Supplementary Table S1. ENIGMA – Major Depressive Disorder Working Group demographic and clinical characteristics of MDD patients.** Age, sex, depression symptom severity, mean number of depressive episodes, percentage of sample with recurrent episode MDD patients and percentage of sample with remitted MDD patients, shown per participating site and per group.

**Supplementary Table S2**. **ENIGMA – Major Depressive Disorder Working Group Instrument for diagnosing Major Depressive Disorder and exclusion criteria per participating site.**

**Supplementary Table S3**. **ENIGMA – Major Depressive Disorder Working Group MRI image acquisition and processing per participating site.**

**Supplementary Table S4. Full mega-analytic results for mean subcortical volume regions for the 1) Group*Age interaction, 2) Group*Sex interaction and 3) main effect of Group controlling for age, sex and ICV.**

**Supplementary Table S5. Full mega-analytic results for mean cortical thickness regions for the 1) Group*Age interaction, 2) Group*Sex interaction and 3) main effect of Group controlling for age and sex.**

**Supplementary Table S6. Full mega-analytic results for mean cortical surface area regions for the 1) Group*Age interaction, 2) Group*Sex interaction and 3) main effect of Group controlling for age, sex and ICV.**

**Supplementary Table S7. Secondary analyses for mean cortical thickness regions: Group*Age interaction within the sample of patients with MDD controlling for age and sex.**

**Supplementary Table S8. Secondary analyses for mean cortical thickness of the middle temporal gyrus: Group*Age interaction within the sample of patients with MDD controlling for age, sex and HDRS-17 score.**

**Supplementary Table S9.** **Secondary analyses for mean cortical thickness of the middle temporal gyrus: Group*Age interaction within the sample of patients with MDD controlling for age, sex and BDI-II score.**

**Supplementary Table S10.** **Secondary analyses for mean cortical thickness of the middle temporal gyrus: Group*Age interaction within the sample of patients with MDD controlling for age, sex and number of depressive episodes.**

**Supplementary Table S11.** **Secondary analyses for mean cortical thickness of the middle temporal gyrus: Group*Age interaction within the sample of patients with MDD controlling for age, sex and stage of illness**.

**Supplementary Table S12.** **Secondary analyses for mean cortical thickness of the middle temporal gyrus: Group*Age interaction within the sample of patients with MDD controlling for age, sex and remission status.**

**Supplementary Table S13.** **Mega-analytic results for mean subcortical volume of the hippocampus for the AD versus nAD group comparison controlling for age, sex and ICV.**

**Supplementary Table S14. Secondary analyses for mean subcortical volume of the hippocampus: AD versus nAD group comparison controlling for age, sex, ICV and HDRS-17 score.**

**Supplementary Table S15. Secondary analyses for mean subcortical volume of the hippocampus: AD versus nAD group comparison controlling for age, sex, ICV and BDI-II score.**

**Supplementary Table S16.** **Secondary analyses for mean subcortical volume of the hippocampus: AD versus nAD group comparison controlling for age, sex, ICV and number of depressive episodes.**

**Supplementary Table S17. Secondary analyses for mean subcortical volume of the hippocampus: AD versus nAD group comparison controlling for age, sex, ICV and stage of illness.**

**Supplementary Table S18. Secondary analyses for mean subcortical volume of the hippocampus: AD versus nAD group comparison controlling for age, sex, ICV and remission status.**

**Supplementary Table S19. Mega-analytic results for mean cortical thickness regions for the AD versus nAD group comparison controlling for age and sex.**

**Supplementary Table S20. Secondary analyses for mean cortical thickness regions: AD versus nAD group comparison controlling for age, sex and HDRS-17 score.**

**Supplementary Table S21. Secondary analyses for mean cortical thickness regions: AD versus nAD group comparison controlling for age, sex and BDI-II score.**

**Supplementary Table S22.** **Secondary analyses for mean cortical thickness regions: AD versus nAD group comparison controlling for age, sex and number of depressive episodes.**

**Supplementary Table S23. Secondary analyses for mean cortical thickness regions: AD versus nAD group comparison controlling for age, sex and stage of illness.**

**Supplementary Table S24. Secondary analyses for mean cortical thickness regions: AD versus nAD group comparison controlling for age, sex and remission status.**

**Supplementary Table S25. Mega-analytic results for mean cortical surface area of the isthmus cingulate for the AD versus nAD group comparison controlling for age, sex and ICV.**

**Supplementary Table S26. Mega-analytic results for mean subcortical volume of the hippocampus for the AD versus HC group comparison controlling for age, sex and ICV.**

**Supplementary Table S27. Mega-analytic results for mean subcortical volume of the hippocampus for the nAD versus HC group comparison controlling for age, sex and ICV.**

**Supplementary Table S28. Mega-analytic results for mean cortical thickness regions for the AD versus HC group comparison controlling for age and sex.**

**Supplementary Table S29. Mega-analytic results for mean cortical thickness regions for the nAD versus HC group comparison controlling for age and sex.**

**Supplementary Table S30. Mega-analytic results for mean cortical surface area of the isthmus cingulate for the AD versus HC group comparison controlling for age, sex and ICV.**

**Supplementary Table S31. Mega-analytic results for mean cortical surface area of the isthmus cingulate for the nAD versus HC group comparison controlling for age, sex and ICV.**

**Supplementary Table S32.** **Secondary analyses for mean subcortical volume regions: 1) AD type*Age interaction, 2) AD type*Sex interaction and 3) main effect of AD type within the sample of the AD group controlling for age, sex and ICV.**

**Supplementary Table S33. Secondary analyses for mean cortical thickness regions: 1) AD type*Age interaction, 2) AD type*Sex interaction and 3) main effect of AD type within the sample of the AD group controlling for age and sex.**

**Supplementary Table S34.** **Secondary analyses for mean cortical surface area regions: 1) AD type*Age interaction, 2) AD type*Sex interaction and 3) main effect of AD type within the sample of the AD group controlling for age, sex and ICV.**

**Supplementary Table S35.** **Secondary analyses for mean subcortical volume regions: 1) AD duration*Age interaction, 2) AD duration*Sex interaction and 3) main effect of AD duration within the sample of the AD group controlling for age, sex and ICV.**

**Supplementary Table S36.** **Secondary analyses for mean cortical thickness regions: 1) AD duration*Age interaction, 2) AD duration*Sex interaction and 3) main effect of AD duration within the sample of the AD group controlling for age and sex.**

**Supplementary Table S37. Secondary analyses for mean cortical surface area regions: 1) AD duration*Age interaction, 2) AD duration*Sex interaction and 3) main effect of AD duration within the sample of the AD group controlling for age, sex and ICV.**

**Supplementary Table S38. Secondary analyses for mean cortical thickness of the rostral anterior cingulate cortex: AD type*Age interaction within the sample of the AD group controlling for age, sex and HDRS-17 score.**

**Supplementary Table S39. Secondary analyses for mean cortical thickness of the rostral anterior cingulate cortex: AD type*Age interaction within the sample of the AD group controlling for age, sex and BDI-II score.**

**Supplementary Table S40.**  **Secondary analyses for mean cortical thickness of the rostral anterior cingulate cortex: AD type*Age interaction within the sample of the AD group controlling for age, sex and number of depressive episodes.**

**Supplementary Table S41. Secondary analyses for mean cortical thickness of the rostral anterior cingulate cortex: AD type*Age interaction within the sample of the AD group controlling for age, sex and stage of illness.**

**Supplementary Table S42. Secondary analyses for mean cortical thickness of the rostral anterior cingulate cortex: AD type*Age interaction within the sample of the AD group controlling for age, sex and remission status.**

**Table S1.** **ENIGMA – Major Depressive Disorder Working Group demographic and clinical characteristics of MDD patients.**

| **Site** | **Group** | **N** | **Age (SD)** | **%**  **Female** | **HDRS-17**  **(SD)** | **BDI-II**  **(SD)** | **Number episodes (SD)** | **%**  **Recurrent** | **%**  **Remitted** |
| --- | --- | --- | --- | --- | --- | --- | --- | --- | --- |
| AFFDIS | HC | 20 | 31.75(12.10) | 50.0 | - | - | - | - | - |
|  | AD | 28 | 39.04(15.29) | 46.4 | 23.6(9.9) | 29.6(11.6) | 6.1(11.2) | 92.9 | 0.0 |
|  | nAD | - | - | - | - | - | - | - | - |
| AMC | HC | - | - | - | - | - | - | - | - |
|  | AD | - | - | - | - | - | - | - | - |
|  | nAD | 50 | 29.36(4.73) | 100.0 | - | - | 3.1(4.2) | 44.0 | 26.0 |
| Barcelona | HC | 32 | 46.03(8.13) | 71.9 | - | - | - | - | - |
|  | AD | 58 | 46.97(7.99) | 81.0 | 13.7(8.1) | - | 4.0(4.8) | 67.2 | 37.9 |
|  | nAD | - | - | - | - | - | - | - | - |
| Bidirect | HC | 435 | 52.17(8.12) | 50.1 | - | - | - | - | - |
|  | AD | 502 | 48.65(7.36) | 59.8 | 13.8(6.7) | - | 4.7(6.5) | - | 0.0 |
|  | nAD | 72 | 50.57(6.76) | 62.5 | 12.1(7.1) | - | 4.8(8.0) | - | 0.0 |
| CLING | HC | 321 | 25.12(5.17) | 59.5 | - | - | - | - | - |
|  | AD | 43 | 36.05(11.90) | 51.2 | 20.1(4.5) | - | 3.6(3.6) | 51.2 | 4.65 |
|  | nAD | - | - | - | - | - | - | - | - |
| Calgary | HC | 23 | 17.26(2.60) | 60.9 | - |  | - | - | - |
|  | AD | 17 | 17.35(2.23) | 47.1 | 20.6(7.8) | 29.5(10.3) | 1.0(0) | 94.1 | 0.0 |
|  | nAD | 24 | 16.46(1.44) | 70.8 | 21.1(6.2) | 23.3(13.9) | 1.0(0) | 29.2 | 0.0 |
| Cardiff | HC | - | - | - | - | - | - | - | - |
|  | AD | 39 | 45.85(11.17) | 66.7 | 19.5(4.7) | 35.9(9.4) | - | 87.2 | 0.0 |
|  | nAD | - | - | - | - | - | - | - | - |
| EPISCA | HC | 30 | 14.73(1.55) | 86.7 | - |  | - | - | - |
|  | AD | - | - | - | - | - | - | - | - |
|  | nAD | 18 | 15.39(1.54) | 83.3 | - | - | - | 0.0 | 0.0 |
| FIDMAG | HC | 34 | 45.94(11.67) | 64.7 |  | - | - | - | - |
|  | AD | 31 | 49.19(12.34) | 64.5 | 25.3(4.3) | - | 3.4(5.3) | 61.3 | 0.0 |
|  | nAD | - | - | - | - | - | - | - | - |
| FOR2107 | HC | 636 | 32.47(12.33) | 63.1 | - | - | - | - | - |
|  | AD | 304 | 38.05(13.58) | 63.5 | 10.3(6.6) | - | 4.7(5.8) | 69.1 | 14.5 |
|  | nAD | 193 | 35.92(13.53) | 64.8 | 6.1(6.2) | - | 3.5(6.7) | 48.2 | 46.6 |
| Groningen | HC | 23 | 42.78(14.37) | 73.9 | - | - | - | - | - |
|  | AD | 10 | 45.10(12.89) | 50.0 | - | 20.2(8.3) | - | 50.0 | 0.0 |
|  | nAD | 12 | 41.50(14.86) | 91.7 | - | 25.9(9.0) | - | 66.7 | 0.0 |
| Hiroshima | HC | 169 | 39.88(12.39) | 61.5 | - | - | - | - | - |
|  | AD | 134 | 43.75(10.89) | 49.3 | 18.7(5.5) | 30.2(9.3) | 1.8(1.2) | 49.3 | 0.0 |
|  | nAD | 9 | 39.56(11.40) | 22.2 | 18.8(7.6) | 27.7(11.8) | 3.1(4.2) | 55.6 | 0.0 |
| Houston | HC | 101 | 38.80(12.27) | 67.3 | - | - | - | - | - |
|  | AD | - | - | - | - | - | - | - | - |
|  | nAD | 77 | 39.19(13.32) | 70.1 | 10.2(8.0) | 16.7(15.2) | 5.1(8.0) | 57.1 | 48.1 |
| Houston adolescent | HC | 72 | 13.60(2.21) | 40.3 | - | - | - | - | - |
|  | AD | - | - | - | - | - | - | - | - |
|  | nAD | 24 | 13.58(2.19) | 37.5 | 10.8(6.7) | - | 1.9(2.0) | 25.0 | - |
| London | HC | 61 | 51.72(7.94) | 52.5 | - | - | - | - | - |
|  | AD | 50 | 48.70(8.53) | 66.0 | - | 15.6(12.4) | 5.2(3.8) | 100.0 | - |
|  | nAD | 19 | 45.63(9.73) | 73.7 | - | 14.4(8.3) | 5.7(3.9) | 100.0 | - |
| MPIP | HC | 203 | 48.55(12.30) | 59.1 | - | - | - | - | - |
|  | AD | 278 | 47.69(12.61) | 55.8 | 24.7(6.8) | - | 2.2(3.8) | 78.8 | 14.0 |
|  | nAD | 52 | 42.73(13.91) | 59.6 | 26.2(6.2) | - | 0.3(0.8) | 42.3 | 11.5 |
| Magdeburg | HC | 39 | 33.10(8.36) | 33.3 | - | - | - | - | - |
|  | AD | 32 | 40.75(13.29) | 46.9 | 15.4(6.4) | - | 2.7(1.5) | 50.0 | 0.0 |
|  | nAD | 7 | 30.14(11.92) | 42.9 | 13.6(7.5) | - | - | - | 0.0 |
| McMaster | HC | 48 | 29.96(11.43) | 62.5 | - | - | - | - | - |
|  | AD | 29 | 41.41(12.25) | 58.6 | 10.3(6.7) | - | 5.9(7.2) | - | 0.0 |
|  | nAD | 22 | 26.09(9.35) | 45.4 | 14.1(8.4) | - | 2.1(2.2) | - | 0.0 |
| Melbourne | HC | 102 | 19.58(2.98) | 52.9 | - | - | - | - | - |
|  | AD | 22 | 18.73(2.16) | 54.5 | - | - | 3.7(6.7) | 54.5 | 0.0 |
|  | nAD | 101 | 19.52(2.89) | 51.5 | - | - | 2.6(2.3) | 65.3 | 0.0 |
| Minnesota | HC | 40 | 15.68(2.00) | 65.0 | - | - | - | - | - |
|  | AD | 16 | 15.63(1.20) | 81.3 | - | 21.6(11.4) | - | 31.3 | 18.8 |
|  | nAD | 52 | 15.33(1.98) | 75.0 | - | 27.2(12.1) | - | 32.7 | 5.8 |
| Moral dilemma | HC | 46 | 18.50(1.77) | 100.0 | - | - | - | - | - |
|  | AD | - | - | - | - | - | - | - | - |
|  | nAD | 24 | 19.42(2.19) | 100.0 | - | - | 1.0(0) | 66.7 | 0.0 |
| Muenster | HC | 723 | 35.36(12.14) | 57.1 | - | - | - | - | - |
|  | AD | 231 | 37.53(11.92) | 58.4 | 18.9(4.4) | 26.2(10.3) | 4.2(5.0) | 77.9 | 8.23 |
|  | nAD | 27 | 39.63(14.12) | 63.0 | 18.3(3.7) | 24.2(9.6) | 4.8(5.7) | 74.1 | 14.8 |
| NESDA | HC | 65 | 40.29(9.74) | 64.6 | - | - | - | - | - |
|  | AD | 56 | 38.30(10.44) | 64.3 | - | - | 5.4(8.3) | 53.6 | 0.0 |
|  | nAD | 98 | 36.55(10.51) | 68.4 | - | - | 6.7(11.8) | 58.2 | 0.0 |
| Oxford | HC | - | - | - | - | - | - | - | - |
|  | AD | - | - | - | - | - | - | - | - |
|  | nAD | 29 | 15.83(1.28) | 75.9 | - | - | 1.1(0.3) | 10.3 | 0.0 |
| QTIM | HC | 297 | 21.99(3.35) | 65.0 | - | - | - | - | - |
|  | AD | 30 | 22.17(2.91) | 70.0 | - | - | 1.3(0.9) | - | - |
|  | nAD | 73 | 22.01(3.26) | 76.7 | - | - | 1.2(0.7) | - | - |
| SHIP | HC | 382 | 52.54(10.84) | 45.5 | - | - | - | - | - |
|  | AD | 22 | 52.73(7.71) | 63.6 | - | 19.8(11.9) | - | 63.6 | - |
|  | nAD | 103 | 51.47(10.20) | 74.8 | - | 10.3(9.2) | - | 39.8 | - |
| SHIP trend | HC | 851 | 48.49(12.90) | 44.8 | - | - | - | - | - |
|  | AD | 52 | 49.12(10.07) | 73.1 | - | 17.7(10.6) | - | 78.8 | - |
|  | nAD | 245 | 47.69(11.22) | 62.9 | - | 11.4(7.0) | - | 60.0 | - |
| UCSF | HC | 90 | 15.29(1.29) | 47.8 | - | - | - | - | - |
|  | AD | - | - | - | - | - | - | - | - |
|  | nAD | 75 | 15.63(1.34) | 65.3 | - | 26.6(11.8) | 1.7(1.1) | 45.3 | 9.3 |
| Sao Paulo | HC | 88 | 30.19(8.15) | 45.5 | - | - | - | - | - |
|  | AD | 13 | 29.38(7.17) | 61.5 | 15.9(8.1) | - | 2.4(1.3) | 46.2 | 0.0 |
|  | nAD | 11 | 28.45(9.90) | 81.8 | 14.6(12.1) | - | 1.8(0.7) | 45.4 | 0.0 |
| Singapore | HC | 17 | 38.53(4.64) | 52.9 | - | - | - | - | - |
|  | AD | 18 | 41.06(7.11) | 50.0 | 6.3(5.5) | - | - | 61.1 | - |
|  | nAD | - | - | - | - | - | - | - | - |
| SOCAT | HC | 100 | 36.42(13.64) | 90.0 | - | - | - | - | - |
|  | AD | 38 | 41.42(13.52) | 89.5 | 12.5(8.1) | - | 2.4(1.3) | 71.0 | 52.6 |
|  | nAD | 41 | 38.02(12.22) | 90.2 | 14.1(7.1) | - | 2.4(1.1) | 80.4 | 31.7 |
| Stanford | HC | 77 | 35.16(10.83) | 70.1 | - | - | - | - | - |
|  | AD | 23 | 37.70(8.75) | 65.2 | 16.3(5.8) | 26.0(8.5) | 6.4(4.5) | 100.0 | 0.0 |
|  | nAD | 37 | 37.05(10.14) | 67.6 | 15.6(6.1) | 27.4(10.7) | 3.0(1.6) | 86.5 | 0.0 |

**Age, sex, depression symptom severity, mean number of depressive episodes, percentage of sample with recurrent episode MDD patients and percentage of sample with remitted MDD patients, shown per participating site and per group.**

HC: Healthy controls; AD: cases with current AD use; nAD: cases not currently taking AD; BDI: Beck Depression Inventory; HDRS: Hamilton Depression Rating Scale

**Table S2**. **ENIGMA – Major Depressive Disorder Working Group Instrument for diagnosing Major Depressive Disorder and exclusion criteria per participating site.**

| **Site** | **Instrument for diagnosing MDD** | **Exclusion criteria** |
| --- | --- | --- |
| AFFDIS | ICD-10 interview | All subjects exclusion criteria: current or history of neurological disorder or brain injury. current substance abuse or dependence (not including nicotine). pregnancy. MRI contraindications. inability to give consent. MDD specific: comorbid psychiatric diagnosis. Healthy control specific: current or history of psychiatric diagnosis. |
| AMC | M.I.N.I. Plus | Less than three week medication-free interval before scanning. current psychotropic medication use. a history of chronic or neurological disorder. family history of sudden heart failure or epileptic attacks. pregnancy (tested via urine sampling prior to the assessment). breast feeding. alcohol dependence and contra-indications for an MRI scan (e.g.. ferromagnetic fragments). Participants agreed to abstain from smoking. caffeine and alcohol use for 24 hours prior to the assessments. |
| Barcelona | DSM-IV-TR according to CIDI-interview and HAMD | The exclusion criteria for healthy participants were: lifetime psychiatric diagnoses. first-degree relatives with psychiatric diagnoses and clinically significant physical or neurological illnesses. Axis I comorbidity according to DSM-IV-TR criteria was an exclusion criteria for all participants. |
| Bidirect | M.I.N.I. Neuropsychiatric Interview. IDS. HAMD. CESD. ICD-10 | Dementia. addiction. |
| CLING | ICD-10 interview | MDD subjects: past or actual presence of other axis I diagnoses other than anxiety disorders. alcohol/cannabis abuse and tobacco dependence; neurological or other medical conditions that could be related to affective symptoms. Control subjects: no medical history. including neurological and psychiatric history. as well as no previous or actual use of psychotropic medication |
| Calgary | KSADS | Dalhousie Sample: A history of neurological illness. medical illness. claustrophobia. >21 year of age. or the presence of a ferrous implant or pacemaker. University of Calgary: Left handed; history of seizures. epilepsy or other neurological or psychiatric diagnoses (specifically bipolar disorder. psychosis. pervasive developmental disorder. eating disorders. PTSD); pregnancy. |
| Cardiff | Hamilton Depression Rating Scale  (HDRS-17) | Psychotic symptoms. current substance dependence. eating disorders. claustrophobia and other MRI contraindications. and ongoing non-pharmacological treatment. |
| EPISCA | ADIS | Primary DSM-IV clinical diagnosis of ADHD. ODD. CD. pervasive developmental disorders. post-traumatic stress disorder. Tourette's syndrome. obsessive–compulsive disorder. bipolar disorder. and psychotic disorders; current substance abuse; history of neurological disorders or severe head injury; age < 12 or > 21 years; pregnancy; left-handedness; IQ score < 80 as measured by the Wechsler Intelligence Scale for Children (WISC) (Wechsler. 1991) or Adults (Wechsler. 1997); and general MRI contra- indications. |
| FIDMAG | DSM-IV-TR criteria | Patients were excluded (i) if they were left-handed; (ii) if they were younger than 18 or older than 65 years; (iii) if they had a history of brain trauma or neurological disease; (iv) if they had shown alcohol/ substance abuse within 12 months prior to participation; and (v) if they had undergone electroconvulsive therapy in the previous 12 months. |
| FOR2107 | SCID-1 | Exclusion criteria all: any MRI contraindications; any neurological abnormalities. Exclusion criteria controls: any current or former psychiatric disorder; Exclusion criteria patients: substance dependence or current benzodiazepine treatment (wash out of at least three half-lives before study participation). |
| Groningen | M.I.N.I.-SCAN | Exclusion MDD: Comorbid axis-I disorders other than anxiety disorders or past substance abuse. other psychotropic medication than stable use of SSRI/SNRI/TCA. established cardiovascular disease. active and concrete suicidal plans. iAD-freeequate language proficiency. cognitive impairments or neurological disease that interferes with task performance. Exclusion CTL: Same as MDD. lifetime history of MDD. BDI>8. |
| Hiroshima | M.I.N.I. | MDD patients: comorbid psychiatric disorders other than MDD. Control subjects: any history of psychiatric disorder. |
| Houston | SCID interview | MDD subjects: age below 18; lifetime or current diagnosis of psychotic disorder. or bipolar I or II disorder; substance abuse/dependence in 6 months prior to study inclusion; current major medical problems. Control subjects: age below 18; current major medical problems; current psychiatric or neurologic disorder; history of psychiatric disorders in a first-degree relative; current major medical problems. Both groups: MRI contra-indications. |
| Houston adolescent | Major depressive disorder (MDD) diagnosis according to DSM-IV | MDD subjects: head trauma with residual effects. neurological disorders. uncontrolled major medical conditions based on patient self reports and current drug abuse. In addition. Healthy controls (HC) were excluded if they had a history of any Axis I disorder or had a first-degree relative with any Axis I disorder. |
| London | SCAN interview | Contraindications to MRI. diagnosis of neurological disorder. head injury leading to loss of consciousness or conditions known to affect brain structure or function (including alcohol or substance misuse). if they or a first-degree relative had ever fulfilled criteria for mania. hypomania. schizophrenia or mood-incongruent psychosis. |
| MPIP | M-CIDI/SCAN interview | Munich Antidepressant Response Signature (MARS) study MDD subjects (clinical consensus diagnosis or M-CIDI (since 2008)): depressive syndromes secondary to any medical or neurological condition (e. g.. intoxication. drug abuse. stroke). the presence of manic. hypomanic or mixed affective symptoms. lifetime diagnosis of alcohol dependence. illicit drug abuse or the presence of severe medical conditions (e.g.. ischemic heart disease). Patients with bipolar depression were excluded for the current MR study. Control subjects: age > 65. MMSE<27. presence of severe somatic diseases or lifetime history of the following axis I disorders as assessed by the M-CIDI interview: alcohol dependence. drug abuse or dependence. possible psychotic disorder. mood disorder. anxiety disorder including OCD and PTSD. somatoform disorder. dissociative disorder NOS. and eating disorder 2. Recurrent unipolar depression (RUD) study: MDD subjects (SCAN interview): presence of manic episodes. mood incongruent psychotic symptoms. the presence of a lifetime diagnosis of intravenous drug abuse and depressive symptoms only secondary to alcohol or substance abuse or to medical illness or medication.Control subjects: presence of severe somatic diseases or life-time history of anxiety and affective disorders according to the Composite International Diagnostic-Screener (CIDI-S). All subjects: gross incidental MR findings such as territorial infarction. tumor. hydrocephalus. malformations and anatomical deviations (e.g. enlarged ventricles) that prevent appropriate image processing were additional exclusion criteria. 3. MR images of 9 additional controls acquired at the LMU. Munich. meeting equivalent criteria as the RUD control sample were included. |
| Magde burg | ICD-10 interview. HAMD | MDD subjects: history of seizures. medication with glutamate modulating drugs (ketamine. riluzole. etc.) or benzodiazepines. prior electroconvulsive therapy (ECT) treatments and pregnancy. atypical forms of depression. any additional psychiatric disorder. and a history of substance abuse or dependence. Control subjects: psychiatric illness. Both groups: contraindications against MRI. major medical and neurological illness. |
| McMaster | SCID interview | Comorbid Axis 1 disorders excluded. including for example. psychosis. bipolar. PTSD substance dependence or current active eating disorder. Exclusion criteria included: i) treatment with anti-cholinergic or typical (first generation) anti-psychotic medication; ii) electroconvulsive therapy (ECT) or transcranial magnetic stimulation (TMS) within the past year; iii) a history of substance dependence or significant and recent (< 1 year) substance abuse; iv) a history (within the past 12 months) of an endocrine or other medical disorder known to adversely affect cognition (e.g.. Cushing’s. uncontrolled diabetes. seizure disorder); and v) English comprehension lower than a grade 6 reading level. |
| Melbourne | SCID interview | MDD subjects: lifetime or current SCID-I diagnosis of psychotic disorder. or bipolar I or II disorder. Control subjects: any SCID-I diagnosis or medication use. Both groups: Acute or unstable medical disorder; general MRI contraindications. |
| Minnesota | Schedule for Affective Disorders and Schizophrenia for School-Age Children–Present and Lifetime Version and the Children’s Depression Rating Scale–Revised (CDRS-R). | Exclusion criteria for both groups included the presence of a neurologic or other chronic medical condition. mental retardation. pervasive developmental disorder. substance use disorder. bipolar disorder. or schizophrenia. |
| Moral dilemma | SCID interview | MDD subjects: lifetime or current SCID-I diagnosis of psychotic disorder. or bipolar I or II disorder; current antidepressant medication use. Control subjects: any SCID-I diagnosis or medication use. Both groups: Acute or unstable medical disorder; general MRI contraindications. |
| Muenster | SCID interview | MDD subjects: presence of bipolar disorder. schizoaffective disorders and schizophrenia; substancerelated disorders or current benzodiazepine treatment (wash out of at least three half-lives before study participation). and former electroconvulsive therapy. Control subjects: any current or former psychiatric disorder. Both groups: any neurological abnormalities. MRI contra-indications. |
| NESDA | CIDI interview | MDD subjects: presence of axis-I disorders other than MDD. panic disorder. social anxiety disorder. or generalized anxiety disorder and any use of psychotropic medication other than stable use of SSRIs or infrequent benzodiazepine use (i.e.. equivalent to 2 doses of 10 mg of oxazepam 3 times per week or use within 48 hours prior to scanning). Control subjects: no Axis-I diagnosis. no medication use. All subjects: presence or history of major internal or neurological disorder. dependence on or recent abuse (past year) of alcohol and/or drugs. hypertension. and general MRI contraindications. |
| Oxford | SCID interview | MDD: psychosis or substance dependence (DSM-IV). clinically significant risk of suicidal behaviour. having contraindications to escitalopram treatment or being treated with psychotropic medication less than three weeks before the study (five weeks in the case of fluoxetine); HC: current or past history of Axis I disorder as defined by DSM-IV; Both groups: major somatic or neurological disorders. pregnancy or breast-feeding. contra-indications to MR imaging or concurrent medication which could alter emotional processing. |
| QTIM | CIDI interview | MDD subjects: presence of axis-I disorders other than MDD and anxiety disorders Control subjects: antidepressant use. psychiatric disorders All subjects: relatedness between subjects. left handedness. history of neurological or other severe medical illness. head injury or current or past diagnosis of substance abuse. use of cognition affecting medication and general MRI contraindications. |
| SHIP- START | M-CIDI interview | MDD subjects: presence of axis-I disorders other than MDD. anxiety disorders. conversion. somatization and eating disorder. Control subjects: no lifetime diagnosis of depression. no antidepressiva. and severity index=0 All subjects: We removed subjects with medical conditions (e.g. a history of cerebral tumor. stroke. Parkinson’s diseases. multiple sclerosis. epilepsy. hydrocephalus. enlarged ventricles. pathological lesions) or due to technical reasons (e.g. severe movement artifacts or inhomogeneity of the magnetic field). |
| SHIP- TREND | M-CIDI interview | MDD subjects: no special exclusion criteria Control subjects: no lifetime diagnosis of depression. no antidepressiva. and severity index=0 All subjects: We removed subjects with due to medical conditions (e.g. a history of cerebral tumor. stroke. Parkinson’s diseases. multiple sclerosis. epilepsy. hydrocephalus. enlarged ventricles. pathological lesions) or due to technical reasons (e.g. severe movement artifacts or inhomogeneity of the magnetic field). |
| UCSF | KSADS (semi-structured interview based on DSM) for MDD. DISC/DPS for HCL | Exclusion criteria for all participants included: 1) use of pharmacotherapeutics for treating psychiatric conditions within the past 6 months. 2) misuse of drugs within two months prior to MRI scanning; 3) two or more alcoholic drinks per week within the previous month (as assessed by the Customary Drinking and Drug Use Record; CDDR) (Brown et al. 1998); 4) a full scale IQ score of less than 75 (as assessed by the Wechsler Abbreviated Scale of Intelligence; WASI) (Wechsler. 1999); 5) contraindications for MRI including ferromagnetic implants and claustrophobia; 6) pregnancy or the possibility of pregnancy; 7) left-handedness; 8) prepubertal status (as assessed as Tanner stages of 1 or 2) (Tanner. 1962); 9) inability to understand and comply with procedures; 10) neurological disorder (including meningitis. migraine. or HIV); 11) head trauma; 12) learning disability; 13) serious health problems; and 14) complicated or premature birth (i.e.. birth before 33 weeks of gestation). The MDD group was subject to the additional exclusion criterion of a primary psychiatric diagnosis other than MDD. The HCL group was subject to the additional exclusion criteria of: 1) history of mood or psychotic disorders in a first- or second-degree relative (as assessed by the Family Interview for Genetics; FIGS) (Maxwell. 1992); and 2) current or lifetime DSM-IV-TR Axis I psychiatric disorder. |
| Sao Paulo | SCID interview | People with psychotic disorders due to a general medical condition or substance-induced psychosis were excluded. Additional exclusion criteria were: (a) history of head injury; (b) presence of neurological disorders or any organic disorders that could affect the central nervous system; and (c) contraindications for MRI. Exclusion cri- teria specific for the control group were personal history of psychosis or other Axis I disorders. except substance misuse or mild anxiety disorders. |
| Singapore | SCID interview | Exclusion criteria 1) History of significant head injury 2)Neurological diseases such as epilepsy. cerebrovascular accident 3) Impaired thyroid function 4) Steroid use 5) DSM IV alcohol or substance use or dependence 6) Contraindications to MRI (e.g. pacemaker. orbital foreign body. recent surgery/procedure with metallic devices/implants deployed) using standard MRI Request Form from NNI 7)Pregnant women 8) Claustrophobia. |
| SOCAT | SCID interview | Exclusion criteria 1) History of significant head injury 2) Neurological diseases such as epilepsy. cerebrovascular accident 3) Other diagnoses on Axis I disorders4). |
| Stanford | SCID interview | MDD subjects: presence of axis-I disorders other than MDD. anxiety and eating disorders. Control subjects: control individuals did not meet diagnostic criteria for any current psychiatric. Both groups: alcohol / substance abuse or dependence within six months prior to MRI scanning. history of head trauma with loss of consciousness > 5 min. aneurysm. or any neurological or metabolic disorders that require ongoing medication or that may affect the central nervous system (including thyroid disease. diabetes. epilepsy or other seizures. or multiple sclerosis). MRI contraindications. or bad MRI data (e.g.. extreme movement). |

ICD: International Statistical Classification of Diseases and Related Health Problems; M.I.N.I.: Mini-International Neuropsychiatric Interview; CIDI: Composite International Diagnostic *Interview*; HAMD: Hamilton Rating Scale for Depression; IDS: Inventory of Depressive Symptomatology; CESD: Center for Epidemiological Studies *Depression;* KSADS; The Kiddie Schedule for Affective Disorders and Schizophrenia; ADIS: The Anxiety and Related Disorders *Interview* Schedule; SCID: Structured Clinical *Interview* for DSM Disorders; SCAN: Schedules for Clinical Assessment in Neuropsychiatry; DISC: The diagnostic *Interview* Schedule for Children; DPS: Depressive Psychopathology Scale

**Table S3**. **ENIGMA – Major Depressive Disorder Working Group MRI image acquisition and processing per participating site.**

| **Site** | **Scanner vendor and type** | **Acquisition parameters** | **FreeSurfer version** | **Slice orientation** | **Operating system** |
| --- | --- | --- | --- | --- | --- |
| AFFDIS | 3T Siemens Magnetom Trio Tim | 3D T1 (176 slices; TR = 2250 ms; TE = 3.26 ms; FOV 256; voxel size 1X1X1mm) | 5.3 | Sagittal | Linux CentOS |
| AMC | 3T Philips | T1 sequence details: 3D-TFE sequence TR= 9.7 ms. TE=4.6ms. matrix 192x192. voxel size = 0.875 x 0.875 x 1.2 mm; 120 slices. Axial plane. Philips 3T Ingenia 16 channel coil | 5.3 | Transverse | freesurfer-Linux-centos6_x86_64-stable-pub-v5.3.0 |
| Barcelona | 3T Philips Achieva | 3D MPRAGE images (Whole-brain T1-weighted); TR=6.7ms. TE=3.2ms; 170 slices. voxel size 0.89X0.89X1.2 mm. Image dimensions 288X288X170; field of view: 256X256X204; slice thickness: 1.2 mm; with a sagittal slice orientation. T1 contrast enhancement. flip angle: 8º. grey matter as a reference tissue. ACQ matrix MXP = 256X240 and turbo-field echo shots (TFE) = 218. | 6 | Sagittal | Scientific Linux 5 |
| Bidirect | 3 T Philips Intera scanner | 3D T1-weighted turbo field echo images were collected with the following parameters: TR = 7.26. TE = 3.56. 9° flip angle. 160 sagittal slices. matrix dimension 256 x 256. FOV = 256 x 256mm. 2mm slice thickness (reconstructed to 1mm) and a resulting voxel size of 1x1x1mm. | 5.3 | Sagittal | freesurfer-Linux-centos6_x86_64-dev-20161222-499fc91 |
| CLING | 3T Siemens Tim Trio | Standard 3D T1-weighted turbo fast low angle shot (turbo FLASH); voxel size 1 mm x 1 mm x 1mm (based on the ADNI protocol (Jack et al. 2008); TR=225 msec; TE=3.26 msec. FOV=256 x 256 x 192 | 5.3 | Sagittal | Linux |
| Calgary | 1.5T Siemens Magnetom Vision. 3T GE Discovery MR750 | 1.5T: A sagittal scout series was acquired to test image quality. 3D fast low angle shot (FLASH) sequence was used to acquire data from 124 1.5 mm-thick contiguous coronal slices through the entire brain (echo time = 5ms. repetition time = 25ms. acquisition matrix = 256 x 256 pixels. field of view = 24 cm and flip angle = 40°). 3T: Anatomical imaging acquisition parameters: axial acquisition. repetition time (TR). 2200 milliseconds (ms); echo time (TE). 3.04 ms; TI. 766. 780; flip angle. 13 degrees; 208 partitions; 256 × 256 matrix; and field of view. 256. | 5.3 | Dalhousie sample. coronal; Calgary sample. axial | MacOs Sierra |
| Cardiff | 3T whole body MRI system (General Electric. Milwaukee. USA) with an 8-channel head coil | High-resolution anatomical scan (Fast Spoiled Gradient-Recalled-Echo [FSPGR] sequence): 178 slices. TE=3 ms. TR=7.9 ms. voxel size=1.0×1.0×1.0 mm3. FA=15°. FOV=256×256. | 5.3 | Sagittal | freesurfer-Linux-centos6_x86_64-stable-pub-v5.3.0 |
| EPISCA | 3T Philips Achieva | a sagittal 3-dimensional gradient-echo T1-weighted image was acquired (repetition time = 9.8 ms; echo time = 4.6 ms; flip angle = 8°; 140 sagittal slices; no slice gap; field of view =256 × 256 mm; 1.17 × 1.17 × 1.2 mm voxels; duration = 4:56 min) | 5.3 | Sagittal | Ubuntu 14.04.5 LTS (Linux 3.13.0-153-generic x86_64) |
| FIDMAG | 1.5T. GE Signa | 3D T1: matrix size = 512 × 512. 180 contiguous axial slices. voxel resolution = 0.47 × 0.47 × 1mm. no slice gap. TE = 3.93ms. TR = 2000ms and inversion time (TI) = 710ms. flip angle = 15 degrees | 6 | Axial | Linux-centos6_x86_64 |
| FOR2107 | 3T Siemens Magnetom TiroTim syngo MR B17 | Sequence: 3D T1-weighted magnetization prepared rapid acquisition gradient echo (MPRAGE) - Sagittal Acquisition Direction. # of Slices 176. 0.5mm Slice Gap. 1.0x1.0x1.0 Voxel Size (mm3). TI 900 ms. TE 2.26 ms. TR 1900 ms. Flip Angle 9. | 5.3 | Sagittal | Red Hat Enterprise Linux Server release 5.11 (Tikanga) |
| Groningen | 3T Philips | 3D T1-weighted scan (170 slices; TR = 9ms; TE = 3.6ms; 256x231 matrix of 1×1×1 mm voxels) | 5.3 |  |  |
| Hiroshima | 3T Siemens (Spectra. Verio.Dot). 3T GE (Signa HDxt) | T1 256x256x256 matrix of 1x1x1mm voxels (Siemens: ADNI MPRAGE (tfl). GRAPPA. 192 slices. GE: SPGR. 184 slices) *Detailed scanning parameter sheets are available for all 4 scanners on request | 5.3 | Sagittal | Linux_Ubuntu_18.04 |
| Houston | subjects in 20000s: 1.5 T Philips Medical Systems Gyroscan Intera; subjects in 30000s: 3T Siemens Allegra | Subjects in the 20000s: Fast field echo sequence- repetition time (TR) = 24 ms. echo time (TE) = 4.99 ms. flip angle = 40°. slice thickness = 1 mm. matrix size = 256 × 256 and 150 slices. Subjects in 30000s: MPRAGE- repetition time (TR) = 1750 ms. echo time (TE) = 4.39 ms. flip angle = 8°. slice thickness = 1 mm. matrix size = 208 × 256 and 160 slices. | 5.3 | Subjects in 20000s: Sagittal; Subjects in 30000s: Transverse | Fedora 19 |
| Houston adolescent | subjects in 20000s: 1.5 T Philips Medical Systems Gyroscan Intera; subjects in 30000s: 3T Siemens Allegra | Subjects in the 20000s: Fast field echo sequence- repetition time (TR) = 24 ms. echo time (TE) = 4.99 ms. flip angle = 40°. slice thickness = 1 mm. matrix size = 256 × 256 and 150 slices. Subjects in 30000s: MPRAGE- repetition time (TR) = 1750 ms. echo time (TE) = 4.39 ms. flip angle = 8°. slice thickness = 1 mm. matrix size = 208 × 256 and 160 slices. | 5.3 | Subjects in 20000s: Sagittal; Subjects in 30000s: Transverse | Fedora 19 |
| London | 1.5T GE Signa HDx | ADNI-1 MPRAGE pulse sequence (details at http://adni.loni.ucla.edu/research/protocols/mri-protocols/). | 5.3 | Sagittal | Linux-centos4_x86_64 |
| MPIP | 1.5T GE and Siemens (the latter: only few cases) | #1: T1-weighted SPGR sagittal 3D volume. TR=1030 msec; TE=3.4 msec; 124 slices; matrix=256x256; FOV=23.0x23.0 cm2; voxel size=0.8975 mm x0.8975 mm x 1.2- 1.4 mm; flip angle=90°; birdcage resonator. #2: same scanner as #1. platform update Signa Excite. sagittal T1-weighted (spin echo sequence. TR=9.7 msec. TE=2.1 msec; FOV=25.0x25.0 cm2. voxel size=0.875 mm x0.875 mm x1.2 mm. 124- 132 slices. flip angle=90°. #3: Siemens 1.5 Tesla. Vario. 3D MPRAGE. TR=11.6 msec; TE=4.9 msec; FOV 23x23 cm2; matrix 512x512; 126 axial slices; voxel site 0.45 mm x 0.45 mm x 1.5 mm. (only N=2 subjects) | 5.3 | 1.5 GE: sagittal. 1.5 Siemens: axial | Linux 2.6.37.1-1.2- desktop x86_64 |
| Magde burg | 7 Tesla. MAGNETOM Terra MRI scanner from Siemens with a 32-channel phased-array head coil.  3 Tesla Siemens MAGNETOM Trio scanner (Siemens. Erlangen. Germany) | 3D-MPRAGE sequence (TE = 2.73 ms. TR = 2300 ms. T1 = 1050 ms. flip angle = 7°. bandwidth = 140 Hz/pixel. acquisition matrix = 320 × 320 x 224. isometric voxel size = 0.8 mm3.  High resolution T1 -weighted structural MRI scans of the brain were acquired for structural reference using a 3D -MPRAGE sequence (TE = 4.77 ms. TR = 2500 ms. T1 = 1100 ms. flip angle = 7°. bandwidth = 140 Hz/pixel. acquisition matrix = 256 × 256 × 192. isometric voxel size = 1.0 mm3). | 5.3 | Sagittal | Oracle Linux Server_x86_64 |
| McMaster | 1.5T (GE); 3T(GE) | 1.5-T. Sigma GE Genesis-based Echo-Speed scanner running version 5.7 software and using a standard 30-cm circularly polarized head coil. Sagittal anatomic images were acquired by using a 3D/FSPGR/20 sequence (flip angle=20; echo delay time in-phase (TE). minimum repetition time (TR)=300 ms; inversion recovery=300 ms; matrix=512x256; field of view (FOV)=24 cm; scan thickness=1.2 mm). 3-T MRI Sigma GE Genesis (General Electric Medical Systems. Milwaukee. WI). Sagittal T-1 weighted images were acquired using a 3D FSPGR-IR sequence. (TR/TE=10.3/2.1 ms; flip angle=20; inversion time=300; matrix=512x256; FOV=24; and slice thickness=1.2 mm. | 5 |  |  |
| Melbourne | 3T GE Signa Excite | 3D BRAVO sequence 140; TR=7900 ms; TE=3000 ms; flip angle=13º; FOV=256 mm; matrix=256 x 256 | 5.3 | Axial | Linux Debian x86 64 |
| Minnesota | 3.0 Tesla Tim Trio scanner; Siemens Corp | A 5-minute structural scan was acquired using a T1-weighted. high-resolution. magnetization-prepared gradient-echo sequence: repetition time. 2530 milliseconds; echo time. 3.65 milliseconds; inversion time. 1100 milliseconds; flip angle. 7°; field of view. 256 × 176 mm; voxel size. 1-mm isotropic; 224 slices; and generalized. autocalibrating. partially parallel acquisition acceleration factor. 2. | 5.3 | Coronal | Linux |
| Moral dilemma | 3T GE Signa Excite | 3D BRAVO sequence: 140 contiguous slices; repetition time. 7900 ms; echo time. 3000 ms; flip angle. 13°; in a 25.6-cm field of view. with a 256 × 256 pixel matrix and a slice thickness of 1 mm (1 mm gap). | 5.3 | Axial | Linux Debian x86 64 |
| Muenster | 3T Philips Gyroscan Intera | 3D fast gradient echo sequence (turbo field echo). repetition time = 7.4 milliseconds. echo time = 3.4 milliseconds. flip angle = 9°. two signal averages. inversion prepulse every 814.5 milliseconds. acquired over a field of view of 256 (feet -head [FH]) × 204 (anterior -posterior [AP]) × 160 (right -left [RL]) mm. phase encoding in AP and RL direction. reconstructed to cubic voxels of .5 mm × .5 mm × .5 mm | 5.3 | Sagittal | Red Hat Enterprise Linux Server release 5.11 (Tikanga) |
| NESDA | 3T Phillips Achieva/Intera | 3D gradient-echo T1-weighted sequence. TR=9 msec; TE=3.5 msec; flip angle 8º. FOV = 256 mm; matrix: 25x62x56; in plane voxel size = 1 mm × 1 mm x 1 mm; 170 slices. | 5 | Sagittal | SHARK HPC. Linux environment |
| Oxford | 3T Siemens Tim Trio | Voxel resolution 0.78 x 0.8 x 0.78 mm on a 208 x 256 x 200 grid. TE/TI/TR= 4.8/1100/2040 ms | 5.3 |  |  |
| QTIM | Bruker 4T Wholebody MRI | 3D T1 weighted sequence. TR=1500 msec; TE=3.35 msec; flip angle=8°. 256 or 240 (coronal or sagittal) slices. FOV=240 mm. matrix 256x256x256 (or 256x256x240) | 5.1 | Coronal. then sagittal following software upgrade. | Linux- centos4_x86_64- stable-pub-v5.1.0 |
| SHIP- START | 1.5T Siemens Avanto | 3D T1-weighted (MP-RAGE/ axial plane); TR=1900 msec; TE=3.4 msec; Flip angle=15°; voxel size 1 mm x 1 mm x 1 mm | 5.3 (cortical). 5.1 (subcortical) | Axial | Centos6_x86_64 |
| SHIP- TREND | 1.5T Siemens Avanto | 3D T1-weighted (MP-RAGE/ axial plane); TR=1900 msec; TE=3.4 msec; Flip angle=15°; voxel size 1 mm x 1 mm x 1 mm | 5.3 (cortical). 5.1 (subcortical) | Axial | Centos6_x86_64 |
| UCSF | 3T GE Discovery MR750 | SPGR T1-weighted: TR=8.1 ms; TE=3.17 ms; TI=450 ms; flip angle=12°; 256x256 matrix; FOV=250x250 mm; 168 sagittal slices; slice thickness=1 mm; in-plane resolution=0.98x 0.98 mm | 5.3 | Sagittal | Linux-centos6_x86_64-stable-pub-v5.3.0. |
| Sao Paulo | 1.5T General Eletric (GE) | Imaging data were acquired using two MRI scanners (at the Clinics Hospital of the University of Sao Paulo 1.5 T GE Signa scanner. General Electric. Milwaukee Wisconsin. USA). T1-SPGR sequence providing 124 contiguous slices. voxel size 0.8660.8661.5 mm. echo time 5.2 ms. resolution time 21.7 ms. flip angle 20. field of vision 22. matrix 256x192) | 5.3 |  | OsX |
| Singapore | Achieva 3T. Philips Medical Systems. Netherlands | Whole brain high resolution 3D MP-RAGE (magnetisation-prepared rapid acquisition with a gradient echo) volumetric scans (TR/TE/TI/flip angle 8.4/3.8/3000/8; matrix 256x204; FOV 240mm2) with axial orientation (reformatted to coronal) | 5.3 | Axial | Linux_Ubuntu12.04_6 4 |
| SOCAT | 3.0 T. Siemens Verio.Numaris/4.Syngo MR B17.Erlangen.Germany | 3D T1 weighted MP-Rage/axial plane; TR=1900 msec; TE=3.4 msec; Flip angle=15°; Voxel size 1 mm x 1 mm x 1 mm |  | Axial | Ubuntu 18.04 LTS |
| Stanford | 1.5T GE Signa Excite  3.0T GE Discovery MR750 | Whole-brain T1-weighted images were collected using a spoiled gradient echo (SPGR) pulse sequence (116 sagittal slices; through-plane resolution = 1.5 mm; in-plane resolution = 0.86 x 0.86 mm; flip angle = 15 degrees; repetition time [TR] = 8.3-10.1 ms; echo time [TE] = 1.7-3.0; inversion time [TI] = 300 ms; matrix = 256 x 192).  Whole-brain T1-weighted images were collected using a spoiled gradient echo (SPGR) pulse sequence (186 sagittal slices; resolution = 0.9 mm isotropic; flip angle = 12°; repetition time [TR] = 6.240 ms; echo time [TE] = 2.34 ms) | 5.3 | Sagittal | Linux-centos6_x86_64  Centos6_x86_64. Linux-based HPC |

**Table S4. Full mega-analytic results for mean subcortical volume regions for the 1) Group*Age interaction, 2) Group*Sex interaction and 3) main effect of Group controlling for age, sex and ICV.**

|  | **Group*Age interaction** | | | | **Group*Sex interaction** | | | | **Group effect** | | | | **Number of subjects** | | |
| --- | --- | --- | --- | --- | --- | --- | --- | --- | --- | --- | --- | --- | --- | --- | --- |
|  | F | Partial η^2^ | P-value | P_FDR_ | F | Partial η^2^ | P-value | P_FDR_ | F | Partial η^2^ | P-value | P_FDR_ | HC | AD | nAD |
| Lateral Ventricles | 5.4111 | 0.0013 | 0.0045 | 0.0250* | 0.0663 | 0.0000 | 0.9359 | 0.9605 | - | - | - | - | 4974 | 2031 | 1455 |
| Thalamus | 7.9874 | 0.0019 | 0.0003 | 0.0038* | 0.8772 | 0.0002 | 0.4160 | 0.6122 | - | - | - | - | 4968 | 2025 | 1455 |
| Caudate | 0.2435 | 0.0001 | 0.7839 | 0.8992 | 0.5432 | 0.0001 | 0.5809 | 0.7308 | 0.3013 | 0.0001 | 0.7399 | 0.9048 | 4965 | 2030 | 1463 |
| Putamen | 0.1885 | 0.0000 | 0.8282 | 0.9229 | 0.2552 | 0.0001 | 0.7748 | 0.8512 | 0.4629 | 0.0001 | 0.6295 | 0.8533 | 4837 | 1981 | 1397 |
| Pallidum | 4.2371 | 0.0010 | 0.0145 | 0.0594 | 2.4899 | 0.0006 | 0.0830 | 0.2298 | 0.4037 | 0.0001 | 0.6678 | 0.8856 | 4814 | 1979 | 1379 |
| Hippocampus | 4.2980 | 0.0010 | 0.0136 | 0.0590 | 0.6766 | 0.0002 | 0.5084 | 0.7197 | 8.5771 | 0.0020 | 0.0002 | 0.0009* | 4984 | 2027 | 1458 |
| Amygdala | 0.2932 | 0.0001 | 0.7459 | 0.8815 | 0.1333 | 0.0000 | 0.8752 | 0.9321 | 0.7303 | 0.0002 | 0.4818 | 0.7347 | 4968 | 2020 | 1453 |
| Accumbens | 2.6062 | 0.0006 | 0.0739 | 0.1859 | 1.7643 | 0.0004 | 0.1714 | 0.4025 | 0.1166 | 0.0000 | 0.8900 | 0.9211 | 4859 | 2029 | 1430 |

* FDR P-value < 0.05; HC: Healthy controls; AD: cases with current AD use; nAD: cases not currently taking AD

**Table S5.** **Full mega-analytic results for mean cortical thickness regions for the 1) Group*Age interaction, 2) Group*Sex interaction and 3) main effect of Group controlling for age and sex.**

|  |  | **Group*Age interaction** | | |  | **Group*Sex interaction** | | |  | **Group effect** | | | **Number of subjects** | | |
| --- | --- | --- | --- | --- | --- | --- | --- | --- | --- | --- | --- | --- | --- | --- | --- |
|  | F | Partial η^2^ | P-value | P_FDR_ | F | Partial η^2^ | P-value | P_FDR_ | F | Partial η^2^ | P-value | P_FDR_ | HC | AD | nAD |
| Banks superior temporal sulcus | 3.0139 | 0.0008 | 0.0492 | 0.1367 | 1.1848 | 0.0003 | 0.3059 | 0.4970 | 8.1274 | 0.0020 | 0.0003 | 0.0013* | 4738 | 1822 | 1406 |
| Caudal anterior cingulate cortex | 3.2535 | 0.0008 | 0.0387 | 0.1161 | 0.1229 | 0.0000 | 0.8843 | 0.9321 | 6.4270 | 0.0015 | 0.0016 | 0.0062* | 5025 | 2033 | 1483 |
| Caudal middle  frontal gyrus | 7.9773 | 0.0019 | 0.0003 | 0.0038* | 1.5070 | 0.0004 | 0.2216 | 0.4322 | - | - | - | - | 5054 | 2034 | 1480 |
| Cuneus | 4.0016 | 0.0010 | 0.0183 | 0.0666 | 2.2821 | 0.0005 | 0.1021 | 0.2656 | 0.0023 | 0.0000 | 0.9977 | 0.9977 | 4960 | 2003 | 1461 |
| Entorhinal cortex | 0.0075 | 0.0000 | 0.9925 | 0.9925 | 0.9877 | 0.0002 | 0.3725 | 0.5697 | 5.8081 | 0.0015 | 0.0030 | 0.0108* | 4720 | 1847 | 1401 |
| Fusiform gyrus | 1.6037 | 0.0004 | 0.2012 | 0.3488 | 1.1313 | 0.0003 | 0.3226 | 0.5033 | 29.2600 | 0.0068 | 0.0000 | 0.0000* | 5065 | 2025 | 1473 |
| Inferior parietal  cortex | 16.4162 | 0.0039 | 0.0000 | 0.0000* | 2.8919 | 0.0007 | 0.0555 | 0.1859 | - | - | - | - | 5005 | 1993 | 1463 |
| Inferior temporal gyrus | 4.1585 | 0.0010 | 0.0157 | 0.0611 | 0.0171 | 0.0000 | 0.9830 | 0.9958 | 28.4153 | 0.0068 | 0.0000 | 0.0000* | 4962 | 1950 | 1453 |
| Isthmus cingulate cortex | 1.2384 | 0.0003 | 0.2899 | 0.4348 | 0.5689 | 0.0001 | 0.5661 | 0.7308 | 12.8996 | 0.0030 | 0.0000 | 0.0000* | 5048 | 2036 | 1477 |
| Lateral occipital cortex | 10.5294 | 0.0025 | 0.0000 | 0.0007* | 0.6603 | 0.0002 | 0.5167 | 0.7197 | - | - | - | - | 5046 | 2022 | 1477 |
| Lateral orbitofrontal cortex | 3.0472 | 0.0007 | 0.0475 | 0.1367 | 1.3115 | 0.0003 | 0.2695 | 0.4702 | 14.4569 | 0.0034 | 0.0000 | 0.0000* | 5054 | 2038 | 1481 |
| Lingual gyrus | 2.9217 | 0.0007 | 0.0539 | 0.1401 | 0.4545 | 0.0001 | 0.6348 | 0.7689 | 3.5365 | 0.0008 | 0.0292 | 0.0847 | 5024 | 2026 | 1465 |
| Medial orbitofrontal cortex | 0.7988 | 0.0002 | 0.4499 | 0.6157 | 0.4452 | 0.0001 | 0.6407 | 0.7689 | 21.1512 | 0.0050 | 0.0000 | 0.0000* | 4993 | 2028 | 1466 |
| Middle temporal gyrus | 8.1759 | 0.0020 | 0.0003 | 0.0038* | 1.4518 | 0.0004 | 0.2342 | 0.4350 | - | - | - | - | 4871 | 1896 | 1434 |
| Parahippocampal gyrus | 0.0578 | 0.0000 | 0.9439 | 0.9816 | 0.0042 | 0.0000 | 0.9958 | 0.9958 | 8.9445 | 0.0021 | 0.0001 | 0.0007* | 5071 | 2037 | 1477 |
| Paracentral lobule | 2.9803 | 0.0007 | 0.0508 | 0.1367 | 2.8805 | 0.0007 | 0.0562 | 0.1859 | 3.2281 | 0.0008 | 0.0397 | 0.1052 | 5065 | 2041 | 1480 |
| Pars opercularis | 1.8647 | 0.0004 | 0.1550 | 0.3023 | 1.1320 | 0.0003 | 0.3224 | 0.5033 | 16.2287 | 0.0038 | 0.0000 | 0.0000* | 5035 | 2029 | 1471 |
| Pars orbitalis | 4.8857 | 0.0011 | 0.0076 | 0.0348* | 1.5421 | 0.0004 | 0.2140 | 0.4280 | - | - | - | - | 5065 | 2035 | 1480 |
| Pars triangularis | 5.5883 | 0.0013 | 0.0038 | 0.0225* | 0.3157 | 0.0001 | 0.7293 | 0.8366 | - | - | - | - | 5033 | 2030 | 1479 |
| Pericalcarine cortex | 1.9850 | 0.0005 | 0.1374 | 0.2898 | 0.6152 | 0.0001 | 0.5405 | 0.7269 | 1.7267 | 0.0004 | 0.1779 | 0.3743 | 4978 | 1994 | 1469 |
| Postcentral gyrus | 9.9654 | 0.0023 | 0.0000 | 0.0009* | 2.5971 | 0.0006 | 0.0745 | 0.2236 | - | - | - | - | 4991 | 2025 | 1467 |
| Posterior cingulate cortex | 1.0434 | 0.0002 | 0.3523 | 0.5089 | 0.2610 | 0.0001 | 0.7703 | 0.8512 | 12.1793 | 0.0028 | 0.0000 | 0.0000* | 5053 | 2044 | 1481 |
| Precentral gyrus | 5.1316 | 0.0012 | 0.0059 | 0.0308* | 0.5970 | 0.0001 | 0.5505 | 0.7277 | - | - | - | - | 5003 | 2021 | 1465 |
| Precuneus | 3.8457 | 0.0009 | 0.0214 | 0.0696 | 1.6613 | 0.0004 | 0.1899 | 0.4116 | 4.6506 | 0.0011 | 0.0096 | 0.0308* | 5041 | 2041 | 1476 |
| Rostral anterior cingulate cortex | 3.9761 | 0.0009 | 0.0188 | 0.0666 | 0.2939 | 0.0001 | 0.7454 | 0.8426 | 15.8645 | 0.0037 | 0.0000 | 0.0000* | 4991 | 2031 | 1471 |
| Rostral middle frontal gyrus | 6.9579 | 0.0016 | 0.0010 | 0.0083* | 0.2237 | 0.0001 | 0.7995 | 0.8662 | - | - | - | - | 5042 | 2027 | 1480 |
| Superior frontal gyrus | 5.9863 | 0.0014 | 0.0025 | 0.0179* | 1.4557 | 0.0003 | 0.2333 | 0.4350 | - | - | - | - | 5048 | 2032 | 1478 |
| Superior parietal cortex | 12.2876 | 0.0029 | 0.0000 | 0.0002* | 1.9239 | 0.0005 | 0.1461 | 0.3561 | - | - | - | - | 5038 | 2033 | 1475 |
| Superior temporal gyrus | 2.1634 | 0.0005 | 0.1150 | 0.2563 | 1.5844 | 0.0004 | 0.2051 | 0.4211 | 15.6393 | 0.0039 | 0.0000 | 0.0000* | 4705 | 1800 | 1395 |
| Supramarginal gyrus | 5.7872 | 0.0014 | 0.0031 | 0.0200* | 1.2082 | 0.0003 | 0.2988 | 0.4970 | - | - | - | - | 4878 | 1917 | 1446 |
| Frontal pole | 1.5021 | 0.0004 | 0.2227 | 0.3662 | 0.3931 | 0.0001 | 0.6750 | 0.7977 | 7.7762 | 0.0018 | 0.0004 | 0.0017* | 5060 | 2038 | 1477 |
| Temporal pole | 1.6417 | 0.0004 | 0.1937 | 0.3434 | 0.8228 | 0.0002 | 0.4392 | 0.6344 | 2.2904 | 0.0005 | 0.1013 | 0.2288 | 5002 | 2030 | 1464 |
| Transverse temporal gyrus | 0.6903 | 0.0002 | 0.5015 | 0.6744 | 0.4930 | 0.0001 | 0.6108 | 0.7563 | 9.0753 | 0.0021 | 0.0001 | 0.0006* | 5052 | 2043 | 1477 |
| Insula | 3.8670 | 0.0009 | 0.0210 | 0.0696 | 3.1438 | 0.0008 | 0.0432 | 0.1859 | 33.1420 | 0.0079 | 0.0000 | 0.0000* | 4881 | 1999 | 1464 |
| Average thickness | 7.8693 | 0.0018 | 0.0004 | 0.0038* | 0.9553 | 0.0002 | 0.3848 | 0.5771 | - | - | - | - | 5092 | 2049 | 1485 |

* FDR P-value < 0.05; HC: Healthy controls; AD: cases with current AD use; nAD: cases not currently taking AD

**Table S6.** **Full mega-analytic results for mean cortical surface area regions for the 1) Group*Age interaction, 2) Group*Sex interaction and 3) main effect of Group controlling for age, sex and ICV.**

|  |  | **Group*Age interaction** | | |  | **Group*Sex interaction** | | |  | **Group effect** | | | **Number of subjects** | | |
| --- | --- | --- | --- | --- | --- | --- | --- | --- | --- | --- | --- | --- | --- | --- | --- |
|  | F | Partial η^2^ | P-value | P_FDR_ | F | Partial η^2^ | P-value | P_FDR_ | F | Partial η^2^ | P-value | P_FDR_ | HC | AD | nAD |
| Banks superior temporal sulcus | 0.3334 | 0.0001 | 0.7165 | 0.8732 | 5.3380 | 0.0014 | 0.0048 | 0.0887 | 3.2505 | 0.0008 | 0.0388 | 0.1052 | 4621 | 1813 | 1371 |
| Caudal anterior cingulate cortex | 2.1967 | 0.0005 | 0.1112 | 0.2552 | 1.2028 | 0.0003 | 0.3004 | 0.4970 | 0.6562 | 0.0002 | 0.5188 | 0.7719 | 4938 | 2015 | 1457 |
| Caudal middle  frontal gyrus | 0.5371 | 0.0001 | 0.5844 | 0.7473 | 2.2435 | 0.0005 | 0.1061 | 0.2671 | 0.0988 | 0.0000 | 0.9060 | 0.9211 | 4978 | 2027 | 1457 |
| Cuneus | 0.1963 | 0.0000 | 0.8218 | 0.9229 | 0.5507 | 0.0001 | 0.5765 | 0.7308 | 0.3415 | 0.0001 | 0.7107 | 0.9032 | 4920 | 2003 | 1446 |
| Entorhinal cortex | 2.3707 | 0.0006 | 0.0935 | 0.2210 | 3.7044 | 0.0010 | 0.0247 | 0.1703 | 0.5812 | 0.0002 | 0.5593 | 0.8123 | 4434 | 1835 | 1329 |
| Fusiform gyrus | 1.7812 | 0.0004 | 0.1685 | 0.3144 | 0.6409 | 0.0002 | 0.5268 | 0.7209 | 3.1425 | 0.0008 | 0.0432 | 0.1099 | 4851 | 2021 | 1426 |
| Inferior parietal  cortex | 2.5599 | 0.0006 | 0.0774 | 0.1886 | 3.1802 | 0.0008 | 0.0416 | 0.1859 | 0.8133 | 0.0002 | 0.4434 | 0.7217 | 4896 | 1992 | 1444 |
| Inferior temporal gyrus | 0.6639 | 0.0002 | 0.5149 | 0.6807 | 5.1725 | 0.0012 | 0.0057 | 0.0887 | 0.2703 | 0.0001 | 0.7632 | 0.9048 | 4942 | 1949 | 1456 |
| Isthmus cingulate cortex | 0.6124 | 0.0001 | 0.5421 | 0.7047 | 3.4204 | 0.0008 | 0.0327 | 0.1703 | 4.8515 | 0.0011 | 0.0078 | 0.0266* | 5028 | 2029 | 1472 |
| Lateral occipital cortex | 5.0346 | 0.0012 | 0.0065 | 0.0318* | 1.7407 | 0.0004 | 0.1755 | 0.4025 | - | - | - | - | 5025 | 2023 | 1474 |
| Lateral orbitofrontal cortex | 1.0049 | 0.0002 | 0.3661 | 0.5192 | 2.4608 | 0.0006 | 0.0854 | 0.2298 | 1.5594 | 0.0004 | 0.2103 | 0.4138 | 5074 | 2041 | 1481 |
| Lingual gyrus | 1.8946 | 0.0004 | 0.1504 | 0.3023 | 1.3704 | 0.0003 | 0.2541 | 0.4608 | 2.8608 | 0.0007 | 0.0573 | 0.1380 | 5032 | 2029 | 1471 |
| Medial orbitofrontal cortex | 3.3694 | 0.0008 | 0.0345 | 0.1075 | 0.3205 | 0.0001 | 0.7258 | 0.8366 | 0.4852 | 0.0001 | 0.6156 | 0.8533 | 4924 | 2033 | 1427 |
| Middle temporal gyrus | 0.3160 | 0.0001 | 0.7291 | 0.8749 | 4.3103 | 0.0011 | 0.0135 | 0.1703 | 0.1523 | 0.0000 | 0.8588 | 0.9211 | 4765 | 1893 | 1405 |
| Parahippocampal gyrus | 1.0810 | 0.0003 | 0.3393 | 0.4993 | 2.9105 | 0.0007 | 0.0545 | 0.1859 | 3.7181 | 0.0009 | 0.0243 | 0.0742 | 4950 | 2016 | 1451 |
| Paracentral lobule | 1.4622 | 0.0003 | 0.2318 | 0.3690 | 3.4537 | 0.0008 | 0.0317 | 0.1703 | 1.4561 | 0.0003 | 0.2332 | 0.4446 | 4936 | 2037 | 1448 |
| Pars opercularis | 0.4077 | 0.0001 | 0.6652 | 0.8307 | 5.8402 | 0.0014 | 0.0029 | 0.0887 | 0.9159 | 0.0002 | 0.4002 | 0.6975 | 4951 | 2024 | 1456 |
| Pars orbitalis | 0.1199 | 0.0000 | 0.8870 | 0.9609 | 1.3048 | 0.0003 | 0.2713 | 0.4702 | 1.5920 | 0.0004 | 0.2036 | 0.4138 | 5044 | 2035 | 1472 |
| Pars triangularis | 0.1425 | 0.0000 | 0.8672 | 0.9527 | 3.2139 | 0.0008 | 0.0402 | 0.1859 | 0.2917 | 0.0001 | 0.7470 | 0.9048 | 4996 | 2035 | 1471 |
| Pericalcarine cortex | 0.2590 | 0.0001 | 0.7718 | 0.8986 | 0.0783 | 0.0000 | 0.9247 | 0.9605 | 1.7274 | 0.0004 | 0.1778 | 0.3743 | 4996 | 2001 | 1471 |
| Postcentral gyrus | 1.7764 | 0.0004 | 0.1693 | 0.3144 | 3.0213 | 0.0007 | 0.0488 | 0.1859 | 0.1043 | 0.0000 | 0.9009 | 0.9211 | 4896 | 2017 | 1436 |
| Posterior cingulate cortex | 1.2986 | 0.0003 | 0.2730 | 0.4217 | 1.6008 | 0.0004 | 0.2018 | 0.4211 | 0.7328 | 0.0002 | 0.4806 | 0.7347 | 5042 | 2041 | 1476 |
| Precentral gyrus | 1.4903 | 0.0004 | 0.2254 | 0.3662 | 2.6944 | 0.0006 | 0.0676 | 0.2110 | 0.8560 | 0.0002 | 0.4249 | 0.7200 | 4919 | 2021 | 1448 |
| Precuneus | 0.0091 | 0.0000 | 0.9909 | 0.9925 | 3.6236 | 0.0009 | 0.0267 | 0.1703 | 1.3637 | 0.0003 | 0.2558 | 0.4728 | 5012 | 2038 | 1473 |
| Rostral anterior cingulate cortex | 1.7511 | 0.0004 | 0.1736 | 0.3150 | 3.5254 | 0.0009 | 0.0295 | 0.1703 | 0.2218 | 0.0001 | 0.8010 | 0.9048 | 4851 | 2019 | 1423 |
| Rostral middle frontal gyrus | 0.3991 | 0.0001 | 0.6710 | 0.8307 | 3.6144 | 0.0009 | 0.0270 | 0.1703 | 0.2299 | 0.0001 | 0.7946 | 0.9048 | 5011 | 2025 | 1470 |
| Superior frontal gyrus | 0.9445 | 0.0002 | 0.3889 | 0.5417 | 3.6002 | 0.0009 | 0.0274 | 0.1703 | 0.2036 | 0.0000 | 0.8158 | 0.9048 | 4905 | 2022 | 1448 |
| Superior parietal cortex | 1.5589 | 0.0004 | 0.2104 | 0.3568 | 2.8622 | 0.0007 | 0.0572 | 0.1859 | 0.3502 | 0.0001 | 0.7046 | 0.9032 | 4961 | 2027 | 1455 |
| Superior temporal gyrus | 0.0758 | 0.0000 | 0.9270 | 0.9816 | 5.2131 | 0.0013 | 0.0055 | 0.0887 | 0.1395 | 0.0000 | 0.8698 | 0.9211 | 4623 | 1800 | 1367 |
| Supramarginal gyrus | 1.8685 | 0.0005 | 0.1544 | 0.3023 | 2.5401 | 0.0006 | 0.0789 | 0.2280 | 0.7995 | 0.0002 | 0.4496 | 0.7217 | 4742 | 1912 | 1418 |
| Frontal pole | 0.0596 | 0.0000 | 0.9421 | 0.9816 | 3.8854 | 0.0009 | 0.0206 | 0.1703 | 0.9925 | 0.0002 | 0.3707 | 0.6651 | 5062 | 2045 | 1482 |
| Temporal pole | 1.2886 | 0.0003 | 0.2757 | 0.4217 | 4.1284 | 0.0010 | 0.0161 | 0.1703 | 2.8345 | 0.0007 | 0.0588 | 0.1380 | 4947 | 2035 | 1456 |
| Transverse temporal gyrus | 0.0367 | 0.0000 | 0.9640 | 0.9893 | 3.0135 | 0.0007 | 0.0492 | 0.1859 | 0.2244 | 0.0001 | 0.7990 | 0.9048 | 5075 | 2048 | 1479 |
| Insula | 6.0253 | 0.0015 | 0.0024 | 0.0179* | 1.6801 | 0.0004 | 0.1864 | 0.4116 | - | - | - | - | 4860 | 1996 | 1458 |
| Total surface area | 2.0891 | 0.0005 | 0.1239 | 0.2684 | 5.3835 | 0.0012 | 0.0046 | 0.0887 | 0.5221 | 0.0001 | 0.5933 | 0.8416 | 5100 | 2055 | 1488 |

* FDR P-value < 0.05; HC: Healthy controls; AD: cases with current AD use; nAD: cases not currently taking AD

**Table S7. Secondary analyses for mean cortical thickness regions: Group*Age interaction within the sample of patients with MDD controlling for age and sex.**

|  |  | **AD-nAD*Age interaction** | | | **Number of subjects** | | |
| --- | --- | --- | --- | --- | --- | --- | --- |
|  | F | Partial η^2^ | P-value | P_FDR_ | AD | nAD |  |
| Banks superior temporal sulcus | 3.4015 | 0.0011 | 0.0652 | 0.2811 | 1822 | 1406 |  |
| Caudal anterior cingulate cortex | 3.5022 | 0.0010 | 0.0614 | 0.2811 | 2033 | 1483 |  |
| Caudal middle  frontal gyrus | 3.8706 | 0.0011 | 0.0492 | 0.2811 | 2034 | 1480 |  |
| Cuneus | 0.0694 | 0.0000 | 0.7922 | 0.8980 | 2003 | 1461 |  |
| Entorhinal cortex | 0.0102 | 0.0000 | 0.9196 | 0.9315 | 1847 | 1401 |  |
| Fusiform gyrus | 1.4249 | 0.0004 | 0.2327 | 0.4627 | 2025 | 1473 |  |
| Inferior parietal  cortex | 6.4486 | 0.0019 | 0.0111 | 0.1460 | 1993 | 1463 |  |
| Inferior temporal gyrus | 1.6524 | 0.0005 | 0.1987 | 0.4583 | 1950 | 1453 |  |
| Isthmus cingulate cortex | 2.1241 | 0.0006 | 0.1451 | 0.4012 | 2036 | 1477 |  |
| Lateral occipital cortex | 2.8740 | 0.0008 | 0.0901 | 0.2811 | 2022 | 1477 |  |
| Lateral orbitofrontal cortex | 2.8884 | 0.0008 | 0.0893 | 0.2811 | 2038 | 1481 |  |
| Lingual gyrus | 0.0564 | 0.0000 | 0.8123 | 0.8980 | 2026 | 1465 |  |
| Medial orbitofrontal cortex | 1.6240 | 0.0005 | 0.2026 | 0.4583 | 2028 | 1466 |  |
| Middle temporal gyrus | 13.3251 | 0.0040 | 0.0003 | 0.0207* | 1896 | 1434 |  |
| Parahippocampal gyrus | 0.1145 | 0.0000 | 0.7351 | 0.8980 | 2037 | 1477 |  |
| Paracentral lobule | 0.3502 | 0.0001 | 0.5540 | 0.7889 | 2041 | 1480 |  |
| Pars opercularis | 1.6684 | 0.0005 | 0.1966 | 0.4583 | 2029 | 1471 |  |
| Pars orbitalis | 0.7254 | 0.0002 | 0.3944 | 0.6546 | 2035 | 1480 |  |
| Pars triangularis | 7.7799 | 0.0022 | 0.0053 | 0.1295 | 2030 | 1479 |  |
| Pericalcarine cortex | 0.4380 | 0.0001 | 0.5081 | 0.7646 | 1994 | 1469 |  |
| Postcentral gyrus | 0.0608 | 0.0000 | 0.8053 | 0.8980 | 2025 | 1467 |  |
| Posterior cingulate cortex | 0.3463 | 0.0001 | 0.5563 | 0.7889 | 2044 | 1481 |  |
| Precentral gyrus | 0.5141 | 0.0001 | 0.4734 | 0.7536 | 2021 | 1465 |  |
| Precuneus | 0.0743 | 0.0000 | 0.7852 | 0.8980 | 2041 | 1476 |  |
| Rostral anterior cingulate cortex | 3.2687 | 0.0009 | 0.0707 | 0.2811 | 2031 | 1471 |  |
| Rostral middle frontal gyrus | 1.3973 | 0.0004 | 0.2373 | 0.4627 | 2027 | 1480 |  |
| Superior frontal gyrus | 0.5957 | 0.0002 | 0.4403 | 0.7154 | 2032 | 1478 |  |
| Superior parietal cortex | 0.1330 | 0.0000 | 0.7154 | 0.8980 | 2033 | 1475 |  |
| Superior temporal gyrus | 0.7489 | 0.0002 | 0.3869 | 0.6546 | 1800 | 1395 |  |
| Supramarginal gyrus | 0.3029 | 0.0001 | 0.5821 | 0.8057 | 1917 | 1446 |  |
| Frontal pole | 2.1853 | 0.0006 | 0.1394 | 0.4012 | 2038 | 1477 |  |
| Temporal pole | 3.2456 | 0.0009 | 0.0717 | 0.2811 | 2030 | 1464 |  |
| Transverse temporal gyrus | 0.0829 | 0.0000 | 0.7734 | 0.8980 | 2043 | 1477 |  |
| Insula | 4.1667 | 0.0012 | 0.0413 | 0.2811 | 1999 | 1464 |  |
| Average thickness | 1.4351 | 0.0004 | 0.2310 | 0.4627 | 2049 | 1485 |  |

* FDR P-value < 0.05; AD: cases with current AD use; nAD: cases not currently taking AD

**Table S8.** **Secondary analyses for mean cortical thickness of the middle temporal gyrus: Group*Age interaction within the sample of patients with MDD controlling for age, sex and HDRS-17 score.**

|  |  | **AD-nAD*Age interaction** | | | **Number of subjects** | | |
| --- | --- | --- | --- | --- | --- | --- | --- |
|  | F | Partial η^2^ | P-value | P_FDR_ | AD | nAD |  |
| Middle temporal gyrus | 8.7170 | 0.0045 | 0.0032 | 0.0032* | 1412 | 507 |  |

* FDR P-value < 0.05; AD: cases with current AD use; nAD: cases not currently taking AD

**Table S9.** **Secondary analyses for mean cortical thickness of the middle temporal gyrus: Group*Age interaction within the sample of patients with MDD controlling for age, sex and BDI-II score.**

|  |  | **AD-nAD*Age interaction** | | | **Number of subjects** | | |
| --- | --- | --- | --- | --- | --- | --- | --- |
|  | F | Partial η^2^ | P-value | P_FDR_ | AD | nAD |  |
| Middle temporal gyrus | 0.1538 | 0.0001 | 0.6950 | 0.6950 | 534 | 598 |  |

AD: cases with current AD use; nAD: cases not currently taking AD

**Table S10.** **Secondary analyses for mean cortical thickness of the middle temporal gyrus: Group*Age interaction within the sample of patients with MDD controlling for age, sex and number of depressive episodes.**

|  |  | **AD-nAD*Age interaction** | | | **Number of subjects** | | |
| --- | --- | --- | --- | --- | --- | --- | --- |
|  | F | Partial η^2^ | P-value | P_FDR_ | AD | nAD |  |
| Middle temporal gyrus | 13.7148 | 0.0059 | 0.0002 | 0.0002* | 1489 | 820 |  |

* FDR P-value < 0.05; AD: cases with current AD use; nAD: cases not currently taking AD

**Table S11.** **Secondary analyses for mean cortical thickness of the middle temporal gyrus: Group*Age interaction within the sample of patients with MDD controlling for age, sex and stage of illness**.

|  |  | **AD-nAD*Age interaction** | | | **Number of subjects** | | |
| --- | --- | --- | --- | --- | --- | --- | --- |
|  | F | Partial η^2^ | P-value | P_FDR_ | AD | nAD |  |
| Middle temporal gyrus | 8.3985 | 0.0033 | 0.0038 | 0.0038* | 1328 | 1197 |  |

* FDR P-value < 0.05; AD: cases with current AD use; nAD: cases not currently taking AD

**Table S12.** **Secondary analyses for mean cortical thickness of the middle temporal gyrus: Group*Age interaction within the sample of patients with MDD controlling for age, sex and remission status.**

|  |  | **AD-nAD*Age interaction** | | | **Number of subjects** | | |
| --- | --- | --- | --- | --- | --- | --- | --- |
|  | F | Partial η^2^ | P-value | P_FDR_ | AD | nAD |  |
| Middle temporal gyrus | 19.2813 | 0.0074 | 0.0000 | 0.0000* | 1699 | 905 |  |

* FDR P-value < 0.05; AD: cases with current AD use; nAD: cases not currently taking AD

**Table S13.** **Mega-analytic results for mean subcortical volume of the hippocampus for the AD versus nAD group comparison controlling for age, sex and ICV.**

|  | Cohen’s d (AD-nAD) | Std. Err. | 95 % CI | P-value | P_FDR_ | No. of AD | No. of nAD |
| --- | --- | --- | --- | --- | --- | --- | --- |
| Hippocampus | -0.1300 | 0.0344 | -0.1974 - -0.0627 | 0.0002 | 0.0015* | 2027 | 1458 |

* FDR P-value < 0.05; AD: cases with current AD use; nAD: cases not currently taking AD

**Table S14. Secondary analyses for mean subcortical volume of the hippocampus: AD versus nAD group comparison controlling for age, sex, ICV and HDRS-17 score.**

|  | Cohen’s d (AD-nAD) | Std. Err. | 95 % CI | P-value | P_FDR_ | No. of AD | No. of nAD |
| --- | --- | --- | --- | --- | --- | --- | --- |
| Hippocampus | -0.0984 | 0.0344 | -0.1657 - -0.0311 | 0.0281 | 0.0760 | 1530 | 543 |

AD: cases with current AD use; nAD: cases not currently taking AD

**Table S15. Secondary analyses for mean subcortical volume of the hippocampus: AD versus nAD group comparison controlling for age, sex, ICV and BDI-II score.**

|  | Cohen’s d (AD-nAD) | Std. Err. | 95 % CI | P-value | P_FDR_ | No. of AD | No. of nAD |
| --- | --- | --- | --- | --- | --- | --- | --- |
| Hippocampus | -0.0914 | 0.0344 | -0.1587 - -0.0240 | 0.1228 | 0.2047 | 576 | 606 |

AD: cases with current AD use; nAD: cases not currently taking AD

**Table S16. Secondary analyses for mean subcortical volume of the hippocampus: AD versus nAD group comparison controlling for age, sex, ICV and number of depressive episodes.**

|  | Cohen’s d (AD-nAD) | Std. Err. | 95 % CI | P-value | P_FDR_ | No. of AD | No. of nAD |
| --- | --- | --- | --- | --- | --- | --- | --- |
| Hippocampus | -0.1475 | 0.0344 | -0.2149 - -0.0801 | 0.0003 | 0.0027* | 1662 | 868 |

* FDR P-value < 0.05; AD: cases with current AD use; nAD: cases not currently taking AD

**Table S17. Secondary analyses for mean subcortical volume of the hippocampus: AD versus nAD group comparison controlling for age, sex, ICV and stage of illness.**

|  | Cohen’s d (AD-nAD) | Std. Err. | 95 % CI | P-value | P_FDR_ | No. of AD | No. of nAD |
| --- | --- | --- | --- | --- | --- | --- | --- |
| Hippocampus | -0.1594 | 0.0344 | -0.2268 - -0.0920 | 0.0001 | 0.0006* | 1419 | 1212 |

* FDR P-value < 0.05; AD: cases with current AD use; nAD: cases not currently taking AD

**Table S18. Secondary analyses for mean subcortical volume of the hippocampus: AD versus nAD group comparison controlling for age, sex, ICV and remission status.**

|  | Cohen’s d (AD-nAD) | Std. Err. | 95 % CI | P-value | P_FDR_ | No. of AD | No. of nAD |
| --- | --- | --- | --- | --- | --- | --- | --- |
| Hippocampus | -0.1287 | 0.0344 | -0.1961 - -0.0613 | 0.0009 | 0.0087* | 1831 | 943 |

* FDR P-value < 0.05; AD: cases with current AD use; nAD: cases not currently taking AD

**Table S19.** **Mega-analytic results for mean cortical thickness regions for the AD versus nAD group comparison controlling for age and sex.**

|  | Cohen’s d (AD-nAD) | Std. Err. | 95 % CI | P-value | P_FDR_ | No. of AD | No. of nAD |
| --- | --- | --- | --- | --- | --- | --- | --- |
| Banks superior temporal sulcus | -0.0388 | 0.0355 | -0.1084 - 0.0307 | 0.2743 | 0.3474 | 1822 | 1406 |
| Caudal anterior cingulate cortex | -0.0411 | 0.0342 | -0.1080 - 0.0259 | 0.2295 | 0.3114 | 2033 | 1483 |
| Entorhinal cortex | -0.0562 | 0.0354 | -0.1256 - 0.0133 | 0.1132 | 0.1792 | 1847 | 1401 |
| Fusiform gyrus | -0.0863 | 0.0343 | -0.1534 - -0.0191 | 0.0118 | 0.0281* | 2025 | 1473 |
| Inferior temporal gyrus | -0.1349 | 0.0347 | -0.2029 - -0.0669 | 0.0001 | 0.0015* | 1950 | 1453 |
| Isthmus cingulate cortex | -0.0563 | 0.0342 | -0.1233 - 0.0107 | 0.0998 | 0.1723 | 2036 | 1477 |
| Lateral orbitofrontal cortex | -0.0834 | 0.0342 | -0.1504 - -0.0165 | 0.0147 | 0.0309* | 2038 | 1481 |
| Medial orbitofrontal cortex | -0.1015 | 0.0343 | -0.1687 - -0.0342 | 0.0031 | 0.0095* | 2028 | 1466 |
| Parahippocampal gyrus | -0.0999 | 0.0342 | -0.1669 - -0.0329 | 0.0035 | 0.0095* | 2037 | 1477 |
| Pars opercularis | -0.0765 | 0.0343 | -0.1436 - -0.0094 | 0.0256 | 0.0487* | 2029 | 1471 |
| Posterior cingulate cortex | -0.0332 | 0.0341 | -0.1001 - 0.0337 | 0.3311 | 0.3495 | 2044 | 1481 |
| Precuneus | -0.0435 | 0.0342 | -0.1104 - 0.0235 | 0.2037 | 0.2977 | 2041 | 1476 |
| Rostral anterior cingulate cortex | -0.0214 | 0.0342 | -0.0885 - 0.0457 | 0.5323 | 0.5323 | 2031 | 1471 |
| Superior temporal gyrus | -0.1286 | 0.0357 | -0.1986 - -0.0586 | 0.0003 | 0.0020* | 1800 | 1395 |
| Frontal pole | -0.1200 | 0.0342 | -0.1870 - -0.0529 | 0.0005 | 0.0022* | 2038 | 1477 |
| Transverse temporal gyrus | -0.0335 | 0.0342 | -0.1004 - 0.0335 | 0.3276 | 0.3495 | 2043 | 1477 |
| Insula | -0.1107 | 0.0344 | -0.1782 - -0.0433 | 0.0013 | 0.0050* | 1999 | 1464 |

* FDR P-value < .05; AD: cases with current AD use; nAD: cases not currently taking AD

**Table S20. Secondary analyses for mean cortical thickness regions: AD versus nAD group comparison controlling for age, sex and HDRS-17 score.**

|  | Cohen’s d (AD-nAD) | Std. Err. | 95 % CI | P-value | P_FDR_ | No. of AD | No. of nAD |
| --- | --- | --- | --- | --- | --- | --- | --- |
| Fusiform gyrus | -0.0840 | 0.0343 | -0.1512 - -0.0169 | 0.0601 | 0.1002 | 1529 | 532 |
| Inferior temporal gyrus | -0.1000 | 0.0347 | -0.1679 - -0.0320 | 0.0283 | 0.0760 | 1462 | 514 |
| Lateral orbitofrontal cortex | -0.0483 | 0.0342 | -0.1152 - 0.0187 | 0.2779 | 0.2779 | 1545 | 535 |
| Medial orbitofrontal cortex | -0.1100 | 0.0343 | -0.1773 - 0.0428 | 0.0138 | 0.0760 | 1538 | 527 |
| Parahippocampal gyrus | -0.0749 | 0.0342 | -0.1419 - -0.0079 | 0.0932 | 0.1165 | 1535 | 534 |
| Pars opercularis | -0.0763 | 0.0343 | -0.1434 - -0.0091 | 0.0877 | 0.1165 | 1534 | 529 |
| Superior temporal gyrus | -0.1025 | 0.0357 | -0.1724 - -0.0325 | 0.0304 | 0.0760 | 1351 | 472 |
| Frontal pole | -0.0897 | 0.0342 | -0.1567 - -0.0227 | 0.0440 | 0.0881 | 1541 | 534 |
| Insula | -0.0646 | 0.0344 | -0.1320 - 0.0029 | 0.1518 | 0.1687 | 1503 | 521 |

AD: cases with current AD use; nAD: cases not currently taking AD

**Table S21. Secondary analyses for mean cortical thickness regions: AD versus nAD group comparison controlling for age, sex and BDI-II score.**

|  | Cohen’s d (AD-nAD) | Std. Err. | 95 % CI | P-value | P_FDR_ | No. of AD | No. of nAD |
| --- | --- | --- | --- | --- | --- | --- | --- |
| Fusiform gyrus | -0.1165 | 0.0343 | -0.1837 - -0.0493 | 0.0478 | 0.1206 | 569 | 622 |
| Inferior temporal gyrus | -0.1424 | 0.0347 | -0.2104 - -0.0744 | 0.0172 | 0.1178 | 543 | 609 |
| Lateral orbitofrontal cortex | -0.0517 | 0.0342 | -0.1187 - 0.0152 | 0.3777 | 0.3777 | 573 | 626 |
| Medial orbitofrontal cortex | -0.0848 | 0.0343 | -0.1520 - -0.0176 | 0.1494 | 0.2134 | 575 | 618 |
| Parahippocampal gyrus | -0.1159 | 0.0342 | -0.1829 - -0.0488 | 0.0482 | 0.1206 | 579 | 621 |
| Pars opercularis | -0.1008 | 0.0343 | -0.1679 - -0.0336 | 0.0876 | 0.1751 | 570 | 617 |
| Superior temporal gyrus | -0.0737 | 0.0357 | -0.1436 - -0.0038 | 0.2259 | 0.2823 | 510 | 594 |
| Frontal pole | -0.1326 | 0.0342 | -0.1997 - -0.0656 | 0.0236 | 0.1178 | 579 | 625 |
| Insula | -0.0664 | 0.0344 | -0.1339 - 0.0010 | 0.2604 | 0.2893 | 559 | 623 |

AD: cases with current AD use; nAD: cases not currently taking AD

**Table S22. Secondary analyses for mean cortical thickness regions: AD versus nAD group comparison controlling for age, sex and number of depressive episodes.**

|  | Cohen’s d (AD-nAD) | Std. Err. | 95 % CI | P-value | P_FDR_ | No. of AD | No. of nAD |
| --- | --- | --- | --- | --- | --- | --- | --- |
| Fusiform gyrus | -0.0590 | 0.0343 | -0.1262 - 0.0081 | 0.1498 | 0.1873 | 1604 | 844 |
| Inferior temporal gyrus | -0.1144 | 0.0347 | -0.1823 - -0.0464 | 0.0061 | 0.0202* | 1535 | 828 |
| Lateral orbitofrontal cortex | -0.0506 | 0.0342 | -0.1176 - 0.0163 | 0.2150 | 0.2302 | 1618 | 849 |
| Medial orbitofrontal cortex | -0.1349 | 0.0343 | -0.2021 - -0.0676 | 0.0010 | 0.0050* | 1613 | 840 |
| Parahippocampal gyrus | -0.0597 | 0.0342 | -0.1266 - 0.0073 | 0.1445 | 0.1873 | 1617 | 846 |
| Pars opercularis | -0.0491 | 0.0342 | -0.1163 - 0.0180 | 0.2302 | 0.2302 | 1609 | 843 |
| Superior temporal gyrus | -0.0885 | 0.0357 | -0.1584 - -0.0185 | 0.0390 | 0.0650 | 1429 | 793 |
| Frontal pole | -0.0928 | 0.0342 | -0.1598 - -0.0258 | 0.0233 | 0.0581 | 1616 | 846 |
| Insula | -0.0886 | 0.0344 | -0.1561 - -0.0212 | 0.0317 | 0.0634 | 1581 | 835 |

* FDR P-value < 0.05; AD: cases with current AD use; nAD: cases not currently taking AD

**Table S23.** **Secondary analyses for mean cortical thickness regions: AD versus nAD group comparison controlling for age, sex and stage of illness.**

|  | Cohen’s d (AD-nAD) | Std. Err. | 95 % CI | P-value | P_FDR_ | No. of AD | No. of nAD |
| --- | --- | --- | --- | --- | --- | --- | --- |
| Fusiform gyrus | -0.0912 | 0.0343 | -0.1584 - -0.0241 | 0.0207 | 0.0345* | 1420 | 1227 |
| Inferior temporal gyrus | -0.1433 | 0.0347 | -0.2113 - -0.0753 | 0.0003 | 0.0014* | 1371 | 1216 |
| Lateral orbitofrontal cortex | -0.0683 | 0.0342 | -0.1352 - -0.0013 | 0.0824 | 0.0824 | 1427 | 1235 |
| Medial orbitofrontal cortex | -0.0786 | 0.0343 | -0.1458 - -0.0114 | 0.0467 | 0.0584 | 1419 | 1221 |
| Parahippocampal gyrus | -0.1102 | 0.0342 | -0.1772 - -0.0431 | 0.0051 | 0.0103* | 1428 | 1232 |
| Pars opercularis | -0.0750 | 0.0343 | -0.1421 - -0.0078 | 0.0575 | 0.0638 | 1418 | 1226 |
| Superior temporal gyrus | -0.1461 | 0.0357 | -0.2161 - -0.0761 | 0.0004 | 0.0014* | 1226 | 1159 |
| Frontal pole | -0.1316 | 0.0342 | -0.1987 - -0.0646 | 0.0008 | 0.0021* | 1428 | 1233 |
| Insula | -0.0841 | 0.0344 | -0.1516 - -0.0167 | 0.0340 | 0.0486* | 1390 | 1222 |

* FDR P-value < 0.05; AD: cases with current AD use; nAD: cases not currently taking AD

**Table S24. Secondary analyses for mean cortical thickness regions: AD versus nAD group comparison controlling for age, sex and remission status.**

|  | Cohen’s d (AD-nAD) | Std. Err. | 95 % CI | P-value | P_FDR_ | No. of AD | No. of nAD |
| --- | --- | --- | --- | --- | --- | --- | --- |
| Fusiform gyrus | -0.064 | 0.034 | -0.131 - 0.003 | 0.096 | 0.119 | 1828 | 943 |
| Inferior temporal gyrus | -0.100 | 0.035 | -0.168 - -0.032 | 0.011 | 0.042* | 1756 | 922 |
| Lateral orbitofrontal cortex | -0.051 | 0.034 | -0.118 - 0.016 | 0.183 | 0.203 | 1841 | 948 |
| Medial orbitofrontal cortex | -0.092 | 0.034 | -0.159 - -0.025 | 0.017 | 0.042* | 1837 | 939 |
| Parahippocampal gyrus | -0.068 | 0.034 | -0.135 - -0.001 | 0.077 | 0.119 | 1839 | 946 |
| Pars opercularis | -0.043 | 0.034 | -0.110 - 0.024 | 0.268 | 0.268 | 1830 | 944 |
| Superior temporal gyrus | -0.091 | 0.036 | -0.161 - -0.021 | 0.025 | 0.050* | 1604 | 872 |
| Frontal pole | -0.092 | 0.034 | -0.159 - -0.025 | 0.017 | 0.042* | 1840 | 949 |
| Insula | -0.065 | 0.034 | -0.133 - 0.002 | 0.093 | 0.119 | 1803 | 935 |

AD: cases with current AD use; nAD: cases not currently taking AD

**Table S25.** **Mega-analytic results for mean cortical surface area of the isthmus cingulate for the AD versus nAD group comparison controlling for age, sex and ICV.**

|  | Cohen’s d (AD-nAD) | Std. Err. | 95 % CI | P-value | P_FDR_ | No. of nAD | No. of AD |
| --- | --- | --- | --- | --- | --- | --- | --- |
| Isthmus cingulate cortex | 0.0344 | 0.0342 | -0.0327 - 0.1015 | 0.3162 | 0.3495 | 1472 | 2029 |

nAD: cases not currently taking AD; AD: cases with current AD use

**Table S26.** **Mega-analytic results for mean subcortical volume of the hippocampus for the AD versus HC group comparison controlling for age, sex and ICV.**

|  | Cohen’s d (AD-HC) | Std. Err. | 95 % CI | P-value | P_FDR_ | No. of HC | No. of AD |
| --- | --- | --- | --- | --- | --- | --- | --- |
| Hippocampus | -0.0996 | 0.0264 | -0.1513 - -0.0480 | 0.0002 | 0.0002* | 4984 | 2027 |

* FDR P-value < 0.05; HC: Healthy controls; AD: cases with current AD use

**Table S27.** **Mega-analytic results for mean subcortical volume of the hippocampus for the nAD versus HC group comparison controlling for age, sex and ICV.**

|  | Cohen’s d (nAD-HC) | Std. Err. | 95 % CI | P-value | P_FDR_ | No. of HC | No. of nAD |
| --- | --- | --- | --- | --- | --- | --- | --- |
| Hippocampus | -0.0093 | 0.0298 | -0.0490 – 0.0677 | 0.7540 | 0.7959 | 4984 | 1458 |

HC: Healthy controls; nAD: cases not currently taking AD

**Table S28.** **Mega-analytic results for mean cortical thickness regions for the AD versus HC group comparison controlling for age and sex.**

|  | Cohen’s d (AD-HC) | Std. Err. | 95 % CI | P-value | P_FDR_ | No. of HC | No. of AD |
| --- | --- | --- | --- | --- | --- | --- | --- |
| Banks superior temporal sulcus | -0.1035 | 0.0276 | -0.1575 - -0.0494 | 0.0002 | 0.0002* | 4738 | 1822 |
| Caudal anterior cingulate cortex | -0.0929 | 0.0263 | -0.1444 - -0.0413 | 0.0004 | 0.0005* | 5025 | 2033 |
| Entorhinal cortex | -0.0936 | 0.0275 | -0.1475 - -0.0398 | 0.0007 | 0.0007* | 4720 | 1847 |
| Fusiform gyrus | -0.1935 | 0.0263 | -0.2451 - -0.1419 | 0.0000 | 0.0000* | 5065 | 2025 |
| Inferior temporal gyrus | -0.1987 | 0.0268 | -0.2511 - -0.1462 | 0.0000 | 0.0000* | 4962 | 1950 |
| Isthmus cingulate cortex | -0.1285 | 0.0263 | -0.1800 - -0.0770 | 0.0000 | 0.0000* | 5048 | 2036 |
| Lateral orbitofrontal cortex | -0.1383 | 0.0263 | -0.1898 - -0.0868 | 0.0000 | 0.0000* | 5054 | 2038 |
| Medial orbitofrontal cortex | -0.1710 | 0.0264 | -0.2227 - -0.1193 | 0.0000 | 0.0000* | 4993 | 2028 |
| Parahippocampal gyrus | -0.1090 | 0.0262 | -0.1604 - -0.0575 | 0.0000 | 0.0001* | 5071 | 2037 |
| Pars opercularis | -0.1450 | 0.0263 | -0.1966 - -0.0934 | 0.0000 | 0.0000* | 5035 | 2029 |
| Posterior cingulate cortex | -0.1243 | 0.0262 | -0.1757- -0.0729 | 0.0000 | 0.0000* | 5053 | 2044 |
| Precuneus | -0.0734 | 0.0262 | -0.1248 - -0.0220 | 0.0052 | 0.0052* | 5041 | 2041 |
| Rostral anterior cingulate cortex | -0.1369 | 0.0263 | -0.1885 - -0.0852 | 0.0000 | 0.0000* | 4991 | 2031 |
| Superior temporal gyrus | -0.1526 | 0.0277 | -0.2070 - -0.0982 | 0.0000 | 0.0000* | 4705 | 1800 |
| Frontal pole | -0.0926 | 0.0262 | -0.1440 - -0.0411 | 0.0004 | 0.0005* | 5060 | 2038 |
| Transverse temporal gyrus | -0.1081 | 0.0262 | -0.1595 - -0.0567 | 0.0000 | 0.0001* | 5052 | 2043 |
| Insula | -0.2173 | 0.0266 | -0.2694 - -0.1651 | 0.0000 | 0.0000* | 4881 | 1999 |

* FDR P-value < .05; HC: Healthy controls; AD: cases with current AD use

**Table S29.** **Mega-analytic results for mean cortical thickness regions for the nAD versus HC group comparison controlling for age and sex.**

|  | Cohen’s d (nAD-HC) | Std. Err. | 95 % CI | P-value | P_FDR_ | No. of HC | No. of nAD |
| --- | --- | --- | --- | --- | --- | --- | --- |
| Banks superior temporal sulcus | -0.0727 | 0.0304 | -0.1322 - -0.0132 | 0.0167 | 0.0415* | 4738 | 1406 |
| Caudal anterior cingulate cortex | -0.0372 | 0.0296 | -0.0951 - 0.0207 | 0.2085 | 0.3015 | 5025 | 1483 |
| Entorhinal cortex | -0.0343 | 0.0304 | -0.0939 - 0.0254 | 0.2603 | 0.3297 | 4720 | 1401 |
| Fusiform gyrus | -0.1167 | 0.0296 | -0.1747 - -0.0586 | 0.0001 | 0.0016* | 5065 | 1473 |
| Inferior temporal gyrus | -0.0822 | 0.0298 | -0.1407 - -0.0238 | 0.0059 | 0.0273* | 4962 | 1453 |
| Isthmus cingulate cortex | -0.0646 | 0.0296 | -0.1226 - -0.0066 | 0.0291 | 0.0553 | 5048 | 1477 |
| Lateral orbitofrontal cortex | -0.0703 | 0.0296 | -0.1282 - -0.0123 | 0.0175 | 0.0415* | 5054 | 1481 |
| Medial orbitofrontal cortex | -0.0667 | 0.0297 | -0.1249 - -0.0084 | 0.0249 | 0.0526 | 4993 | 1466 |
| Parahippocampal gyrus | -0.0057 | 0.0296 | -0.0636 - 0.0523 | 0.8481 | 0.8481 | 5071 | 1477 |
| Pars opercularis | -0.0779 | 0.0296 | -0.1360 - -0.0198 | 0.0086 | 0.0273* | 5035 | 1471 |
| Posterior cingulate cortex | -0.0778 | 0.0296 | -0.1357 - -0.0199 | 0.0085 | 0.0273* | 5053 | 1481 |
| Precuneus | -0.0479 | 0.0296 | -0.1059 - 0.0101 | 0.1059 | 0.1677 | 5041 | 1476 |
| Rostral anterior cingulate cortex | -0.0970 | 0.0297 | -0.1551 - -0.0388 | 0.0011 | 0.0095* | 4991 | 1471 |
| Superior temporal gyrus | -0.0322 | 0.0305 | -0.0919 - 0.0276 | 0.2917 | 0.3464 | 4705 | 1395 |
| Frontal pole | 0.0251 | 0.0296 | -0.0328 - 0.0831 | 0.3953 | 0.4419 | 5060 | 1477 |
| Transverse temporal gyrus | -0.0606 | 0.0296 | -0.1186 - -0.0027 | 0.0405 | 0.0699 | 5052 | 1477 |
| Insula | -0.0946 | 0.0298 | -0.1531 - -0.0362 | 0.0015 | 0.0095* | 4881 | 1464 |

* FDR P-value < .05; HC: Healthy controls; nAD: cases not currently taking AD

**Table S30.** **Mega-analytic results for mean cortical surface area of the isthmus cingulate for the AD versus HC group comparison controlling for age, sex and ICV.**

|  | Cohen’s d (AD-HC) | Std. Err. | 95 % CI | P-value | P_FDR_ | No. of HC | No. of AD |
| --- | --- | --- | --- | --- | --- | --- | --- |
| Isthmus cingulate cortex | 0.0807 | 0.0263 | 0.0291 - 0.1322 | 0.0022 | 0.0023* | 5028 | 2029 |

* FDR P-value < .05; HC: Healthy controls; AD: cases with current AD use

**Table S31.** **Mega-analytic results for mean cortical surface area of the isthmus cingulate for the nAD versus HC group comparison controlling for age, sex and ICV.**

|  | Cohen’s d (nAD-HC) | Std. Err. | 95 % CI | P-value | P_FDR_ | No. of HC | No. of nAD |
| --- | --- | --- | --- | --- | --- | --- | --- |
| Isthmus cingulate cortex | 0.0362 | 0.0296 | -0.0219 - 0.0943 | 0.2221 | 0.3015 | 5028 | 1472 |

HC: Healthy controls; nAD: cases not currently taking AD

**Table S32. Secondary analyses for mean subcortical volume regions: 1) AD type*Age interaction, 2) AD type*Sex interaction and 3) main effect of AD type within the sample of the AD group controlling for age, sex and ICV.**

|  | **AD type*Age interaction** | | | | **AD type*Sex interaction** | | | | **AD type effect** | | | | **Number of subjects** | | |
| --- | --- | --- | --- | --- | --- | --- | --- | --- | --- | --- | --- | --- | --- | --- | --- |
|  | F | Partial η^2^ | P-value | P_FDR_ | F | Partial η^2^ | P-value | P_FDR_ | F | Partial η^2^ | P-value | P_FDR_ | SNRI | SSRI | Mirta- zapine |
| Lateral Ventricles | 0.7833 | 0.0018 | 0.4572 | 0.7588 | 1.8253 | 0.0042 | 0.1618 | 0.7657 | 1.4699 | 0.0033 | 0.2305 | 0.8290 | 373 | 393 | 120 |
| Thalamus | 0.2287 | 0.0005 | 0.7956 | 0.9008 | 3.0914 | 0.0070 | 0.0459 | 0.7657 | 2.3155 | 0.0052 | 0.0993 | 0.7306 | 372 | 395 | 120 |
| Caudate | 2.7884 | 0.0064 | 0.0621 | 0.6900 | 1.9035 | 0.0044 | 0.1497 | 0.7657 | 0.6498 | 0.0015 | 0.5224 | 0.8758 | 374 | 390 | 117 |
| Putamen | 1.9173 | 0.0045 | 0.1476 | 0.6900 | 0.8682 | 0.0020 | 0.4201 | 0.8028 | 0.7249 | 0.0017 | 0.4847 | 0.8758 | 369 | 379 | 116 |
| Pallidum | 0.0564 | 0.0001 | 0.9452 | 0.9647 | 0.3087 | 0.0007 | 0.7345 | 0.9222 | 0.1730 | 0.0004 | 0.8412 | 0.9282 | 365 | 382 | 115 |
| Hippocampus | 0.9913 | 0.0023 | 0.3715 | 0.6900 | 1.3875 | 0.0032 | 0.2502 | 0.7657 | 1.8470 | 0.0042 | 0.1583 | 0.7693 | 374 | 393 | 119 |
| Amygdala | 1.2652 | 0.0029 | 0.2827 | 0.6900 | 1.5328 | 0.0035 | 0.2165 | 0.7657 | 0.7302 | 0.0017 | 0.4821 | 0.8758 | 369 | 393 | 117 |
| Accumbens | 4.6167 | 0.0105 | 0.0101 | 0.2633 | 0.4105 | 0.0009 | 0.6635 | 0.8922 | 0.9780 | 0.0022 | 0.3765 | 0.8290 | 377 | 394 | 116 |

SNRI: selective serotonin and noradrenalin reuptake inhibitor; SSRI: selective serotonin reuptake inhibitor

**Table S33. Secondary analyses for mean cortical thickness regions: 1) AD type*Age interaction, 2) AD type*Sex interaction and 3) main effect of AD type within the sample of the AD group controlling for age and sex.**

|  |  | **AD type*Age interaction** | | |  | **AD type*Sex interaction** | | |  | **AD type effect** | | | **Number of subjects** | | |
| --- | --- | --- | --- | --- | --- | --- | --- | --- | --- | --- | --- | --- | --- | --- | --- |
|  | F | Partial η^2^ | P-value | P_FDR_ | F | Partial η^2^ | P-value | P_FDR_ | F | Partial η^2^ | P-value | P_FDR_ | SNRI | SSRI | Mirta- zapine |
| Banks superior temporal sulcus | 5.7524 | 0.0144 | 0.0033 | 0.1291 | 0.7411 | 0.0019 | 0.4769 | 0.8239 | 0.9995 | 0.0025 | 0.3685 | 0.8290 | 333 | 361 | 104 |
| Caudal anterior cingulate cortex | 2.6785 | 0.0061 | 0.0692 | 0.6900 | 0.6075 | 0.0014 | 0.5449 | 0.8428 | 1.2158 | 0.0027 | 0.2970 | 0.8290 | 372 | 399 | 118 |
| Caudal middle  frontal gyrus | 0.2793 | 0.0006 | 0.7564 | 0.8939 | 1.1756 | 0.0027 | 0.3091 | 0.8028 | 2.7301 | 0.0061 | 0.0658 | 0.7306 | 377 | 399 | 117 |
| Cuneus | 0.0563 | 0.0001 | 0.9452 | 0.9647 | 1.3678 | 0.0031 | 0.2552 | 0.7657 | 0.4743 | 0.0011 | 0.6225 | 0.8941 | 367 | 391 | 117 |
| Entorhinal cortex | 1.8240 | 0.0047 | 0.1621 | 0.6900 | 0.0033 | 0.0000 | 0.9967 | 0.9967 | 0.1302 | 0.0003 | 0.8779 | 0.9282 | 338 | 341 | 109 |
| Fusiform gyrus | 2.1872 | 0.0050 | 0.1128 | 0.6900 | 1.7979 | 0.0041 | 0.1663 | 0.7657 | 1.5642 | 0.0035 | 0.2098 | 0.8290 | 370 | 397 | 118 |
| Inferior parietal  cortex | 0.7049 | 0.0016 | 0.4944 | 0.7811 | 0.9530 | 0.0022 | 0.3860 | 0.8028 | 0.3540 | 0.0008 | 0.7019 | 0.9282 | 368 | 391 | 116 |
| Inferior temporal gyrus | 1.2003 | 0.0029 | 0.3016 | 0.6900 | 0.2073 | 0.0005 | 0.8128 | 0.9222 | 1.1498 | 0.0028 | 0.3172 | 0.8290 | 347 | 373 | 111 |
| Isthmus cingulate cortex | 0.2245 | 0.0005 | 0.7989 | 0.9008 | 1.4558 | 0.0033 | 0.2338 | 0.7657 | 2.8703 | 0.0064 | 0.0572 | 0.7306 | 371 | 402 | 117 |
| Lateral occipital cortex | 0.5143 | 0.0012 | 0.5981 | 0.7907 | 5.2304 | 0.0118 | 0.0055 | 0.4305 | 0.1605 | 0.0004 | 0.8518 | 0.9282 | 375 | 395 | 117 |
| Lateral orbitofrontal cortex | 0.0472 | 0.0001 | 0.9539 | 0.9647 | 1.4109 | 0.0032 | 0.2445 | 0.7657 | 0.6360 | 0.0014 | 0.5296 | 0.8758 | 377 | 398 | 118 |
| Lingual gyrus | 0.0359 | 0.0001 | 0.9647 | 0.9647 | 0.8470 | 0.0019 | 0.4290 | 0.8028 | 1.3581 | 0.0031 | 0.2577 | 0.8290 | 370 | 400 | 118 |
| Medial orbitofrontal cortex | 2.0214 | 0.0046 | 0.1331 | 0.6900 | 1.6607 | 0.0038 | 0.1906 | 0.7657 | 4.1857 | 0.0094 | 0.0155 | 0.5973 | 376 | 396 | 118 |
| Middle temporal gyrus | 1.6533 | 0.0041 | 0.1921 | 0.6900 | 0.5474 | 0.0013 | 0.5787 | 0.8428 | 1.6479 | 0.0040 | 0.1931 | 0.8260 | 340 | 372 | 110 |
| Parahippocampal gyrus | 1.1603 | 0.0026 | 0.3139 | 0.6900 | 0.1834 | 0.0004 | 0.8324 | 0.9222 | 1.1056 | 0.0025 | 0.3315 | 0.8290 | 377 | 396 | 118 |
| Paracentral lobule | 0.4051 | 0.0009 | 0.6670 | 0.8529 | 2.2928 | 0.0051 | 0.1016 | 0.7657 | 2.4788 | 0.0055 | 0.0844 | 0.7306 | 379 | 403 | 117 |
| Pars opercularis | 2.3045 | 0.0052 | 0.1004 | 0.6900 | 0.2719 | 0.0006 | 0.7620 | 0.9222 | 2.9853 | 0.0067 | 0.0510 | 0.7306 | 375 | 399 | 119 |
| Pars orbitalis | 1.0852 | 0.0025 | 0.3383 | 0.6900 | 0.1751 | 0.0004 | 0.8394 | 0.9222 | 1.7364 | 0.0039 | 0.1768 | 0.8006 | 375 | 396 | 120 |
| Pars triangularis | 1.7725 | 0.0040 | 0.1705 | 0.6900 | 0.9006 | 0.0020 | 0.4067 | 0.8028 | 0.9259 | 0.0021 | 0.3965 | 0.8290 | 375 | 403 | 118 |
| Pericalcarine cortex | 0.7599 | 0.0018 | 0.4680 | 0.7605 | 0.0990 | 0.0002 | 0.9057 | 0.9326 | 0.6687 | 0.0015 | 0.5126 | 0.8758 | 367 | 388 | 113 |
| Postcentral gyrus | 1.3096 | 0.0030 | 0.2704 | 0.6900 | 2.1475 | 0.0049 | 0.1174 | 0.7657 | 0.9214 | 0.0021 | 0.3983 | 0.8290 | 375 | 399 | 115 |
| Posterior cingulate cortex | 0.6627 | 0.0015 | 0.5157 | 0.7811 | 1.6371 | 0.0037 | 0.1951 | 0.7657 | 1.0827 | 0.0024 | 0.3391 | 0.8290 | 380 | 398 | 118 |
| Precentral gyrus | 1.8300 | 0.0041 | 0.1610 | 0.6900 | 0.6582 | 0.0015 | 0.5181 | 0.8247 | 1.4110 | 0.0032 | 0.2445 | 0.8290 | 377 | 397 | 116 |
| Precuneus | 0.6685 | 0.0015 | 0.5128 | 0.7811 | 0.2092 | 0.0005 | 0.8113 | 0.9222 | 0.7219 | 0.0016 | 0.4861 | 0.8758 | 373 | 402 | 120 |
| Rostral anterior cingulate cortex | 8.1618 | 0.0183 | 0.0003 | 0.0240* | 0.8817 | 0.0020 | 0.4145 | 0.8028 | - | - | - | - | 369 | 398 | 119 |
| Rostral middle frontal gyrus | 0.3058 | 0.0007 | 0.7366 | 0.8939 | 0.1093 | 0.0002 | 0.8965 | 0.9326 | 3.2367 | 0.0073 | 0.0398 | 0.7306 | 373 | 397 | 116 |
| Superior frontal gyrus | 0.9412 | 0.0021 | 0.3906 | 0.7003 | 0.7737 | 0.0018 | 0.4616 | 0.8183 | 1.2064 | 0.0027 | 0.2998 | 0.8290 | 378 | 397 | 116 |
| Superior parietal cortex | 0.1400 | 0.0003 | 0.8694 | 0.9418 | 0.1151 | 0.0003 | 0.8913 | 0.9326 | 0.2067 | 0.0005 | 0.8133 | 0.9282 | 375 | 401 | 119 |
| Superior temporal gyrus | 1.2578 | 0.0033 | 0.2849 | 0.6900 | 0.9370 | 0.0024 | 0.3923 | 0.8028 | 1.3415 | 0.0035 | 0.2621 | 0.8290 | 327 | 351 | 101 |
| Supramarginal gyrus | 0.8949 | 0.0022 | 0.4090 | 0.7090 | 1.5434 | 0.0037 | 0.2143 | 0.7657 | 0.9853 | 0.0024 | 0.3738 | 0.8290 | 354 | 375 | 107 |
| Frontal pole | 1.1999 | 0.0027 | 0.3017 | 0.6900 | 0.2255 | 0.0005 | 0.7981 | 0.9222 | 0.3071 | 0.0007 | 0.7356 | 0.9282 | 377 | 398 | 120 |
| Temporal pole | 1.3861 | 0.0032 | 0.2506 | 0.6900 | 0.4695 | 0.0011 | 0.6255 | 0.8712 | 0.2874 | 0.0007 | 0.7503 | 0.9282 | 372 | 397 | 117 |
| Transverse temporal gyrus | 0.2127 | 0.0005 | 0.8084 | 0.9008 | 0.6791 | 0.0015 | 0.5073 | 0.8247 | 2.5645 | 0.0057 | 0.0775 | 0.7306 | 379 | 399 | 119 |
| Insula | 0.2899 | 0.0007 | 0.7484 | 0.8939 | 0.4471 | 0.0010 | 0.6396 | 0.8753 | 4.5288 | 0.0104 | 0.0111 | 0.5973 | 362 | 394 | 110 |
| Average thickness | 1.4186 | 0.0032 | 0.2426 | 0.6900 | 1.1807 | 0.0026 | 0.3076 | 0.8028 | 2.1140 | 0.0047 | 0.1214 | 0.7306 | 380 | 402 | 120 |
|  |  |  |  |  |  |  |  |  |  |  |  |  |  |  |  |

* FDR P-value < 0.05; SNRI: selective serotonin and noradrenalin reuptake inhibitor; SSRI: selective serotonin reuptake inhibitor

**Table S34.** **Secondary analyses for mean cortical surface area regions: 1) AD type*Age interaction, 2) AD type*Sex interaction and 3) main effect of AD type within the sample of the AD group controlling for age, sex and ICV.**

|  |  | **AD type*Age interaction** | | |  | **AD type*Sex interaction** | | |  | **AD type effect** | | | **Number of subjects** | | |
| --- | --- | --- | --- | --- | --- | --- | --- | --- | --- | --- | --- | --- | --- | --- | --- |
|  | F | Partial η^2^ | P-value | P_FDR_ | F | Partial η^2^ | P-value | P_FDR_ | F | Partial η^2^ | P-value | P_FDR_ | SNRI | SSRI | Mirta- zapine |
| Banks superior temporal sulcus | 0.5437 | 0.0014 | 0.5808 | 0.7811 | 0.5476 | 0.0014 | 0.5785 | 0.8428 | 0.5960 | 0.0015 | 0.5513 | 0.8758 | 331 | 359 | 103 |
| Caudal anterior cingulate cortex | 0.5439 | 0.0012 | 0.5807 | 0.7811 | 0.1931 | 0.0004 | 0.8245 | 0.9222 | 0.5128 | 0.0012 | 0.5990 | 0.8941 | 371 | 398 | 115 |
| Caudal middle  frontal gyrus | 0.0421 | 0.0001 | 0.9588 | 0.9647 | 0.7915 | 0.0018 | 0.4535 | 0.8183 | 0.2724 | 0.0006 | 0.7616 | 0.9282 | 376 | 397 | 116 |
| Cuneus | 0.5873 | 0.0014 | 0.5561 | 0.7811 | 2.4239 | 0.0056 | 0.0892 | 0.7657 | 0.0486 | 0.0001 | 0.9525 | 0.9651 | 367 | 392 | 117 |
| Entorhinal cortex | 2.3541 | 0.0060 | 0.0957 | 0.6900 | 1.7205 | 0.0044 | 0.1797 | 0.7657 | 0.3442 | 0.0009 | 0.7089 | 0.9282 | 339 | 342 | 108 |
| Fusiform gyrus | 1.8408 | 0.0042 | 0.1593 | 0.6900 | 0.2829 | 0.0006 | 0.7536 | 0.9222 | 0.5850 | 0.0013 | 0.5573 | 0.8758 | 369 | 396 | 118 |
| Inferior parietal  cortex | 1.1435 | 0.0026 | 0.3192 | 0.6900 | 0.3503 | 0.0008 | 0.7045 | 0.9159 | 2.0234 | 0.0046 | 0.1328 | 0.7306 | 366 | 393 | 116 |
| Inferior temporal gyrus | 1.0588 | 0.0026 | 0.3474 | 0.6900 | 0.8606 | 0.0021 | 0.4233 | 0.8028 | 0.1917 | 0.0005 | 0.8256 | 0.9282 | 348 | 373 | 109 |
| Isthmus cingulate cortex | 1.1337 | 0.0026 | 0.3223 | 0.6900 | 2.0323 | 0.0046 | 0.1316 | 0.7657 | 0.4986 | 0.0011 | 0.6076 | 0.8941 | 370 | 402 | 117 |
| Lateral occipital cortex | 0.0450 | 0.0001 | 0.9560 | 0.9647 | 1.1391 | 0.0026 | 0.3206 | 0.8028 | 1.8374 | 0.0042 | 0.1598 | 0.7693 | 374 | 396 | 115 |
| Lateral orbitofrontal cortex | 1.1695 | 0.0026 | 0.3110 | 0.6900 | 0.2381 | 0.0005 | 0.7882 | 0.9222 | 0.2517 | 0.0006 | 0.7775 | 0.9282 | 376 | 398 | 119 |
| Lingual gyrus | 0.1737 | 0.0004 | 0.8406 | 0.9234 | 3.1823 | 0.0072 | 0.0420 | 0.7657 | 0.5421 | 0.0012 | 0.5817 | 0.8941 | 369 | 400 | 118 |
| Medial orbitofrontal cortex | 0.3013 | 0.0007 | 0.7399 | 0.8939 | 0.3861 | 0.0009 | 0.6798 | 0.8988 | 0.1373 | 0.0003 | 0.8717 | 0.9282 | 374 | 398 | 117 |
| Middle temporal gyrus | 0.2581 | 0.0006 | 0.7726 | 0.8994 | 0.9827 | 0.0024 | 0.3747 | 0.8028 | 0.9662 | 0.0024 | 0.3810 | 0.8290 | 341 | 371 | 110 |
| Parahippocampal gyrus | 1.8688 | 0.0042 | 0.1549 | 0.6900 | 1.4220 | 0.0032 | 0.2418 | 0.7657 | 0.0944 | 0.0002 | 0.9099 | 0.9342 | 374 | 395 | 118 |
| Paracentral lobule | 0.5832 | 0.0013 | 0.5583 | 0.7811 | 0.0827 | 0.0002 | 0.9206 | 0.9326 | 2.0765 | 0.0046 | 0.1260 | 0.7306 | 377 | 401 | 120 |
| Pars opercularis | 1.5539 | 0.0035 | 0.2120 | 0.6900 | 0.5809 | 0.0013 | 0.5596 | 0.8428 | 0.2764 | 0.0006 | 0.7586 | 0.9282 | 374 | 399 | 119 |
| Pars orbitalis | 1.1816 | 0.0027 | 0.3073 | 0.6900 | 0.6589 | 0.0015 | 0.5177 | 0.8247 | 0.0344 | 0.0001 | 0.9661 | 0.9661 | 374 | 399 | 119 |
| Pars triangularis | 1.0841 | 0.0025 | 0.3386 | 0.6900 | 0.7223 | 0.0016 | 0.4859 | 0.8239 | 0.4547 | 0.0010 | 0.6348 | 0.8941 | 373 | 402 | 119 |
| Pericalcarine cortex | 0.8675 | 0.0020 | 0.4204 | 0.7128 | 1.0644 | 0.0025 | 0.3454 | 0.8028 | 0.1705 | 0.0004 | 0.8433 | 0.9282 | 369 | 389 | 113 |
| Postcentral gyrus | 2.4638 | 0.0056 | 0.0857 | 0.6900 | 3.9980 | 0.0091 | 0.0187 | 0.7288 | 2.1708 | 0.0049 | 0.1147 | 0.7306 | 375 | 398 | 113 |
| Posterior cingulate cortex | 1.7971 | 0.0041 | 0.1664 | 0.6900 | 1.9099 | 0.0043 | 0.1487 | 0.7657 | 0.1002 | 0.0002 | 0.9046 | 0.9342 | 380 | 396 | 118 |
| Precentral gyrus | 1.6540 | 0.0038 | 0.1919 | 0.6900 | 0.0840 | 0.0002 | 0.9194 | 0.9326 | 2.3421 | 0.0053 | 0.0967 | 0.7306 | 376 | 396 | 117 |
| Precuneus | 0.4457 | 0.0010 | 0.6405 | 0.8326 | 0.2936 | 0.0007 | 0.7457 | 0.9222 | 0.4486 | 0.0010 | 0.6387 | 0.8941 | 372 | 401 | 120 |
| Rostral anterior cingulate cortex | 1.4056 | 0.0032 | 0.2458 | 0.6900 | 0.5390 | 0.0012 | 0.5835 | 0.8428 | 0.3170 | 0.0007 | 0.7284 | 0.9282 | 369 | 395 | 119 |
| Rostral middle frontal gyrus | 0.5441 | 0.0012 | 0.5806 | 0.7811 | 1.2927 | 0.0029 | 0.2750 | 0.7946 | 0.4032 | 0.0009 | 0.6683 | 0.9189 | 374 | 396 | 114 |
| Superior frontal gyrus | 1.2391 | 0.0028 | 0.2902 | 0.6900 | 1.7661 | 0.0040 | 0.1716 | 0.7657 | 1.3992 | 0.0032 | 0.2474 | 0.8290 | 376 | 397 | 115 |
| Superior parietal cortex | 1.0232 | 0.0023 | 0.3599 | 0.6900 | 1.8730 | 0.0042 | 0.1543 | 0.7657 | 0.8342 | 0.0019 | 0.4345 | 0.8580 | 374 | 400 | 119 |
| Superior temporal gyrus | 1.0061 | 0.0026 | 0.3661 | 0.6900 | 0.8397 | 0.0022 | 0.4323 | 0.8028 | 0.5878 | 0.0015 | 0.5558 | 0.8758 | 326 | 352 | 101 |
| Supramarginal gyrus | 0.3378 | 0.0008 | 0.7134 | 0.8939 | 0.5115 | 0.0012 | 0.5998 | 0.8506 | 0.1279 | 0.0003 | 0.8800 | 0.9282 | 354 | 375 | 107 |
| Frontal pole | 1.4145 | 0.0032 | 0.2436 | 0.6900 | 1.4114 | 0.0032 | 0.2443 | 0.7657 | 0.1923 | 0.0004 | 0.8251 | 0.9282 | 377 | 403 | 120 |
| Temporal pole | 0.6059 | 0.0014 | 0.5458 | 0.7811 | 2.1607 | 0.0049 | 0.1159 | 0.7657 | 0.8788 | 0.0020 | 0.4156 | 0.8422 | 373 | 399 | 120 |
| Transverse temporal gyrus | 1.3952 | 0.0031 | 0.2483 | 0.6900 | 0.1397 | 0.0003 | 0.8696 | 0.9326 | 1.0141 | 0.0023 | 0.3631 | 0.8290 | 378 | 400 | 120 |
| Insula | 0.9298 | 0.0022 | 0.3950 | 0.7003 | 1.1449 | 0.0027 | 0.3187 | 0.8028 | 1.2958 | 0.0030 | 0.2742 | 0.8290 | 359 | 395 | 110 |
| Total surface area | 0.6173 | 0.0014 | 0.5396 | 0.7811 | 0.8935 | 0.0020 | 0.4096 | 0.8028 | 0.5868 | 0.0013 | 0.5563 | 0.8758 | 377 | 403 | 118 |
|  |  |  |  |  |  |  |  |  |  |  |  |  |  |  |  |

SNRI: selective serotonin and noradrenalin reuptake inhibitor; SSRI: selective serotonin reuptake inhibitor

**Table S35.** **Secondary analyses for mean subcortical volume regions: 1) AD duration*Age interaction, 2) AD duration*Sex interaction and 3) main effect of AD duration within the sample of the AD group controlling for age, sex and ICV.**

|  | **AD duration*Age interaction** | | | | **AD duration*Sex interaction** | | | | **AD duration effect** | | | | **Number of subjects** | | |
| --- | --- | --- | --- | --- | --- | --- | --- | --- | --- | --- | --- | --- | --- | --- | --- |
|  | F | Partial η^2^ | P-value | P_FDR_ | F | Partial η^2^ | P-value | P_FDR_ | F | Partial η^2^ | P-value | P_FDR_ |  | AD |  |
| Lateral Ventricles | 1.2850 | 0.0045 | 0.2579 | 0.7699 | 2.3787 | 0.0082 | 0.1241 | 0.8598 | 0.0461 | 0.0002 | 0.8302 | 0.9462 |  | 295 |  |
| Thalamus | 0.1081 | 0.0004 | 0.7426 | 0.8911 | 0.8737 | 0.0030 | 0.3507 | 0.8598 | 0.5334 | 0.0018 | 0.4658 | 0.9062 |  | 297 |  |
| Caudate | 0.8872 | 0.0031 | 0.3470 | 0.7699 | 1.8898 | 0.0067 | 0.1703 | 0.8598 | 1.1206 | 0.0039 | 0.2907 | 0.9062 |  | 290 |  |
| Putamen | 4.1121 | 0.0151 | 0.0436 | 0.4594 | 3.0363 | 0.0112 | 0.0826 | 0.8598 | 0.9905 | 0.0037 | 0.3205 | 0.9062 |  | 276 |  |
| Pallidum | 0.0175 | 0.0001 | 0.8950 | 0.9434 | 0.0020 | 0.0000 | 0.9642 | 0.9861 | 0.3790 | 0.0014 | 0.5387 | 0.9062 |  | 271 |  |
| Hippocampus | 1.1635 | 0.0040 | 0.2816 | 0.7699 | 0.1341 | 0.0005 | 0.7145 | 0.9601 | 1.2790 | 0.0044 | 0.2590 | 0.8784 |  | 296 |  |
| Amygdala | 4.1052 | 0.0142 | 0.0437 | 0.4594 | 0.2739 | 0.0010 | 0.6011 | 0.9194 | 0.0073 | 0.0000 | 0.9321 | 0.9759 |  | 293 |  |
| Accumbens | 0.0449 | 0.0002 | 0.8323 | 0.9300 | 0.3427 | 0.0012 | 0.5587 | 0.9079 | 5.0152 | 0.0171 | 0.0259 | 0.4557 |  | 296 |  |

AD: cases with current AD use

**Table S36.** **Secondary analyses for mean cortical thickness regions: 1) AD duration*Age interaction, 2) AD duration*Sex interaction and 3) main effect of AD duration within the sample of the AD group controlling for age and sex.**

|  |  | **AD duration*Age interaction** | | |  | **AD duration*Sex interaction** | | |  | **AD duration effect** | | | **Number of subjects** | | |
| --- | --- | --- | --- | --- | --- | --- | --- | --- | --- | --- | --- | --- | --- | --- | --- |
|  | F | Partial η^2^ | P-value | P_FDR_ | F | Partial η^2^ | P-value | P_FDR_ | F | Partial η^2^ | P-value | P_FDR_ |  | AD |  |
| Banks superior temporal sulcus | 0.8950 | 0.0034 | 0.3450 | 0.7699 | 0.0605 | 0.0002 | 0.8059 | 0.9770 | 0.4861 | 0.0018 | 0.4863 | 0.9062 |  | 271 |  |
| Caudal anterior cingulate cortex | 0.1740 | 0.0006 | 0.6769 | 0.8515 | 0.6519 | 0.0022 | 0.4201 | 0.8598 | 1.4656 | 0.0050 | 0.2270 | 0.8784 |  | 298 |  |
| Caudal middle  frontal gyrus | 3.6552 | 0.0123 | 0.0569 | 0.4594 | 0.1476 | 0.0005 | 0.7012 | 0.9601 | 0.1092 | 0.0004 | 0.7413 | 0.9062 |  | 300 |  |
| Cuneus | 0.6021 | 0.0021 | 0.4384 | 0.7851 | 0.0019 | 0.0000 | 0.9656 | 0.9861 | 0.0175 | 0.0001 | 0.8947 | 0.9693 |  | 291 |  |
| Entorhinal cortex | 0.7063 | 0.0029 | 0.4015 | 0.7851 | 0.1421 | 0.0006 | 0.7065 | 0.9601 | 0.1901 | 0.0008 | 0.6632 | 0.9062 |  | 253 |  |
| Fusiform gyrus | 1.5993 | 0.0054 | 0.2070 | 0.7689 | 0.7262 | 0.0025 | 0.3948 | 0.8598 | 0.4657 | 0.0016 | 0.4955 | 0.9062 |  | 300 |  |
| Inferior parietal  cortex | 3.3165 | 0.0115 | 0.0696 | 0.4594 | 0.2971 | 0.0010 | 0.5861 | 0.9194 | 0.8292 | 0.0029 | 0.3633 | 0.9062 |  | 291 |  |
| Inferior temporal gyrus | 2.0644 | 0.0073 | 0.1519 | 0.6499 | 0.2398 | 0.0008 | 0.6247 | 0.9232 | 0.1285 | 0.0005 | 0.7203 | 0.9062 |  | 288 |  |
| Isthmus cingulate cortex | 0.6347 | 0.0022 | 0.4263 | 0.7851 | 0.0735 | 0.0003 | 0.7865 | 0.9770 | 0.1073 | 0.0004 | 0.7435 | 0.9062 |  | 299 |  |
| Lateral occipital cortex | 0.0097 | 0.0000 | 0.9215 | 0.9584 | 1.1000 | 0.0037 | 0.2951 | 0.8598 | 2.6235 | 0.0088 | 0.1064 | 0.5926 |  | 299 |  |
| Lateral orbitofrontal cortex | 1.4517 | 0.0049 | 0.2292 | 0.7699 | 2.1878 | 0.0074 | 0.1402 | 0.8598 | 0.1162 | 0.0004 | 0.7335 | 0.9062 |  | 301 |  |
| Lingual gyrus | 0.0399 | 0.0001 | 0.8419 | 0.9300 | 0.0130 | 0.0000 | 0.9095 | 0.9770 | 2.9465 | 0.0100 | 0.0871 | 0.5926 |  | 295 |  |
| Medial orbitofrontal cortex | 0.4913 | 0.0017 | 0.4839 | 0.8031 | 1.3431 | 0.0046 | 0.2474 | 0.8598 | 0.3030 | 0.0010 | 0.5824 | 0.9062 |  | 298 |  |
| Middle temporal gyrus | 1.9230 | 0.0069 | 0.1666 | 0.6499 | 1.2691 | 0.0045 | 0.2609 | 0.8598 | 0.0401 | 0.0001 | 0.8414 | 0.9462 |  | 284 |  |
| Parahippocampal gyrus | 0.8707 | 0.0030 | 0.3515 | 0.7699 | 0.5714 | 0.0019 | 0.4503 | 0.8598 | 0.0062 | 0.0000 | 0.9371 | 0.9759 |  | 299 |  |
| Paracentral lobule | 0.2056 | 0.0007 | 0.6506 | 0.8489 | 0.1875 | 0.0006 | 0.6653 | 0.9601 | 0.0060 | 0.0000 | 0.9384 | 0.9759 |  | 303 |  |
| Pars opercularis | 3.2908 | 0.0110 | 0.0707 | 0.4594 | 0.2814 | 0.0010 | 0.5962 | 0.9194 | 0.0969 | 0.0003 | 0.7558 | 0.9069 |  | 301 |  |
| Pars orbitalis | 7.2971 | 0.0241 | 0.0073 | 0.3844 | 0.7228 | 0.0024 | 0.3959 | 0.8598 | 0.2819 | 0.0009 | 0.5958 | 0.9062 |  | 301 |  |
| Pars triangularis | 0.7938 | 0.0027 | 0.3737 | 0.7851 | 0.0427 | 0.0001 | 0.8365 | 0.9770 | 0.7982 | 0.0027 | 0.3724 | 0.9062 |  | 303 |  |
| Pericalcarine cortex | 0.5593 | 0.0020 | 0.4552 | 0.7851 | 0.0143 | 0.0001 | 0.9049 | 0.9770 | 0.5842 | 0.0021 | 0.4453 | 0.9062 |  | 282 |  |
| Postcentral gyrus | 1.4731 | 0.0051 | 0.2258 | 0.7699 | 0.7148 | 0.0025 | 0.3985 | 0.8598 | 0.2100 | 0.0007 | 0.6471 | 0.9062 |  | 296 |  |
| Posterior cingulate cortex | 0.3369 | 0.0011 | 0.5621 | 0.8489 | 0.9233 | 0.0031 | 0.3374 | 0.8598 | 4.4886 | 0.0149 | 0.0350 | 0.4557 |  | 301 |  |
| Precentral gyrus | 0.2183 | 0.0007 | 0.6407 | 0.8489 | 0.0114 | 0.0000 | 0.9149 | 0.9770 | 0.0707 | 0.0002 | 0.7904 | 0.9202 |  | 300 |  |
| Precuneus | 0.5812 | 0.0020 | 0.4465 | 0.7851 | 0.0086 | 0.0000 | 0.9260 | 0.9770 | 0.1276 | 0.0004 | 0.7212 | 0.9062 |  | 302 |  |
| Rostral anterior cingulate cortex | 3.3261 | 0.0114 | 0.0692 | 0.4594 | 0.0759 | 0.0003 | 0.7832 | 0.9770 | 0.5633 | 0.0019 | 0.4536 | 0.9062 |  | 294 |  |
| Rostral middle frontal gyrus | 2.0664 | 0.0069 | 0.1516 | 0.6499 | 1.7359 | 0.0058 | 0.1887 | 0.8598 | 0.5309 | 0.0018 | 0.4668 | 0.9062 |  | 302 |  |
| Superior frontal gyrus | 1.2317 | 0.0042 | 0.2680 | 0.7699 | 0.6889 | 0.0023 | 0.4072 | 0.8598 | 0.3110 | 0.0011 | 0.5775 | 0.9062 |  | 299 |  |
| Superior parietal cortex | 0.2087 | 0.0007 | 0.6481 | 0.8489 | 0.5437 | 0.0018 | 0.4615 | 0.8598 | 0.9964 | 0.0033 | 0.3190 | 0.9062 |  | 302 |  |
| Superior temporal gyrus | 2.5299 | 0.0098 | 0.1129 | 0.6293 | 0.4680 | 0.0018 | 0.4945 | 0.8598 | 0.0000 | 0.0000 | 0.9955 | 0.9955 |  | 261 |  |
| Supramarginal gyrus | 2.0498 | 0.0075 | 0.1534 | 0.6499 | 0.0240 | 0.0001 | 0.8771 | 0.9770 | 0.0333 | 0.0001 | 0.8554 | 0.9462 |  | 276 |  |
| Frontal pole | 0.3300 | 0.0011 | 0.5661 | 0.8489 | 2.4995 | 0.0084 | 0.1149 | 0.8598 | 6.0927 | 0.0200 | 0.0141 | 0.4557 |  | 302 |  |
| Temporal pole | 0.0427 | 0.0001 | 0.8365 | 0.9300 | 0.4906 | 0.0017 | 0.4842 | 0.8598 | 0.0306 | 0.0001 | 0.8613 | 0.9462 |  | 301 |  |
| Transverse temporal gyrus | 0.0044 | 0.0000 | 0.9474 | 0.9597 | 0.8842 | 0.0030 | 0.3478 | 0.8598 | 0.3385 | 0.0011 | 0.5611 | 0.9062 |  | 301 |  |
| Insula | 0.9809 | 0.0035 | 0.3228 | 0.7699 | 0.1396 | 0.0005 | 0.7089 | 0.9601 | 0.3315 | 0.0012 | 0.5652 | 0.9062 |  | 284 |  |
| Average thickness | 0.8571 | 0.0029 | 0.3553 | 0.7699 | 0.4631 | 0.0016 | 0.4967 | 0.8598 | 0.1789 | 0.0006 | 0.6726 | 0.9062 |  | 302 |  |
|  |  |  |  |  |  |  |  |  |  |  |  |  |  |  |  |

AD: cases with current AD use

**Table S37.** **Secondary analyses for mean cortical surface area regions: 1) AD duration*Age interaction, 2) AD duration*Sex interaction and 3) main effect of AD duration within the sample of the AD group controlling for age, sex and ICV.**

|  |  | **AD duration*Age interaction** | | |  | **AD duration*Sex interaction** | | |  | **AD duration effect** | | | **Number of subjects** | | |
| --- | --- | --- | --- | --- | --- | --- | --- | --- | --- | --- | --- | --- | --- | --- | --- |
|  | F | Partial η^2^ | P-value | P_FDR_ | F | Partial η^2^ | P-value | P_FDR_ | F | Partial η^2^ | P-value | P_FDR_ |  | AD |  |
| Banks superior temporal sulcus | 4.6836 | 0.0177 | 0.0314 | 0.4594 | 0.2363 | 0.0009 | 0.6273 | 0.9232 | 0.7186 | 0.0027 | 0.3974 | 0.9062 |  | 268 |  |
| Caudal anterior cingulate cortex | 0.2514 | 0.0009 | 0.6165 | 0.8489 | 1.5435 | 0.0053 | 0.2151 | 0.8598 | 0.0735 | 0.0003 | 0.7865 | 0.9202 |  | 296 |  |
| Caudal middle  frontal gyrus | 0.6371 | 0.0022 | 0.4254 | 0.7851 | 3.1413 | 0.0107 | 0.0774 | 0.8598 | 1.4176 | 0.0048 | 0.2348 | 0.8784 |  | 299 |  |
| Cuneus | 1.9247 | 0.0068 | 0.1664 | 0.6499 | 0.0580 | 0.0002 | 0.8098 | 0.9770 | 2.6614 | 0.0093 | 0.1039 | 0.5926 |  | 290 |  |
| Entorhinal cortex | 1.1232 | 0.0046 | 0.2903 | 0.7699 | 2.0213 | 0.0082 | 0.1564 | 0.8598 | 0.1432 | 0.0006 | 0.7054 | 0.9062 |  | 252 |  |
| Fusiform gyrus | 0.6424 | 0.0022 | 0.4235 | 0.7851 | 0.0005 | 0.0000 | 0.9826 | 0.9861 | 0.1546 | 0.0005 | 0.6945 | 0.9062 |  | 300 |  |
| Inferior parietal  cortex | 4.8869 | 0.0170 | 0.0279 | 0.4594 | 1.0303 | 0.0036 | 0.3110 | 0.8598 | 0.1748 | 0.0006 | 0.6762 | 0.9062 |  | 290 |  |
| Inferior temporal gyrus | 0.9021 | 0.0032 | 0.3430 | 0.7699 | 5.3869 | 0.0186 | 0.0210 | 0.7726 | 0.8813 | 0.0031 | 0.3486 | 0.9062 |  | 291 |  |
| Isthmus cingulate cortex | 3.4389 | 0.0117 | 0.0647 | 0.4594 | 1.0766 | 0.0037 | 0.3003 | 0.8598 | 0.4272 | 0.0015 | 0.5139 | 0.9062 |  | 299 |  |
| Lateral occipital cortex | 0.0023 | 0.0000 | 0.9622 | 0.9622 | 0.9699 | 0.0033 | 0.3255 | 0.8598 | 2.8578 | 0.0097 | 0.0920 | 0.5926 |  | 298 |  |
| Lateral orbitofrontal cortex | 0.0375 | 0.0001 | 0.8467 | 0.9300 | 0.0184 | 0.0001 | 0.8923 | 0.9770 | 0.0004 | 0.0000 | 0.9847 | 0.9955 |  | 300 |  |
| Lingual gyrus | 0.2987 | 0.0010 | 0.5851 | 0.8489 | 0.0084 | 0.0000 | 0.9269 | 0.9770 | 4.1039 | 0.0140 | 0.0437 | 0.4557 |  | 294 |  |
| Medial orbitofrontal cortex | 0.0318 | 0.0001 | 0.8585 | 0.9300 | 1.5264 | 0.0053 | 0.2177 | 0.8598 | 0.4781 | 0.0016 | 0.4898 | 0.9062 |  | 295 |  |
| Middle temporal gyrus | 0.4302 | 0.0016 | 0.5124 | 0.8157 | 0.4638 | 0.0017 | 0.4964 | 0.8598 | 4.6414 | 0.0164 | 0.0321 | 0.4557 |  | 283 |  |
| Parahippocampal gyrus | 1.1004 | 0.0038 | 0.2951 | 0.7699 | 0.5320 | 0.0018 | 0.4663 | 0.8598 | 0.0001 | 0.0000 | 0.9908 | 0.9955 |  | 297 |  |
| Paracentral lobule | 6.6184 | 0.0221 | 0.0106 | 0.3844 | 0.1040 | 0.0004 | 0.7473 | 0.9715 | 0.4021 | 0.0014 | 0.5265 | 0.9062 |  | 302 |  |
| Pars opercularis | 0.2450 | 0.0008 | 0.6210 | 0.8489 | 0.7602 | 0.0026 | 0.3840 | 0.8598 | 5.3526 | 0.0178 | 0.0214 | 0.4557 |  | 303 |  |
| Pars orbitalis | 6.0131 | 0.0201 | 0.0148 | 0.3844 | 2.3307 | 0.0079 | 0.1279 | 0.8598 | 0.1969 | 0.0007 | 0.6576 | 0.9062 |  | 300 |  |
| Pars triangularis | 0.0549 | 0.0002 | 0.8149 | 0.9300 | 0.4412 | 0.0015 | 0.5070 | 0.8598 | 0.2056 | 0.0007 | 0.6506 | 0.9062 |  | 302 |  |
| Pericalcarine cortex | 0.5401 | 0.0020 | 0.4630 | 0.7851 | 0.0003 | 0.0000 | 0.9861 | 0.9861 | 3.9901 | 0.0141 | 0.0467 | 0.4557 |  | 284 |  |
| Postcentral gyrus | 0.1456 | 0.0005 | 0.7031 | 0.8705 | 0.3997 | 0.0014 | 0.5278 | 0.8758 | 1.2852 | 0.0044 | 0.2579 | 0.8784 |  | 295 |  |
| Posterior cingulate cortex | 0.0199 | 0.0001 | 0.8878 | 0.9434 | 0.5714 | 0.0019 | 0.4503 | 0.8598 | 0.4926 | 0.0017 | 0.4833 | 0.9062 |  | 301 |  |
| Precentral gyrus | 0.7557 | 0.0026 | 0.3854 | 0.7851 | 1.4418 | 0.0049 | 0.2308 | 0.8598 | 1.3892 | 0.0047 | 0.2395 | 0.8784 |  | 298 |  |
| Precuneus | 1.2971 | 0.0044 | 0.2557 | 0.7699 | 2.3108 | 0.0078 | 0.1296 | 0.8598 | 1.9750 | 0.0067 | 0.1610 | 0.7847 |  | 301 |  |
| Rostral anterior cingulate cortex | 0.2317 | 0.0008 | 0.6306 | 0.8489 | 0.0111 | 0.0000 | 0.9162 | 0.9770 | 0.3440 | 0.0012 | 0.5580 | 0.9062 |  | 293 |  |
| Rostral middle frontal gyrus | 0.8831 | 0.0030 | 0.3481 | 0.7699 | 0.5338 | 0.0018 | 0.4656 | 0.8598 | 2.9270 | 0.0098 | 0.0882 | 0.5926 |  | 300 |  |
| Superior frontal gyrus | 0.1938 | 0.0007 | 0.6601 | 0.8489 | 2.3936 | 0.0082 | 0.1229 | 0.8598 | 4.2153 | 0.0142 | 0.0409 | 0.4557 |  | 298 |  |
| Superior parietal cortex | 2.7978 | 0.0095 | 0.0955 | 0.5728 | 0.1228 | 0.0004 | 0.7262 | 0.9601 | 0.2169 | 0.0007 | 0.6418 | 0.9062 |  | 301 |  |
| Superior temporal gyrus | 0.4537 | 0.0018 | 0.5012 | 0.8144 | 0.6334 | 0.0025 | 0.4268 | 0.8598 | 0.8267 | 0.0032 | 0.3641 | 0.9062 |  | 261 |  |
| Supramarginal gyrus | 0.2228 | 0.0008 | 0.6373 | 0.8489 | 0.4905 | 0.0018 | 0.4843 | 0.8598 | 1.4904 | 0.0055 | 0.2232 | 0.8784 |  | 276 |  |
| Frontal pole | 0.1892 | 0.0006 | 0.6639 | 0.8489 | 9.5400 | 0.0315 | 0.0022 | 0.1719 | 1.4486 | 0.0049 | 0.2297 | 0.8784 |  | 301 |  |
| Temporal pole | 0.0662 | 0.0002 | 0.7971 | 0.9300 | 4.7720 | 0.0160 | 0.0297 | 0.7726 | 0.3499 | 0.0012 | 0.5546 | 0.9062 |  | 302 |  |
| Transverse temporal gyrus | 2.2710 | 0.0077 | 0.1329 | 0.6499 | 0.9073 | 0.0031 | 0.3416 | 0.8598 | 0.9207 | 0.0031 | 0.3381 | 0.9062 |  | 302 |  |
| Insula | 0.1085 | 0.0004 | 0.7421 | 0.8911 | 0.0275 | 0.0001 | 0.8684 | 0.9770 | 2.1077 | 0.0075 | 0.1477 | 0.7679 |  | 284 |  |
| Total surface area | 0.0048 | 0.0000 | 0.9447 | 0.9597 | 1.1727 | 0.0040 | 0.2797 | 0.8598 | 3.5013 | 0.0117 | 0.0623 | 0.5400 |  | 301 |  |
|  |  |  |  |  |  |  |  |  |  |  |  |  |  |  |  |

AD: cases with current AD use

**Table S38.** **Secondary analyses for mean cortical thickness of the rostral anterior cingulate cortex: AD type*Age interaction within the sample of the AD group controlling for age, sex and HDRS-17 score.**

|  |  | **AD type*Age interaction** | | | **Number of subjects** | | |
| --- | --- | --- | --- | --- | --- | --- | --- |
|  | F | Partial η^2^ | P-value | P_FDR_ | SNRI | SSRI | Mirta- zapine |
| Rostral anterior cingulate cortex | 6.6721 | 0.0179 | 0.0013 | 0.0013* | 334 | 298 | 110 |

* FDR P-value < 0.05; SNRI: selective serotonin and noradrenalin reuptake inhibitor; SSRI: selective serotonin reuptake inhibitor

**Table S39.** **Secondary analyses for mean cortical thickness of the rostral anterior cingulate cortex: AD type*Age interaction within the sample of the AD group controlling for age, sex and BDI-II score.**

|  |  | **AD type*Age interaction** | | | **Number of subjects** | | |
| --- | --- | --- | --- | --- | --- | --- | --- |
|  | F | Partial η^2^ | P-value | P_FDR_ | SNRI | SSRI | Mirta- zapine |
| Rostral anterior cingulate cortex | 1.5320 | 0.0147 | 0.2186 | 0.2186 | 97 | 91 | 27 |

SNRI: selective serotonin and noradrenalin reuptake inhibitor; SSRI: selective serotonin reuptake inhibitor

**Table S40.** **Secondary analyses for mean cortical thickness of the rostral anterior cingulate cortex: AD type*Age interaction within the sample of the AD group controlling for age, sex and number of depressive episodes.**

|  |  | **AD type*Age interaction** | | | **Number of subjects** | | |
| --- | --- | --- | --- | --- | --- | --- | --- |
|  | F | Partial η^2^ | P-value | P_FDR_ | SNRI | SSRI | Mirta- zapine |
| Rostral anterior cingulate cortex | 4.1021 | 0.0218 | 0.0173 | 0.0173* | 149 | 166 | 63 |

* FDR P-value < 0.05; SNRI: selective serotonin and noradrenalin reuptake inhibitor; SSRI: selective serotonin reuptake inhibitor

**Table S41.** **Secondary analyses for mean cortical thickness of the rostral anterior cingulate cortex: AD type*Age interaction within the sample of the AD group controlling for age, sex and stage of illness.**

|  |  | **AD type*Age interaction** | | | **Number of subjects** | | |
| --- | --- | --- | --- | --- | --- | --- | --- |
|  | F | Partial η^2^ | P-value | P_FDR_ | SNRI | SSRI | Mirta- zapine |
| Rostral anterior cingulate cortex | 4.6418 | 0.0171 | 0.0100 | 0.0100* | 213 | 272 | 58 |

* FDR P-value < 0.05; SNRI: selective serotonin and noradrenalin reuptake inhibitor; SSRI: selective serotonin reuptake inhibitor

**Table S42.** **Secondary analyses for mean cortical thickness of the rostral anterior cingulate cortex: AD type*Age interaction within the sample of the AD group controlling for age, sex and remission status.**

|  |  | **AD type*Age interaction** | | | **Number of subjects** | | |
| --- | --- | --- | --- | --- | --- | --- | --- |
|  | F | Partial η^2^ | P-value | P_FDR_ | SNRI | SSRI | Mirta- zapine |
| Rostral anterior cingulate cortex | 8.0205 | 0.0193 | 0.0004 | 0.0004* | 356 | 354 | 114 |

* FDR P-value < 0.05; SNRI: selective serotonin and noradrenalin reuptake inhibitor; SSRI: selective serotonin reuptake inhibitor
